# Tislelizumab plus lenvatinib in advanced fumarate hydratase-deficient renal cell carcinoma: a single-arm, phase 2 trial with metabolic biomarker analysis

**DOI:** 10.1038/s41467-026-77615-9

**Published:** 2026-09-07

**Authors:** Wen Kong, Yunze Xu, Guiqin Liu, Wenwen Lv, Zaoyu Wang, Dengfeng Cao, Feng Jiao, Jiong Hu, Xiaoyu Wu, Jiwei Huang, Yonghui Chen, Wei Zhai, Xiaorong Wu, Jiahua Pan, Yiran Huang, Guangyu Wu, Liang Zheng, Wei Xue, Jin Zhang

**Affiliations:** 1 Department of Urology, Renji Hospital, Shanghai Jiao Tong University School of Medicine, Shanghai, China; 2 Department of Radiology, Renji Hospital, Shanghai Jiao Tong University School of Medicine, Shanghai, China; 3 Clinical Research Center, Shanghai Jiao Tong University School of Medicine, Shanghai, China; 4 Department of Pathology, Renji Hospital, Shanghai Jiao Tong University School of Medicine, Shanghai, China; 5 Department of Medical Oncology, Renji Hospital, Shanghai Jiao Tong University School of Medicine, Shanghai, China; 6 Pediatric Translational Medicine Institute, Shanghai Children’s Medical Center, Shanghai Jiao Tong University School of Medicine, Shanghai, China

**Keywords:** Renal cancer, Cancer immunotherapy

## Abstract

Fumarate hydratase-deficient renal cell carcinoma (FH-RCC) is a rare, aggressive kidney cancer with no established standard of care. This phase 2 trial (NCT05877820) assessed first-line tislelizumab plus lenvatinib in advanced FH-RCC (*N* = 20). The primary endpoint was objective response rate (ORR); secondary endpoints included progression-free survival (PFS), overall survival (OS), disease control rate (DCR), duration of response (DOR) and safety. After a median follow-up of 20.2 months, ORR was 95% (95% CI 75.1–99.9), and DCR was 100% (95% CI 83.2–100). Median PFS was 20.7 months (95% CI 17.8–NR), median DOR was 19.2 months (95% CI 16.3–NR) and 2-year OS rate was 89.7% (95% CI 77.2–100). Grade ≥3 treatment-related adverse events occurred in 45% of patients. Circulating succinyl-adenosine and succinic-cysteine tracked radiographic tumor burden in 80% of patients. Limitations include the single-center, single-arm design, and limited sample size. These results support tislelizumab plus lenvatinib as a potential first-line option for advanced FH-RCC, with favorable antitumor activity and manageable safety profile. Circulating succinate-modifying metabolites may complement response monitoring but require further validation.

## Abstract (web summary)

Effective first-line treatments for fumarate hydratase-deficient renal cell carcinoma remain poorly defined, despite promising activity of immune checkpoint inhibitor– based combinations. There these authors report efficacy outcomes of a phase 2 clinical trial on tislelizumab plus lenvatinib in fumarate hydratase-deficient renal cell carcinoma patients.

## Introduction

Fumarate hydratase-deficient renal cell carcinoma (FH-RCC) is a rare renal malignancy with aggressive biological behavior and a dismal prognosis. FH-RCC is mostly associated with hereditary leiomyomatosis and renal cell carcinoma (HLRCC) syndrome characterized by *FH* germline mutations; while it is also caused by biallelic somatic *FH* alterations in few cases^1^. Tumorigenesis and progression of FH-RCC are believed to be driven by tricarboxylic acid metabolic rewiring and abnormal accumulation of fumarate in the tumor cell, due to functional deficiency of fumarate hydratase^2^.

There is no standard of care for patients with advanced FH-RCC, although the NCCN Guidelines recommend bevacizumab plus erlotinib (Beva/Erlo) based on a phase 2 trial initiated over ten years ago^3,4^, the results of which were recently published^5^. During the past decade, combinations of immune checkpoint inhibitor (ICI) and tyrosine kinase inhibitor (TKI) have been shown to provide great benefit for patients with advanced RCC and revolutionized the treatment landscape^6–9^. A real-world multicenter retrospective study showed comparable response rates between ICI/TKI and Beva/Erlo in the front-line treatment for advanced FH-RCC patients, and the former seemed to provide favorable clinical outcomes^10^. However, the retrospective nature and different TKIs used in the study population obscured the advantages of ICI/TKI therapy. Prospective KEYNOTE-B61 study demonstrated that pembrolizumab plus lenvatinib had encouraging antitumor efficacy and acceptable safety for advanced non-clear cell RCC patients, yet without focusing on any specified subtype, such as FH-RCC^11^. Here, we report the results of a prospective phase 2 study of tislelizumab plus lenvatinib combination as the first-line therapy for patients with advanced or metastatic FH-RCC. Tislelizumab is an anti-PD-1 monoclonal antibody developed by a Chinese biopharmaceutical company and is approved for the treatment of multiple malignancies across China and Western countries.

The disrupted Kreb’s cycle caused by FH deficiency occurring in the tumor cell leads to unique metabolic traits and our previous work identified two robust circulating metabolic biomarkers, succinyl-adenosine and succinic-cysteine, which were able to sensitively and specifically reflect genomic alteration status and tumor burden in FH-RCC^12^. In parallel, as an exploratory endpoint, we also report dynamic correlation between biomarker change and radiological tumor response.

In this work, we show that tislelizumab plus lenvatinib has favorable antitumor activity in patients with previously untreated advanced FH-RCC, with a safety profile consistent to that of previous reports, which shows potential as a first-line treatment option. Circulating succinate-modifying metabolites may act as potentially complementary biomarkers for real-time treatment response monitoring but require further prospective validation.

## Results

### Baseline characteristics

**Fig. 1:**
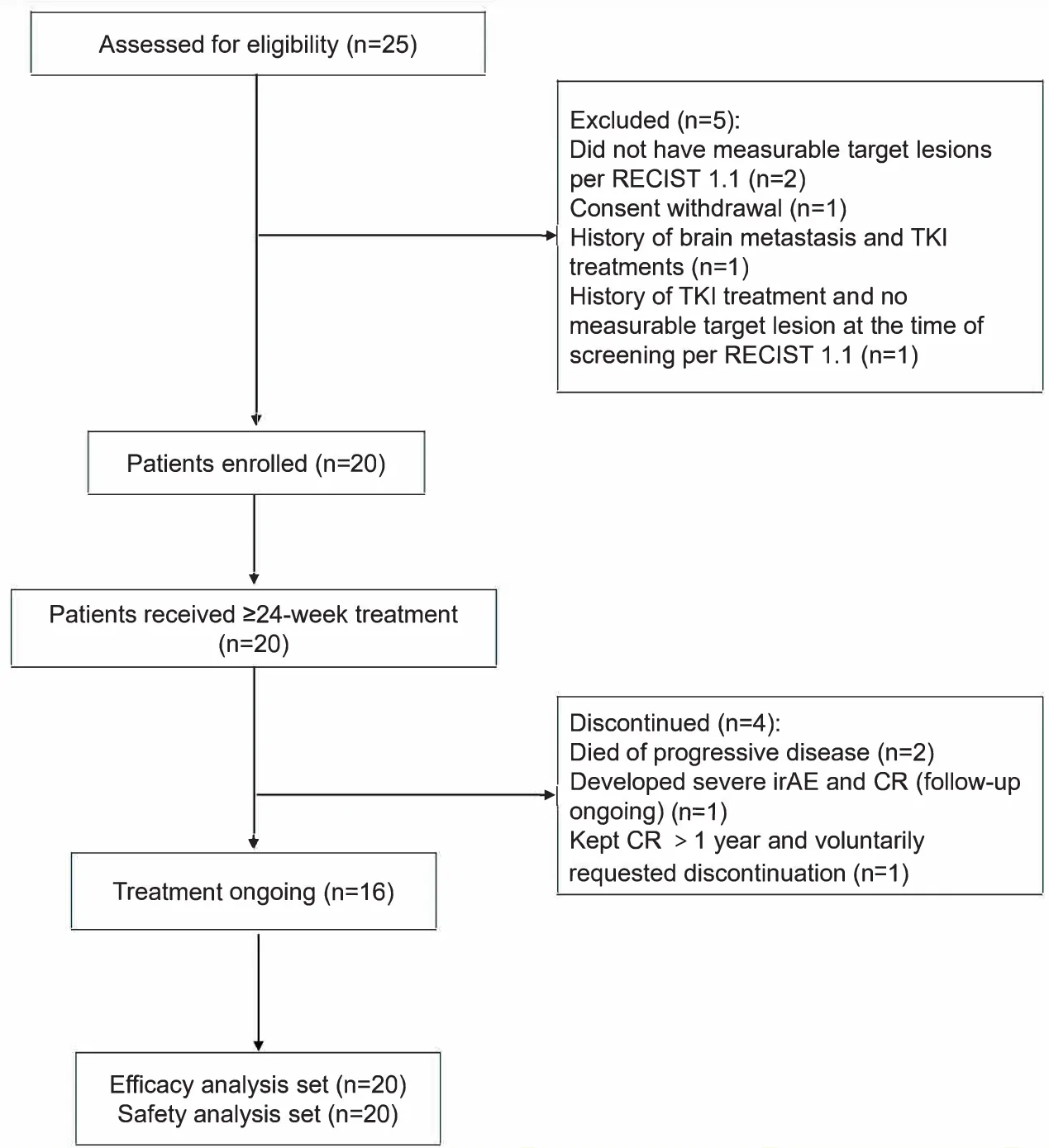
Trial profile. A total of 25 patients provided informed consent. 5 patients were excluded before treatment initiation, including 2 patients who did not have measurable target lesions per RECIST 1.1; 1 patient who withdrew his informed consent; 1 patient with history of brain metastasis and TKI treatment and 1 patient who had history of TKI treatment and without measurable target lesion at the time of screening. A total of 20 patients were enrolled and all received treatment over 24 weeks, which comprised the safety set and efficacy set. At the data cutoff (December 22, 2025), 4 patients discontinued treatment because of progressive disease, death, adverse events, or patient voluntary request.

From September 5, 2023, to December 10, 2024, 25 patients were screened for eligibility, and 20 patients were enrolled (Fig. 1). At the data cutoff on December 22, 2025, the median follow-up for survival was 20.2 months (95%CI 14.6–23.9), estimated using the reverse Kaplan-Meier method. Patient characteristics are summarized in Table 1 and individual details are listed in supplementary source data. Median age at enrollment was 41.5 years (IQR: 30-50); 16 (80%) patients were male and 4 (20%) were female. Physical status was affected by advanced disease in 8 (40%) patients, whose ECOG performance status was 1 or 2. Patients were classified as favorable, intermediate, or poor risk group in 3 (15%), 11 (55%), and 6 (30%) patients respectively per International Metastatic RCC Database Consortium (IMDC) risk categorization. Twelve patients (60%) had a history of radical nephrectomy, and 3 (15%) underwent partial nephrectomy prior to enrollment. For cases with a history of nephrectomy, the median interval from surgery until enrollment was 10.7 months (IQR: 5.7–14.2). The median number of involved organs or disease sites was 3 (IQR: 1–4). The most common locations of metastases or recurrence were lymph node (14/20, 70%), followed by bone (10/20, 50%), retroperitoneum/renal fossa/peritoneum (10/20, 50%), adrenal gland (6/20, 30%), lung (4/20, 20%), and liver (3/20, 15%). Regarding pathology, tumor tissue from 19 patients was available for immunohistochemical staining, amongst whom 16 had a confirmed lack of FH expression, while the remaining 3 showed FH positivity. In contrast, all tumor tissues demonstrated positive staining for 2-succinocysteine (2-SC). On the molecular level, *FH* germline mutation was identified in 15 out of 20 patients (75%), while the other 5 (25%) were confirmed carrying somatic biallelic *FH* alterations (Table 1).

**Table 1:** Baseline clinical characteristics of enrolled patients

Characteristics	Cohort (*n* = 20)
Median (range) age, years	41.5 (24–62)
Sex, No. (%)	
Male	16 (80)
Female	4 (20)
ECOG performance status, No. (%)	
0	12 (60)
1	4 (20)
2	4 (20)
IMDC risk classification, No. (%)	
Favorable	3 (15)
Intermediate	11 (55)
Poor	6 (30)
Prior nephrectomy, No. (%)	
Partial nephrectomy	3 (15)
Radical nephrectomy	12 (60)
T stage at enrollment, No. (%)	
Tx	12 (60)
T1-T2	2 (10)
≥T3	6 (30)
No. of disease sites, Median (range)	3 (1−6)
Location of metastasis, No. (%)	
Lymph nodes	14 (70)
Bone	10 (50)
Retroperitoneum /Peritoneum/Residual kidney recurrence	10 (50)
Adrenal gland	6 (30)
Lung	4 (20)
Liver	3 (15)
Molecular pathology, No. (%)	
Germline	15 (75)
Somatic	5 (25)

*ECOG* Eastern Cooperative Oncology Group, *IMDC* International Metastatic RCC Database Consortium.

Source data are provided as a Source Data file.

### Efficacy

**Fig. 2:**
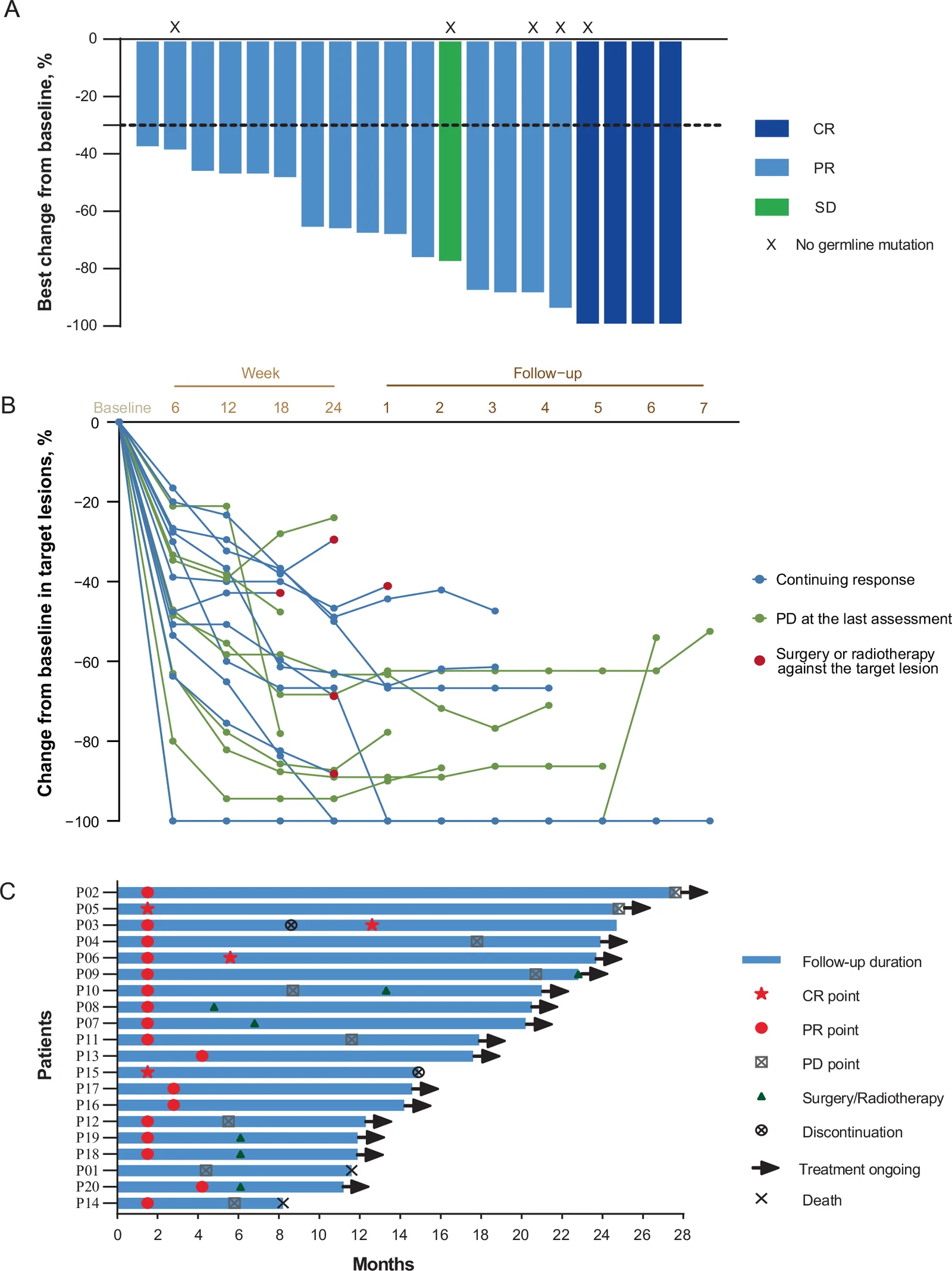
Anti-tumor activity and treatment duration. **A** Waterfall plot of maximal percentage change from baseline in target lesions and best response according to RECIST 1.1. **B** Spider plot of percentage change of target lesions in individual patients from start of treatment. In the initial 24 weeks, imaging evaluations were performed every 6 weeks. In the subsequent follow-up period, imaging evaluations were performed every 3 months or specified by investigators’ judgment (Green line: PD at the last assessment; Blue line: continuing response; Red dot: surgery or radiotherapy against the target lesion in the duration of clinical response). **C** Swimmer plot illustrating the treatment course for each enrolled patient. Bars show the length of drug exposure, while overlaid markers indicate the time points of best overall response, progression or death events, locoregional interventions, and the survival status at data cutoff. Source data are provided as a Source Data file. CR complete response, PR partial response, SD stable disease, PD progressive disease. NR not reached, OS overall survival, PFS progression-free survival.

**Fig. 3:**
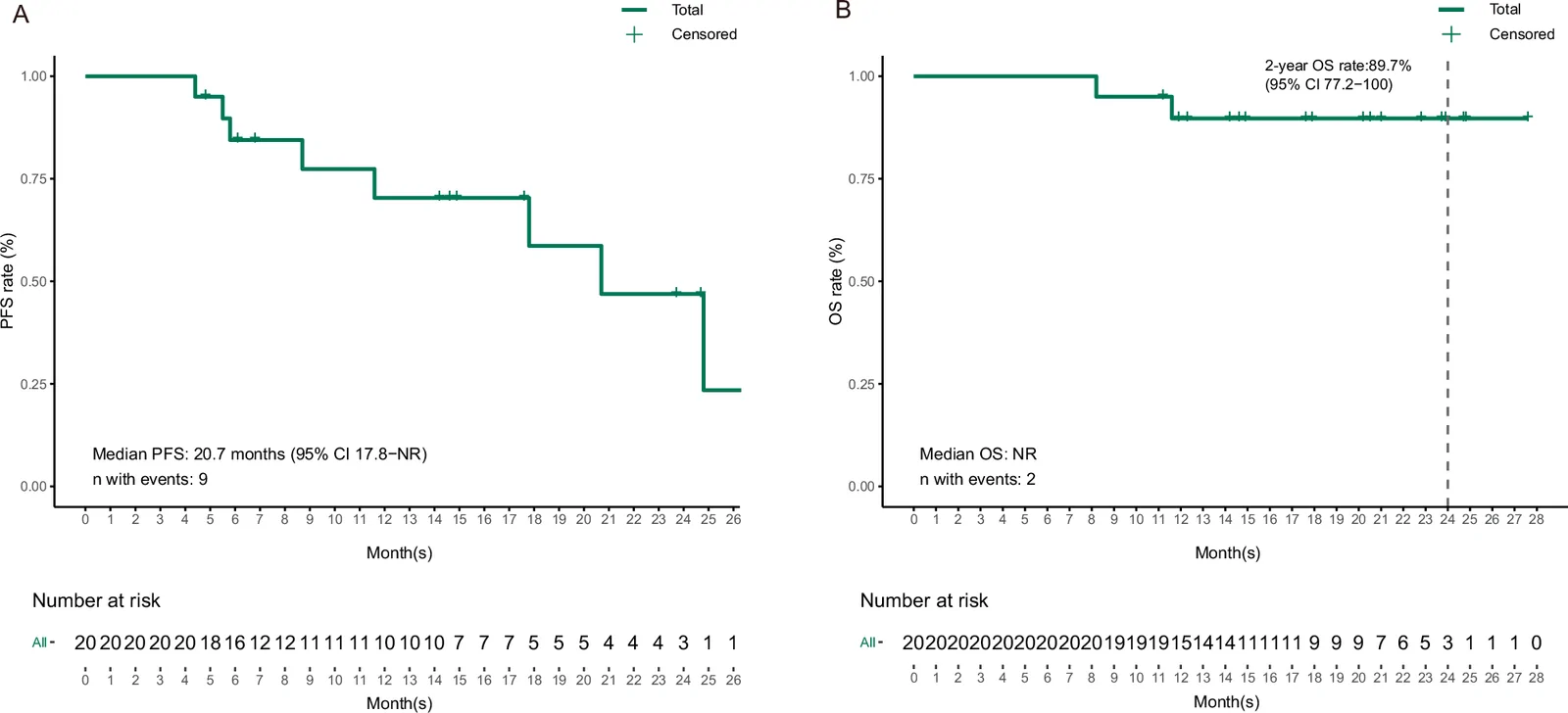
Kaplan–Meier survival curves. **A** Kaplan–Meier survival curve for PFS. Median PFS was 20.7 months (95% CI 17.8-NR). **B** Kaplan–Meier survival curve for OS. Median OS was not reached, and 2-year OS rate was estimated as 89.7% (95% CI 77.2-100). Source data are provided as a Source Data file. CR complete response, PR partial response, PD progressive disease.

Nineteen patients were confirmed to have an objective response, yielding an objective response rate (ORR) of 95%, (95% CI 75.1–99.9), with 4 (20%) achieving complete response (CR) and 15 (75%) showing partial response (PR), while 1 (5%) had stable disease (SD) as the best response by the investigators’ assessment (Fig. 2A). The duration of SD for this patient was 4.4 months, at which point progression of non-target bone metastases was observed (Supplementary Dataset 1). The disease control rate (DCR) was 100% (95% CI 83.2–100) accordingly. The clinical benefit rate, defined as durable disease control lasting ≥24 weeks, was 85% (95% CI 62.1–96.8) (Table 2). When stratified by *FH* status, ORR in patients with *FH* germline mutation seemed higher than that of patients with *FH* somatic alteration (15/15, 100% versus 4/5, 80%), which was not statistically different (Fig. 2A). All patients showed measurable tumor shrinkage since treatment started, and no primary progression was observed (Fig. 2B, Supplementary Dataset 1). Most patients demonstrated rapid clinical response, as the median time to response was 6 weeks (IQR: 6–6). The median duration of response (DOR) was 19.2 months (95% CI 16.3–NR), and the median progression-free survival (PFS) was 20.7 months (95% CI 17.8–NR) (Fig. 3A). Median overall survival (OS) was not reached, while the 2-year OS rate was estimated as 89.7% (95% CI 77.2–100) (Fig. 3B). Among the entire cohort, 9 experienced subsequent disease progression; 7 underwent surgery or radiotherapy against predefined target lesions; 1 patient discontinued the treatment due to serious immune-related adverse event (irAE), 1 patient requested discontinuation after maintenance of CR for 13.4 months, and 2 patients died. At last follow-up, 16 patients remained on combination therapy; 7 were continuing beyond progression per investigator judgment (i.e., after oligoprogression managed with radiotherapy, or limited progression managed with drug re-escalation), 4 maintained an ongoing response, and 5 continued treatment as adjuvant therapy after definitive surgery or radiotherapy to target lesions (Fig. 2C) Although the response rate was not significantly different between patients with *FH* germline mutation and those with somatic alteration, the subgroup with *FH* germline mutation showed numerically favorable median PFS compared with that in patients carrying somatic alteration (24.8 months versus 8.7 months).(Supplementary Fig. S1). Given the small number of patients in the somatic subgroup (*n* = 5), formal statistical comparisons are underpowered and should be interpreted as hypothesis-generating only. The *p*-values are exploratory and highly sensitive to individual patient outcomes and therefore not reported.

**Table 2:** Treatment efficacy

Response	Cohort (*n* = 20)
ORR, % (95% CI)	95 (75.1–99.9)
Best response, No. (%)	
CR	4 (20)
PR	15 (75)
SD	1 (5)
PD	0 (0)
Disease control rate, % (95% CI)	100 (83.2–100)
Clinical benefit rate, % (95% CI)	85 (62.1–96.8)
Median TTR, weeks (range)	6 (6-18)
Median DOR, months, (95% CI)	19.2 (16.3-NR)
Median PFS, months, (95% CI)	20.7 (17.8-NR)
Median OS, months	NR
2-year OS rate, % (95% CI)	89.7 (77.2–100)

NOTE. ORR includes patients with CR or PR. DCR includes patients with objective response or SD in the initial 24 weeks. Clinical benefit includes patients with objective response or SD lasting ≥ 24 weeks.

*TTR* time to response, *DOR* duration of response, *ORR* objective response rate, *PD* progressive disease, *PFS* progression-free survival, *PR* partial response, *SD* stable disease, *NR* not reached.

Source data are provided as a Source Data file.

### Safety

At the data cutoff, the median duration of treatment with at least one agent was 16.3 months (IQR: 11.9–21.5). All patients experienced treatment-related adverse events (TRAEs); grade ≥3 AEs occurred in 9 (45%). The most common TRAEs of any grade included hypertension (80%), TSH elevation/hypothyroidism (80%), proteinuria (70%), pharyngolaryngeal pain (45%), and hyperuricemia (40%). The most common grade 3 TRAEs were hypertension (30%) and arthralgia (15%). No grade 4 or higher TRAEs occurred (Table 3). Lenvatinib was temporarily held or was reduced with the dosage in 13 patients (65%) for AEs management and the median number of breaks was 1 (range: 0–2). One patient permanently discontinued lenvatinib due to grade 3 immune-related pneumonitis and tumor complete remission during the following assessment. The most frequent TRAEs causing lenvatinib interruption or dosage reduction were arthralgia (6/20, 30%), pharyngolaryngeal pain (5/20, 25%), palmar-plantar erythrodysesthesia (4/20, 20%), transaminitis or hyperbilirubinemia (4/20, 20%) and thrombocytopenia (2/20, 10%). At the latest follow-up, lenvatinib of 20 mg per day remained in 4 patients, 2 patients kept on 16 mg per day, while 10 patients reduced to 12 or 8 mg per day. Dose re‑escalation of lenvatinib (from 8 mg to 12 mg) was performed in 2 patients (10%) due to imaging‑documented lesion fluctuation after initial dose reduction for grade 3 arthralgia or intolerable grade 2 palmar‑plantar erythrodysesthesia. Tislelizumab was temporarily held in 3 patients (15%) due to irAEs (pneumonitis in 2 patients, adrenal insufficiency in 1 patient). High‑dose steroids (≥40 mg prednisone equivalent) were required in both patients with pneumonitis, one permanently discontinued tislelizumab because of severe immune‑related pneumonitis and the other one recovered after steroid therapy and resumed immunotherapy thereafter. One patient with adrenal insufficiency required hydrocortisone supplement.

**Table 3:** Treatment related adverse events

AE	All grades, No (%)	Grade≥3, No (%)
Any	20 (100)	9 (45)
Hypertension	16 (80)	6 (30)
TSH elevation/Hypothyroidism	16 (80)	0 (0)
Proteinuria	14 (70)	1 (5)
Pharyngolaryngeal pain	9 (45)	1 (5)
Hyperuricemia	8 (40)	0 (0)
Diarrhea	7 (35)	0 (0)
Sinus tachycardia	7 (35)	0 (0)
Palmar-plantar erythrodysesthesia	7 (35)	0 (0)
Tooth pain	6 (30)	0 (0)
Nausea	6 (30)	0 (0)
Arthralgia	6 (30)	3 (15)
Fatigue	5 (25)	1 (5)
Eosinophilia	5 (25)	0 (0)
Electrocardiogram ST-T change	5 (25)	0 (0)
Hyperthyroidism	4 (20)	0 (0)
Rash acneiform	4 (20)	0 (0)
Neutropenia	4 (20)	0 (0)
Nail disorder	3 (15)	0 (0)
Hyperbilirubinemia	3 (15)	0 (0)
Serum creatinine elevation	3 (15)	0 (0)
Thrombocytopenia	3 (15)	0 (0)
Lymphopenia	3 (15)	0 (0)
Insomnia	3 (15)	0 (0)
Transaminitis	3 (15)	0 (0)
Immune-related pneumonitis	2 (10)	1 (5)
Fever	2 (10)	0 (0)
Vomiting	2 (10)	0 (0)
Hypertriglyceridemia	2 (10)	0 (0)
Urinary tract infection	2 (10)	0 (0)
Epistaxis	2 (10)	0 (0)
Mucositis oral	1 (5)	0 (0)
Gastroesophageal reflux	1 (5)	0 (0)
Lung infection	1 (5)	0 (0)
Immune-related adrenal insufficiency	1 (5)	0 (0)
Facial nerve disorder	1 (5)	1 (5)
Cardiac troponin T elevation	1 (5)	0 (0)
Cardiac failure	1 (5)	1 (5)
Nail disorder	1 (5)	0 (0)
Atrioventricular block	1 (5)	0 (0)

### Metabolic biomarker analysis

Our previous work using the mass spectrometry platform identified two tumor-derived metabolites, succinyl-adenosine and succinic-cysteine, as plasma biomarkers for diagnosis of tumors with FH deficiency, with cutoff values of 12 ng/mL and 32 ng/mL, respectively. Moreover, correlation between these metabolites and tumor burden was observed, supporting their potential relevance for monitoring response to systemic therapies^12^.

**Fig. 4:**
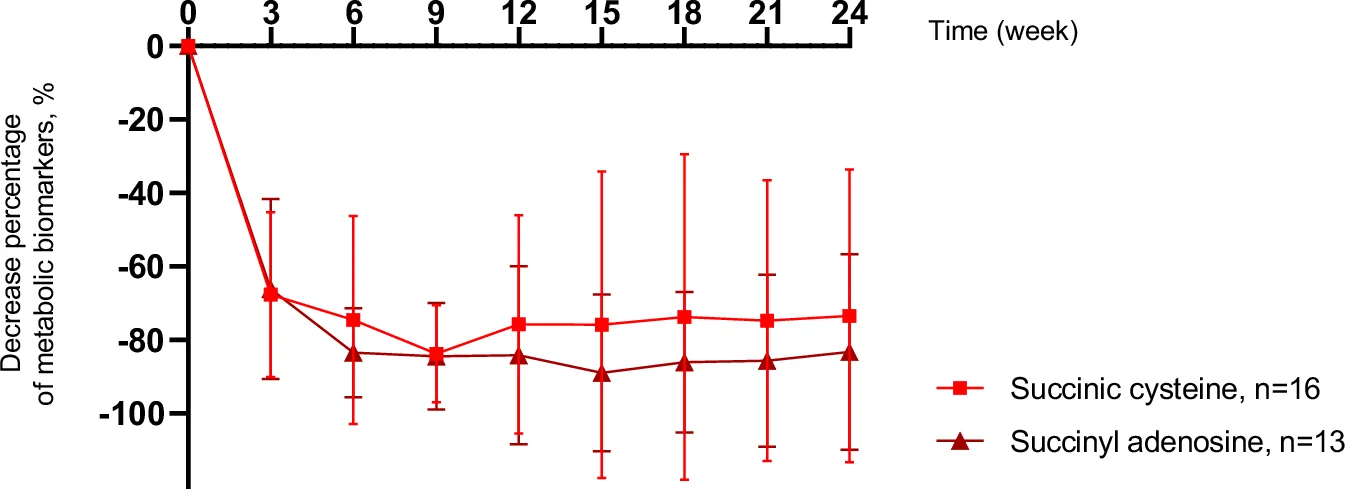
Dynamics of circulating succinate-modifying metabolites during the initial 24 weeks in the qualified set. Serial blood samples were obtained every 3 weeks from patients participating in the exploratory endpoint study, and measurements of succinyl‑adenosine and succinyl‑cysteine concentrations were performed via liquid chromatography-mass spectrometry quantitation. The plotted values are the average percentage changes at each scheduled visit. For succinyl-adenosine, the qualified sample size *n* = 13, and for succinic-cysteine, the qualified sample size *n* = 16. Specifically, n represents the number of independent patients, and all statistical analyses were derived from biological replicates. In this figure, the error bars represent standard deviation SD, and the central values indicate the mean value. Source data are provided as a Source Data file.

**Fig. 5:**
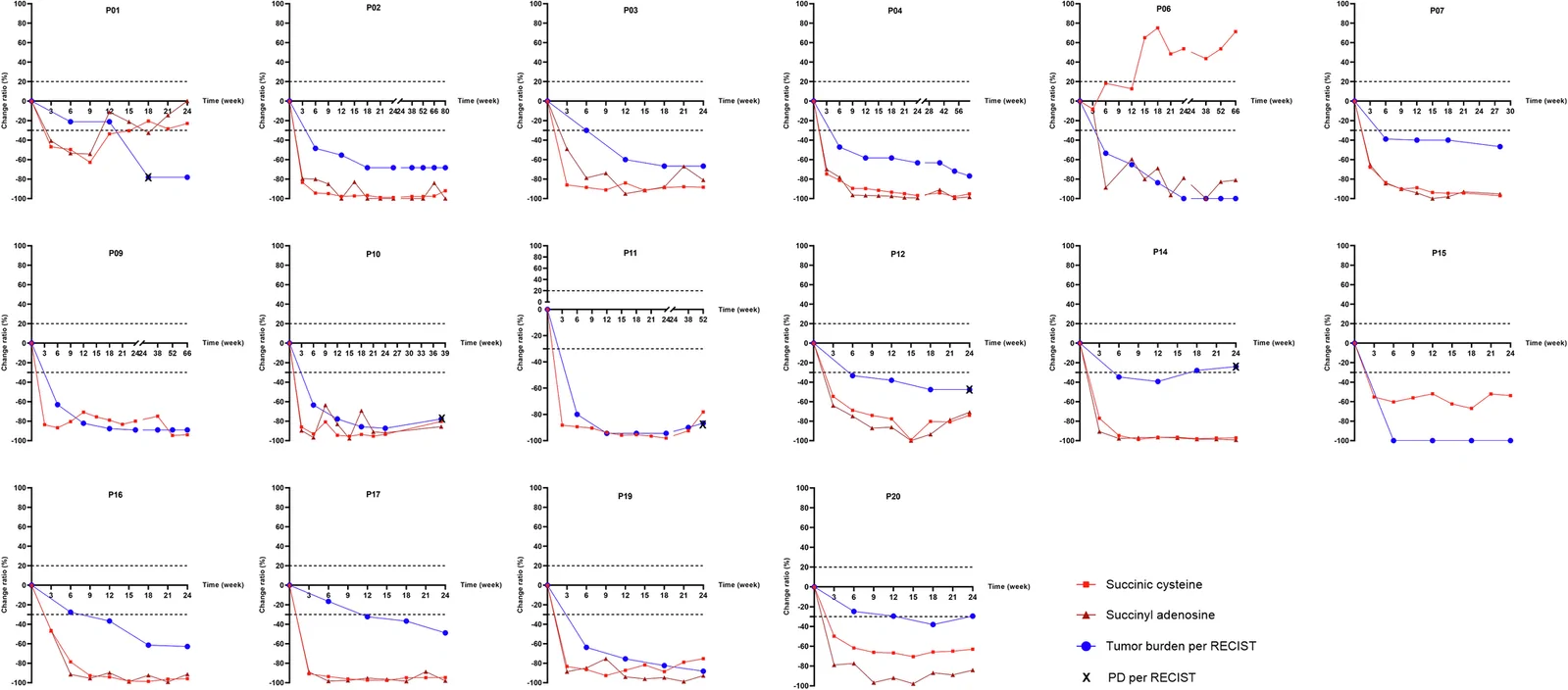
Change percentage of circulating succinate-modifying metabolites and tumor burdens as per RECIST assessment for each patient. The blue plot depicts the longitudinal percentage change of RECIST target lesions from the baseline at every time point of imaging assessment. The light and dark red plots depict the longitudinal percentage change of circulating succinic cysteine and succinyl adenosine respectively from the baseline at every time point of follow-up. The dark cross depicts the first documented disease progression per RECIST assessment (For patients P01, P10, P11, P12 and P14, PD was recorded along with available circulating metabolites quantitation results; For patient P02, P04, P05 and P09, PD were recorded at the time when metabolic biomarker data were not available). Source data are provided as a Source Data file.

All enrolled patients participated in the exploratory analysis of biomarker dynamics versus radiological response. At baseline, 4 patients had both biomarkers below the predefined cutoffs and therefore were excluded from longitudinal change analysis to avoid unreliability (Supplementary Fig. S2). All these 4 patients carried low tumor burden, as evidenced by the median number of disease sites being 1 (range: 1–2), in contrast to a median of 3 sites among the entire cohort. Although the median time to clinical response as per imaging assessment was 6 weeks, the circulating succinyl-adenosine and succinic-cysteine levels showed a trend of earlier drop after treatment initiation at Week 3 (Fig. 4). In the remaining 16 patients, we observed coordinated change between circulating metabolite levels and tumor burden depicted via Response Evaluation Criteria in Solid Tumors (RECIST) assessment. In most responders with CR or PR as the best response, at least one of the two metabolic biomarkers dropped by over 80% within the initial 6 weeks, suggesting that metabolic response might occur before imaging manifestations. Based on available data of metabolic quantification, among 4 out of 9 patients with progression disease (PD) during follow-up, visual inspection of serial biomarkers and imaging plots suggested that at least one biomarker elevation occurred temporally before radiographic progression. However, our study was unable to provide formal lead time estimates, false-positive or false-negative rates, time-dependent sensitivity or specificity, or landmark analysis due to the small sample size and exploratory nature of this analysis (Fig. 5).

## Discussion

To our knowledge, this represents one of the few prospective clinical trials conducted exclusively in patients with advanced FH-RCC, adding new evidence to support the use of ICI/TKI combination therapy in the front-line setting. The first-line tislelizumab plus lenvatinib demonstrated encouraging antitumor activity, with 95% of patients having an objective response and 20% achieving complete remission. Notably, we did not observe primary progression in any subject because all patients achieved at least SD during the initial 12 weeks, and all showed some degree of tumor shrinkage (Supplementary Dataset 1). Furthermore, we observed durable treatment efficacy, as the median DOR and median PFS were 19.2 months and 20.7 months, respectively, and the 2-year OS rate was estimated as 89.7%. The activity of tislelizumab plus lenvatinib remained consistent across different IMDC risk groups. The safety profile of the regimen was consistent with previous reports for ICI/TKI combinations. TRAEs of any grade occurred in all patients; most of them were managed and resolved with interruption and dose reduction, except one with permanent discontinuation of both agents.

Due to the low incidence and challenges in obtaining a timely diagnosis, the standard of care for patients with advanced FH-RCC has not been established until now. The AVATAR study evaluated the efficacy of Beva/Erlo in patients with advanced HLRCC-associated or sporadic papillary RCC and results were recently published^5^. Seventy-two percent of patients with HLRCC-associated RCC exhibited an objective response. The median PFS was 21.1 months, and the median OS was 44.6 months^4,5^. This study was initiated a decade ago and had over five years of follow-up, during which molecular pathology of RCC developed rapidly, and the therapeutic landscape was completely revolutionized by immunotherapy. Previous studies have shown that FH‑RCC typically exhibits abundant lymphocyte infiltration and positive PD‑L1 expression, suggesting that FH-RCC is a highly immunogenic tumor, and these patients are likely to benefit from immunotherapy^10,13,14^. We performed a retrospective multicenter study across China, and for the first time compared the clinical activity of ICI/TKI versus Bev/Erlo combination for advanced FH-RCC patients in the first-line setting. A comparable response rate was observed between ICI/TKI and Bev/Erlo cohort (27% versus 25%), while ICI/TKI seemed to have a favourable median PFS compared to the Beva/Erlo group (18.0 months versus 10.0 months)^10^. Meanwhile, cabozantinib plus nivolumab and pembrolizumab plus lenvatinib combinations were reported to demonstrate evident superiority over previously reported regimens for advanced non-clear cell RCC, in which ORR for the cohort including papillary, unclassified, and translocation-associated RCC achieved 47.5% and 49%, median PFS were 12.5 months and 18 months, respectively^11,15^. Interestingly, cabozantinib plus nivolumab study found that 10 out of 12 patients with either *FH* or *NF2* mutations showed a clinical response, with an ORR significantly higher than the average^15,16^. Similarly, another ICI/TKI combination, sintilimab plus axitinib, showed encouraging antitumor efficacy for advanced FH-RCC patients in a recently published study, in which ORR was 56% with a median duration of response not yet reached and a median PFS of 19.8 months^17^. Although some other retrospective studies showed conflicting results, in which mTOR/VEGF combination or TKI monotherapy had superior efficacy^18,19^, our results, together with sintilimab plus axitinib study, support ICI/TKI combination therapy as potentially another option in first-line treatment for FH-RCC patients. Both sintilimab plus axitinib trial and our study showed numerically higher ORR and longer PFS in patients with germline mutations compared to those with somatic alterations (sintilimab/axitinib: ORR 63% versus 43%, median PFS 32.0 versus 12.6 months; tislelizumab/lenvatinib: ORR 100% versus 80%, median PFS 24.8 versus 8.7 months). However, the sample sizes of these two studies were limited, and definitive conclusions cannot be drawn. Regarding the numerically different response rates observed between tislelizumab plus lenvatinib versus sintilimab plus axitinib combinations, we assume it is probably attributable to the distinct action mechanism of specific targeted therapies. Lenvatinib not only has broader activity to block tumor angiogenesis but also acts directly on tumor cells and modulates the tumor microenvironment by inhibiting pathways such as FGFR, which might translate to a favorable response rate especially in patients with non-clear cell RCC^20,21^.

For decades, oncological evaluation in clinical research has relied on the RECIST, which is primarily based on measurement of target lesions on contrast-enhanced cross-sectional imaging^22^. Taking advantage of the robust metabolic rewiring occurring in FH-deficient tumor cells, our collaborators identified succinyl-adenosine and succinic-cysteine as specific biomarkers for FH-RCC patients in the previous work^12^. Theoretically, all FH-deficient lesions, either measurable or not on imaging, have abnormal fumarate accumulation and persistently release succinate-modifying metabolites into circulation. Therefore, circulating levels of succinyl-adenosine and succinic-cysteine would be ideal surrogate markers for tumor burden, and their fluctuation would mirror tumor remission or progression, which would be potential complements for RECIST-based imaging assessment. By synchronously monitoring the circulating levels of succinyl-adenosine and succinic-cysteine along with the preset imaging evaluations, we found that circulating biomarker dynamics paralleled the trend of tumor burden change quantified by RECIST in the majority of cases. In most responders, at least one of the two biomarkers dropped rapidly as the treatment started, which happened earlier than the first post-treatment imaging assessment and usually demonstrated a greater decline. Among patients initially responding to treatment but progressing later, we could find rebound from nadir of at least one biomarker (Fig. 5: P01, P10, P11, P12). Notably, in 2 patients with progression in non-measurable bone metastasis (Fig. 5: P01, P12), a sustained rise in circulating succinyl-adenosine and succinic-cysteine predated imaging-confirmed PD by nine weeks. The above findings suggest that circulating metabolic biomarkers demonstrate potential for real-time treatment response monitoring and early warning of treatment failure. However, our study did not provide formal statistical validation about lead time, false-positive rates, time-dependent sensitivity or specificity, or landmark, because any attempt to calculate such metrics from such a small sample would be statistically futile and potentially misleading. These observations were only hypothesis-generating, not practice-changing. Four patients in our cohort were found to have both circulating succinyl-adenosine and succinic-cysteine below the predefined cutoff values, thereby excluded from the exploratory analysis. These patients were characterized by low disease burden, mainly presenting with local recurrence. The 20% exclusion rate might reflect the current sensitivity limitation of our assay, not a fundamental failure of the biomarker concept. Considering the absence of established tumor marker for FH-RCC, the ability to monitor disease dynamics in 80% of patients using these metabolic biomarkers represents a meaningful step forward, even if the current assay requires further refinement for low-level cases. With future technical advances, such as high-resolution mass spectrometry, or more sensitive detection modes, the limit of quantification can be lowered, and the operational thresholds can be revised downward. This would possibly extend detectability to low-tumor-burden patients and reduce the exclusion rate. In addition, these biomarkers in female patients with treatment-naïve uterine leiomyomas should be interpreted with caution, because we found deviations of circulating succinyl-adenosine or succinic-cysteine from the imaging-assessed tumor burden (Fig. 5: P06, P15; Supplementary Fig. S3). However, the small number of female patients and the absence of quantitative metabolite measurements from leiomyoma tissue preclude any definitive assessment of the confounding effect of uterine leiomyomas. Whether these metabolites are produced by leiomyomas, and if so, to what extent, remains unknown. The biomarker should not be used in isolation but as an adjunct to clinical and radiographic assessment. One more point, in 5 patients with somatic biallelic *FH* alterations, the longitudinal change trends in circulating metabolic biomarkers appeared similar to those in germline mutation carriers, but the small number precludes definitive statistical comparison. Larger studies are needed to clarify whether mutation type influences metabolic biomarker dynamics. Finally, the limited sample size precluded the development of a robust predictive model correlating metabolic biomarker levels with radiographic measurements. However, despite the absence of formal statistical validation, this exploratory analysis provides valuable proof-of-concept evidence for the utility of circulating biomarkers in kidney cancer, a malignancy for which few biomarkers have yet been validated in clinical practice. Our study demonstrated that serial metabolic biomarker monitoring was feasible in this rare cancer and that in the majority of patients, biomarker dynamics could be visualized in parallel with radiographic tumor changes, with some cases suggesting a temporal lead. These observations provide a rationale and a signal for future prospective cohort studies designed to test whether biomarker-guided monitoring improves outcomes. Meanwhile, whether other liquid biopsy biomarkers, such as ctDNA, can guide treatment for patients with FH-RCC, similar to its role in urothelial carcinoma, remains an area requiring further exploration.

In summary, tislelizumab plus lenvatinib demonstrated encouraging antitumor efficacy with toxicities aligned with those of previous studies in patients with advanced FH-RCC in the first-line setting. Our results, together with other similar studies, support the front-line use of ICI/TKI as an alternative to Beva/Erlo. We also found that longitudinal monitoring of circulating succinate-modifying metabolites might offer a valuable complementary dimension to routine imaging assessment particularly for advanced FH-RCC patients, which act as proof-of-concept regarding metabolic biomarkers in kidney cancer. The small sample size, dictated by the rarity of FH-RCC, underlies potential selection bias, precludes robust subgroup analysis regarding mutation types, and limits formal validation of exploratory biomarkers. The single‑arm, open‑label design without a comparator or blinded independent central review may bias efficacy assessment and prevent definitive attribution of the high response rate to the combination. These constraints are inherent to rare‑disease research and should be considered when interpreting the results. Future efforts should prioritize multicenter collaboration to accrue larger cohorts, incorporate blinded independent central review, and prospectively validate the candidate metabolic biomarkers with optimized assays and lead‑time analysis.

## Methods

### Study design and patient eligibility

This is an investigator-initiated, single-arm, single-center, open-label, phase 2 trial. The study protocol was approved by the Renji Hospital Ethics Committee (Approval No. LY2023-056-B) and was registered in the ClinicalTrials.gov with the identifier NCT05877820; the date of preregistration was August 31, 2023. This trial was conducted in compliance with the Declaration of Helsinki and Good Clinical Practice Standards; all patients provided written informed consent.

Eligible patients were aged 18-80 years with pathological confirmed, unresectable advanced or metastatic FH-RCC, which were reviewed by two expert GU pathologists (Z.W., D.C.). An archival tumor tissue or newly biopsy was tested for FH and 2-SC expression via immunohistochemistry, and genetic testing were required for all subjects. FH-RCC was diagnosed based on morphologic features and immunohistochemical result. Morphologically, FH-RCC demonstrates multiple admixed histologic patterns including papillary, tubulopapillary, solid, cystic and cribriform ones and shows focal to extensive eosinophilic macronucleoli with perinucleolar halo^23^. The diagnosis was supported by negative staining for FH and overexpression of 2-SC (diffuse and strong staining). In cases with positive but weak FH staining, the diagnosis was made based on morphologic features with overexpression of 2-SC^24,25^. In one case without tumor tissue pathology (i.e. cystic renal tumor not suitable for puncture biopsy), the diagnosis of HLRCC was based on the finding of *FH* germline mutation. All FH-RCCs were further confirmed with molecular testing which showed pathogenic or likely pathogenic germline or somatic alteration in *FH* gene. All eligible patients were required to have at least one measurable lesion defined by RECIST 1.1^22^, ECOG performance status 0–2, adequate organ function, and no history of prior systemic therapy. Key exclusion criteria included prior therapy with TKI or ICI agents, clinically significant cardiovascular disease or untreated brain metastases. Male and female patients were recruited based on self-reported sex information, with all study outcomes subsequently reported in a sex-disaggregated manner.

No direct financial incentives were provided for participation, and comprehensive trial-related injury insurance was maintained by the sponsor to provide compensation for any AEs or injuries directly associated with the study procedures.

### Treatment and assessment

All enrolled patients received concurrent lenvatinib 20 mg orally once daily plus tislelizumab 200 mg intravenously every three weeks. Treatment continued until disease progression, unacceptable toxicity, or withdrawal of consent. Patients with PD were allowed to continue the treatment if the investigator considered them to still be deriving treatment benefit. Interruption of tislelizumab, or delay for no more than 12 weeks, or permanent discontinuation was permitted to manage irAEs, while dose reduction was not allowed. Dose reduction of lenvatinib from 20 mg daily to 16 mg, 12 mg, or 8 mg daily gradually in sequence based on the previous dose, or temporal interruption, or discontinuation was implemented to manage lenvatinib-related toxicities. Patients receiving a dose reduction would continue on the reduced dose in subsequent cycles. However, while on a reduced dose, if the investigator judge that there would be a risk or possibility of disease progression, and the patient was considered stable for safety on dose reduction and supportive care (i.e., no grade ≥3 AE or intolerable grade 2 AE), lenvatinib dose re‑escalation by one level (i.e., +4 mg, no skipping) was permitted. Each subject was permitted at most one dose re‑escalation. If a patient again developed grade ≥3 or intolerable grade 2 AEs after re‑escalation, they are managed with protocol‑mandated dose interruption, and no further re‑escalation was allowed. Patients who interrupted or discontinued the treatment of either tislelizumab or lenvatinib were permitted to continue the remaining agent in the absence of disease progression.

Abdominal and pelvic contrast-enhanced magnetic resonance imaging (preferred) or contrast-enhanced computed tomography, and chest plain computed tomography were performed at baseline and repeated every six weeks until Week 24, and every three months thereafter. Tumor response was assessed according to RECIST 1.1 by two independent radiologists (G.W., G.L.). To reduce potential assessment bias in this study, a post-hoc independent radiological review was performed. All available baseline and post-baseline imaging data were anonymized and reviewed according to RECIST 1.1 by two independent radiologists who were not involved in patient care or the original response assessment. Discrepancies were resolved by consensus or by adjudication from a third independent radiologist. Clinical and laboratory assessments, including complete blood counting, urinalysis, metabolic panel, endocrine panel and electrocardiograph, were performed every three weeks within the initial 24 weeks, and every three months thereafter. Extra imaging and laboratory examinations were allowed if clinically needed. For patients participating exploratory endpoint studies, liquid chromatography-mass spectrometry quantitation of plasma succinyl-adenosine and succinic-cysteine, as per previously reported protocol^12^ were conducted every three weeks within the initial 24 weeks to explore the correlation between metabolic biomarker dynamics and clinical response per RECIST assessment, which was continued among patients able to provide plasma samples in the follow-up period after the initial 24 weeks.

### Outcomes

The primary endpoint was ORR defined as the percentage of patients with a confirmed CR or PR as the best response according to RECIST 1.1. Secondary endpoints included PFS, OS, DCR, DOR and TRAEs assessment. PFS and OS were calculated using time-to-events methods to account for censoring. PFS was defined as the interval between treatment initiation and the first occurrence of disease progression or death from any cause. Patients without documented progression or death were censored at the date of the last valid tumor assessment. Patients who initiated non-protocol anti-cancer therapy, including surgery or radiotherapy against target lesions, before documented progression were censored at the last valid tumor assessment before the initiation of subsequent therapy. OS was defined as the time from treatment start to death of any cause. DCR was defined as the combined proportion of patients achieving CR, PR or SD. DOR was calculated from the time of the first documented response until the first event of PD or death of any cause. Additionally, the clinical benefit rate was defined as the percentage of patients who achieved sustained disease control for at least 24 weeks. AEs were assessed and graded according to Common Terminology Criteria for Adverse Events (CTCAE) 5.0^26^ and were recorded until 30 days after treatment discontinuation and post-treatment 90 days for serious AEs.

### Statistics analysis

This study used Simon’s minimax two-stage design to evaluate the antitumor activity of tislelizumab plus lenvatinib in patients with advanced FH-RCC^27–29^. The primary endpoint was ORR according to RECIST 1.1. In our previous real-world retrospective cohort, first-line ICI/TKI or Beva/Erlo produced an ORR of approximately 25–30%^10^. We tested H₀: ORR ≤ 30% (p₀=0.30) versus H₁: ORR ≥ 60% (p₁=0.60), representing a clinically meaningful improvement based on available historical data and the expected activity of ICI/TKI combinations in advanced renal cell carcinoma. With a one-sided α of 0.05 and 80% power, Simon’s minimax two-stage design required 17 evaluable patients, with 10 patients in stage 1 and 7 additional patients in stage 2. The study would stop early for futility if 2 or fewer objective responses were observed among the first 10 patients; otherwise, enrollment would continue to the second stage. The regimen would be considered promising if at least 9 objective responses were observed among 17 evaluable patients. This design yielded an exact one-sided type I error rate of 4.02% under p₀=0.30 and a statistical power of 80.08% under p₁=0.60. To account for an anticipated 10–20% attrition rate and ensure operational feasibility, the target enrollment was set at 20 patients.

Efficacy and safety analyses included all patients who received at least one dose of either study agent. ORR, DCR and clinical benefit rate were summarized with exact Clopper–Pearson 95% CIs. Median PFS, OS, and DOR were estimated using Kaplan-Meier methods, with 95% CIs calculated using the Greenwood’s formula. Median follow-up for survival was estimated using the reverse Kaplan-Meier method, in which deaths were censored, and patients alive at last follow-up were treated as events. The reverse Kaplan‑Meier curve was generated, and the median follow‑up time was defined as the time at which the reverse survival function reached 0.5. All analyses were performed using R software (version 4.3.3).

## Supplementary information

Supplementary Information

Description of Additional Supplementary Files

Supplementary Dataset 1

Transparent Peer Review file

## Source data

Source Data

## Data Availability

The study protocol and all datasets generated during this study are included in the Article, Supplementary Information, and Source Data files. All source data supporting the findings of this study, including the raw data underlying the statistical tables, figures, de-identified patient baseline clinical characteristics, and longitudinal tumor assessment datasets, are provided as Source Data Excel files. To protect participant privacy and in compliance with the requirements of our institutional ethics committee, the individual-level raw clinical data are not deposited in a public repository, as the combination of temporal and demographic variables could potentially allow for participant re-identification. Nevertheless, the complete de-identified aggregated source datasets provided in the supplementary Source Data are fully sufficient to reproduce all statistical analyses and graphical results reported in this manuscript. The remaining raw data is not publicly available due to patient privacy protection laws. Requests for access to the full individual-level de-identified participant data for non-commercial research purposes may be sent to W.K. (kongwen@renji.com) and J.Z. (zhangjin@renji.com) and will be considered on a case-by-case basis and typically will be processed within three months. The data will be available for ten years following publication. Source data are provided with this paper.
