# Supplementary material for: Tislelizumab plus lenvatinib in advanced fumarate hydratase-deficient renal cell carcinoma: a single-arm, phase 2 trial with metabolic biomarker analysis
Source: Nat Commun. 2026 Sep 7;17:10591. doi: 10.1038/s41467-026-77615-9 (PMC13645792; doi:10.1038/s41467-026-77615-9)
Supplement: Supplementary file 1 [file 41467_2026_77615_MOESM1_ESM.pdf]

**Supplementary Information**

Tislelizumab plus lenvatinib in advanced fumarate hydratase-deficient renal cell carcinoma: a single-arm, phase 2 trial with metabolic biomarker analysis

**Supplementary Note 1:**

**Supplementary Figure S1.**

**Supplementary Figure S2.**

**Supplementary Figure S3.**

**Supplementary Note 2:** Study Protocol

**Supplementary Note 3:** CONSORT checklist

## Supplementary Note 1

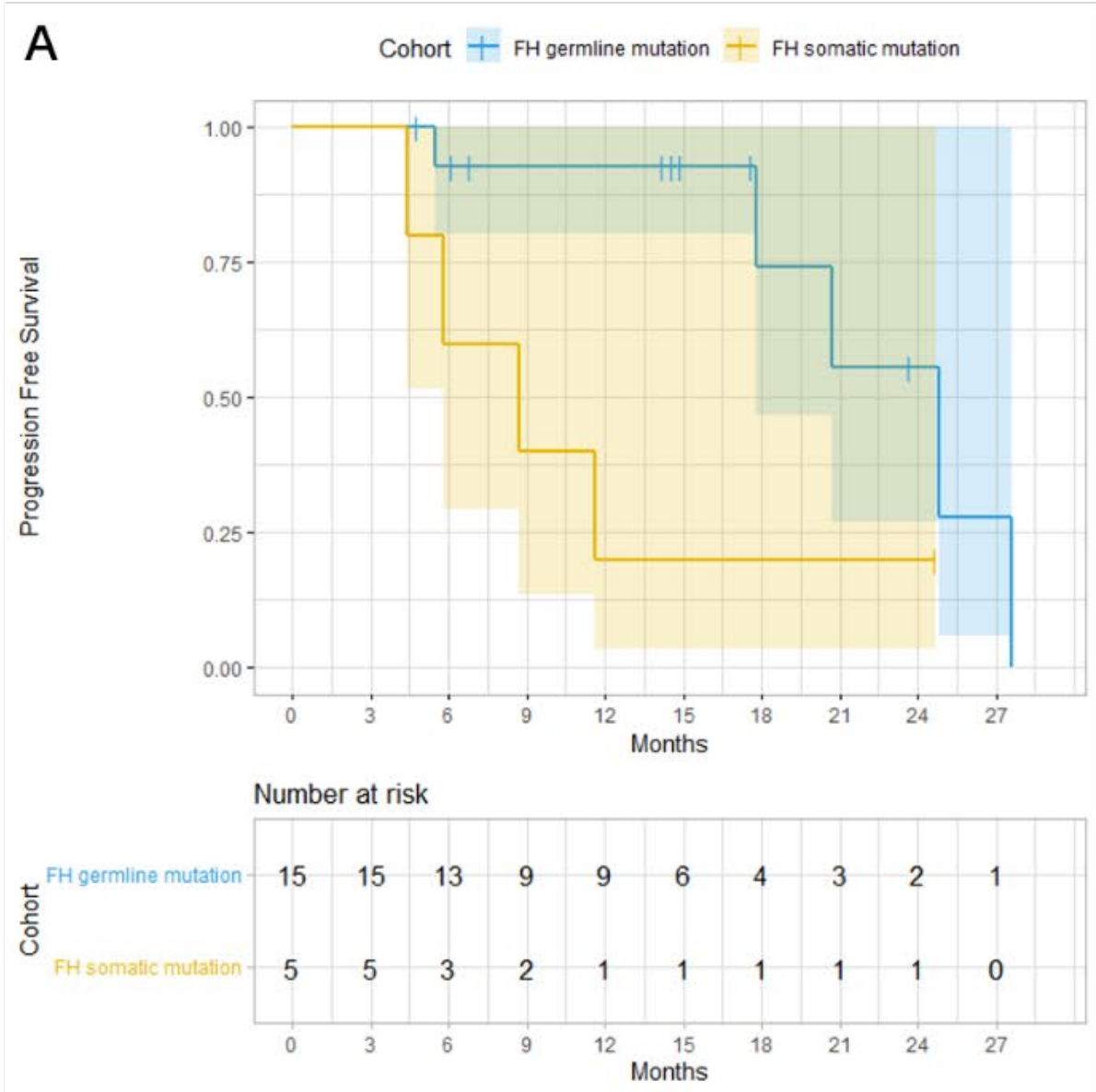


| | FH germline mutation | FH somatic mutation |
|---|---|---|
| Median PFS (month) | 24.8 | 8.7 |

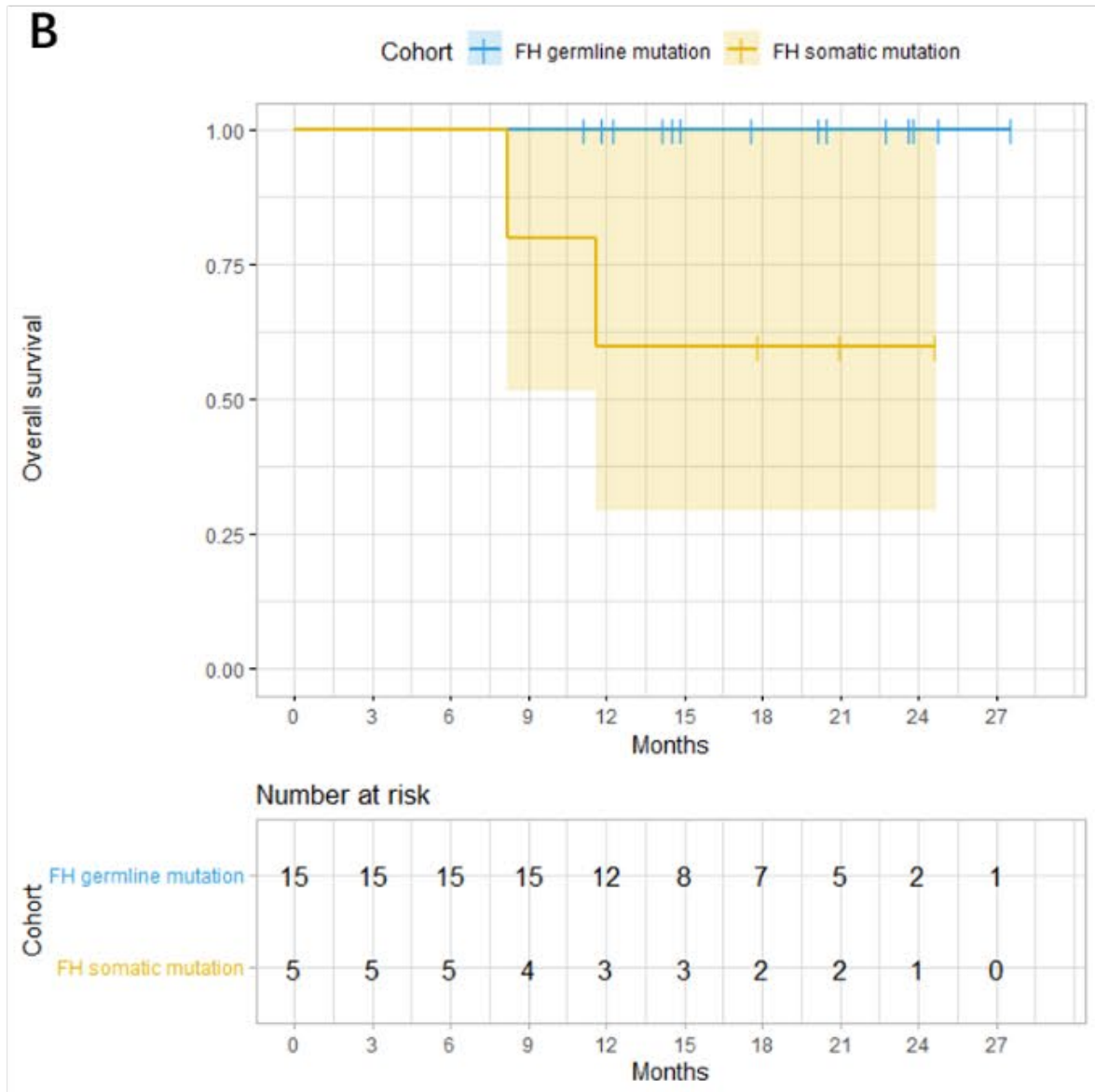


| | FH germline mutation | FH somatic mutation |
|---|---|---|
| Median OS (month) | NR | NR |

**Supplementary Fig. S1.** Comparison of progression-free survival (PFS) and overall survival (OS) between subgroups with different FH status. (A) Kaplan–Meier survival curves for PFS. Median PFS of patients with *FH* germline mutation (n=15) and somatic mutation (n=5) was 24.8 months *vs.* 8.7 months respectively. Given the small number of patients in the somatic subgroup (n=5), formal statistical comparison is underpowered, and the p-value is exploratory and highly sensitive to individual patient outcomes and therefore not reported. (B) Kaplan–Meier survival curves for OS, and median OS for both two subgroups were not reach. Similarly, given the small number in the somatic subgroup (n=5), formal statistical comparison is underpowered, and the p-value is not reported.

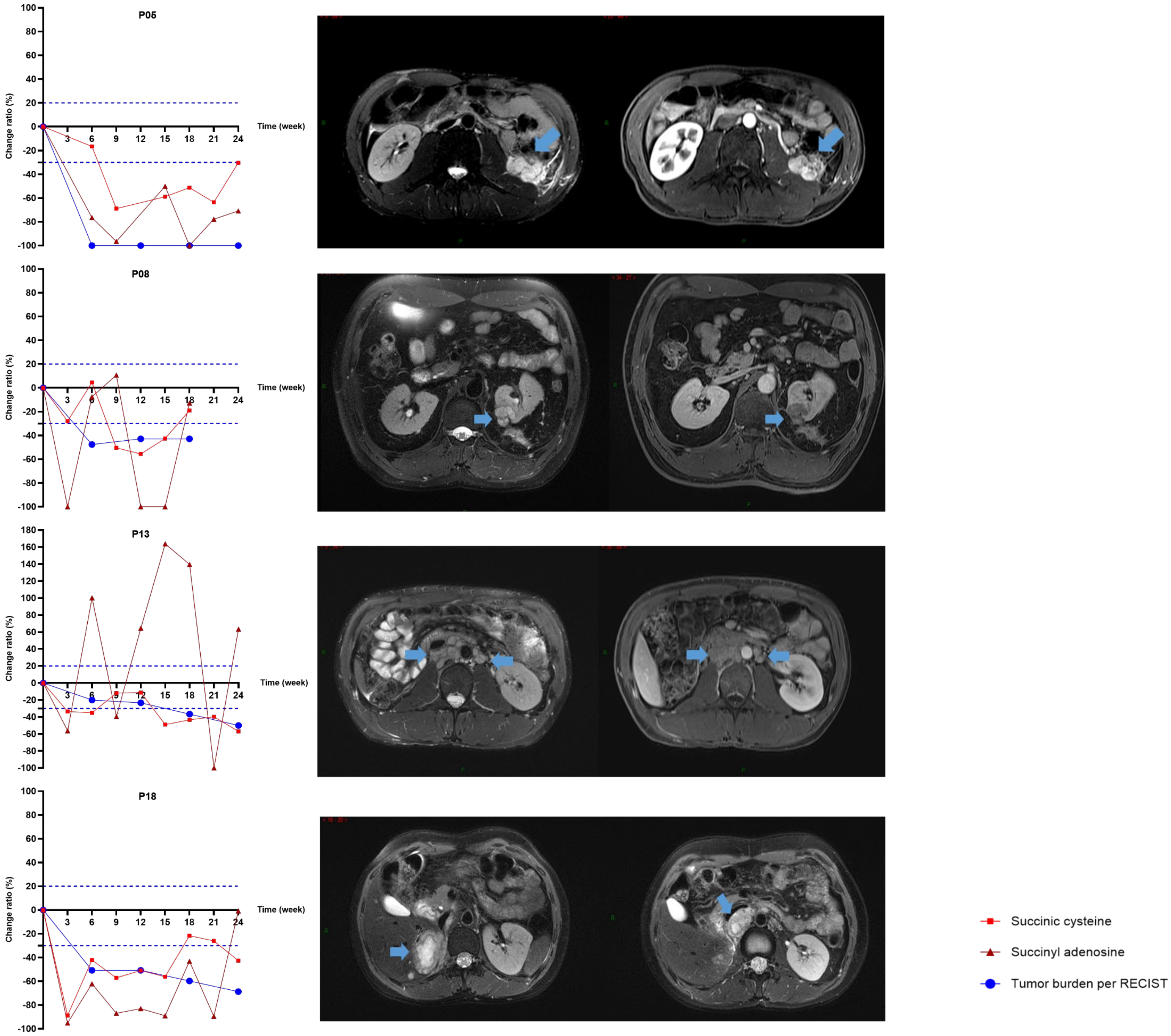


**Supplementary Fig. S2.** Biomarker trajectories and corresponding baseline imaging in four patients with both baseline succinic-cysteine and succinyl-adenosine under the pre-specified thresholds. Four patients (P05, P08, P13 and P18) had low, not zero, biomarker levels. Their metabolite concentrations fell below the predefined thresholds (succinic-cysteine 32 ng/mL, succinyl-adenosine 12 ng/mL), which could lead to unreliable measurements. At such low concentrations, multiple factors compromise data quality, including low signal-to-noise ratio; high proportional technical variability; marked instability in serial measurements among which biomarker trajectories oscillate widely without any correlation with tumor burden changes on imaging. Therefore, excluding these patients was a methodological necessity to avoid analyzing unreliable data, not a form of selection bias. When we go back to the imaging, these 4 patients predominantly had low tumor burden and localized lymph node recurrence. In these patients, the absolute concentration of circulating metabolites likely falls below the current assay's limit of quantification. We explicitly acknowledge that, for patients with levels below the threshold, the current assay is not reliable and should not be used for clinical monitoring.

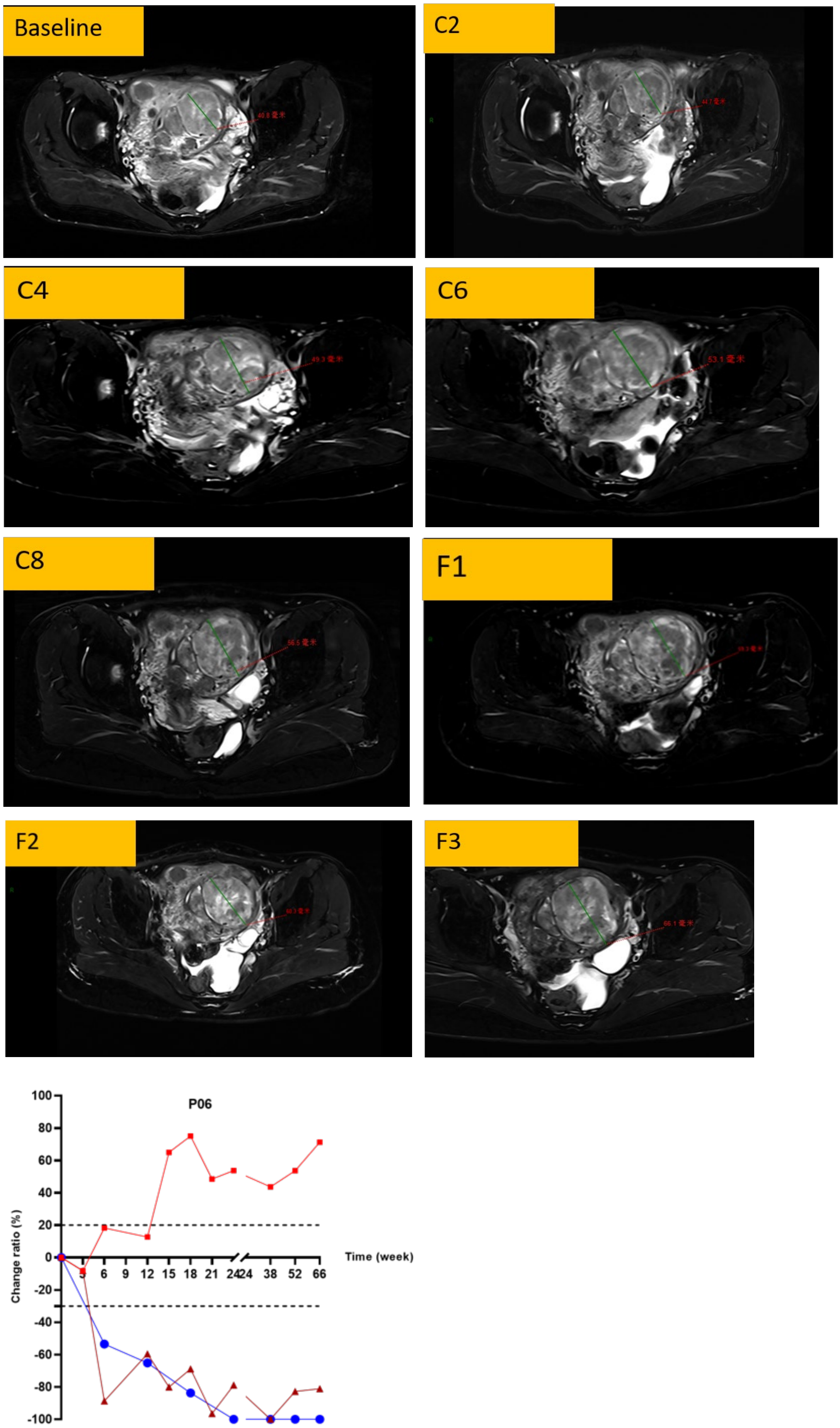
Baseline
C2
C4
C6
C8
F1
F2
F3
P06
Change ratio (%)
100
80
60
40
20
0
-20
-40
-60
-80
-100
3
6
9
12
15
18
21
24
24
38
52
66
Time (week)

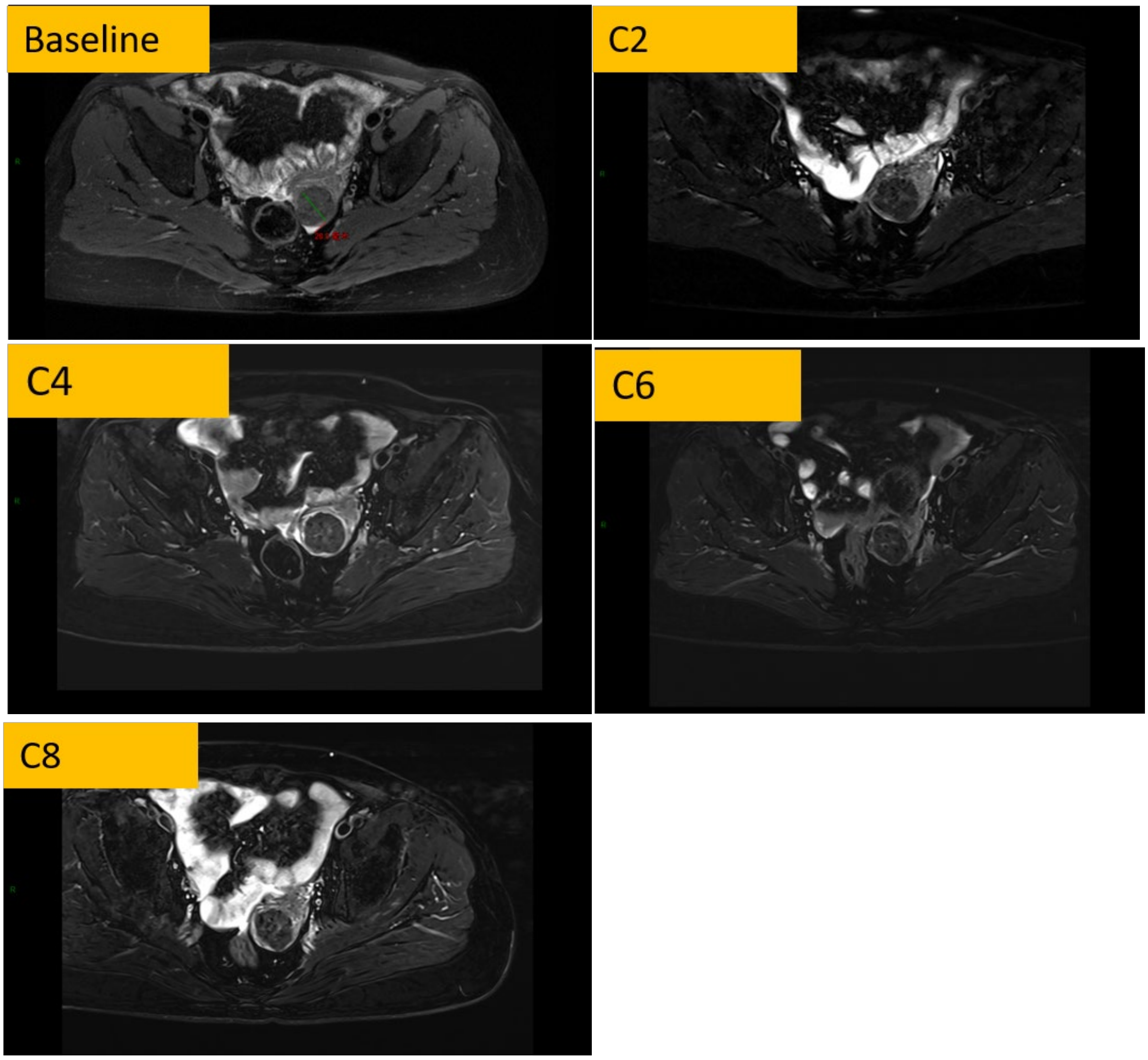


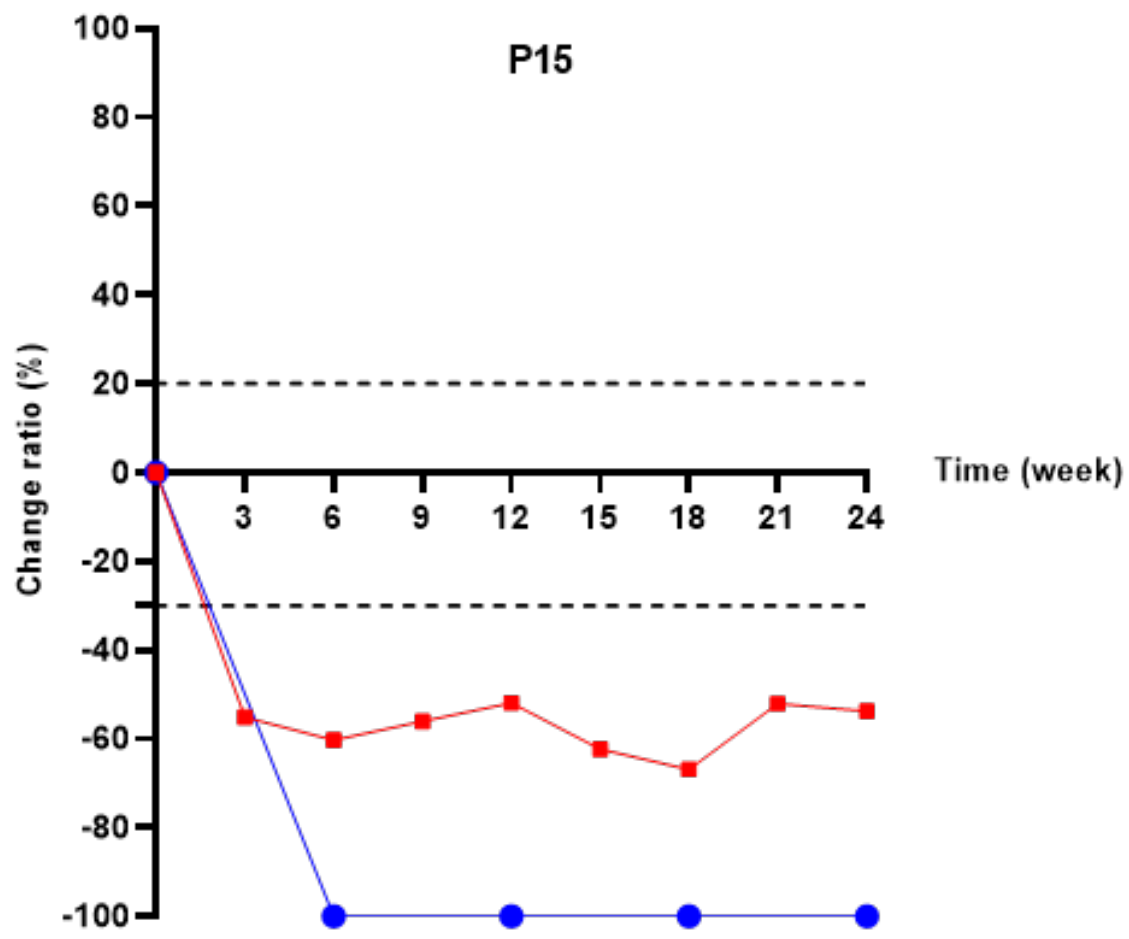


**Supplementary Fig. S3.** Representative imaging for female patients with treatment-naïve uterine leiomyomas. In Patient P06 with multiple untreated leiomyoma, succinic cysteine increased progressively and disproportionately to the responding metastatic disease, while coinciding with radiographic slow enlargement of the leiomyoma. Succinyl-adenosine however, remained correlated with tumor burden. In Patient P15 with stable leiomyoma, succinic cysteine decreased with treatment but not as steeply as tumor burden, possibly reflecting a persistent low-level signal from the

leiomyoma. Succinyl-adenosine was undetectable at baseline, possibly due to lower tumor burden or different biochemistry mechanism.

# Supplementary Note 2:

Title: Tislelizumab plus lenvatinib as the first-line therapy in patients with advanced fumarate hydratase-deficient renal cell carcinoma: a single-arm, open-label, prospective study

Center: Renji Hospital, Shanghai Jiao Tong University School of Medicine

Principal Investigator: Jin Zhang, M.D., Ph.D.

Sub-Investigator: Wen Kong, M.D.

Yunze Xu, M.D., Ph.D.

Guiqin Liu, M.D.

Guangyu Wu, M.D.

Zaoyu Wang, M.D.

Dengfeng Cao, M.D., Ph.D.

Feng Jiao, M.D.

Jiong Hu, M.D.

Liang Zheng, Ph.D.

Wei Xue, M.D.

Statistician: Wenwen Lv, Ph.D.

# TABLE OF CONTENTS


1. OBJECTIVES ........ 4

1.1 Primary Objectives ........ 4

1.2 Secondary Objectives ........ 4

1.3 Exploratory Objectives ........ 4

2. BACKGROUND ........ 4

2.1 Epidemiological and Clinical Features of FH-RCC ........ 4

2.2 Progress in the Treatment of Advanced FH-RCC ........ 5

2.3 Metabolic Biomarkers of FH-RCC ........ 6

2.4 Rational of the Study Design ........ 7

2.5 Tislelizumab ........ 10

2.6 Lenvatinib ........ 11

3 PATIENT SELECTION ........ 12

3.1 Inclusion Criteria ........ 12

3.2 Exclusion Criteria ........ 14

3.3 Inclusion of Women and Minorities ........ 15

4 STUDY PLAN ........ 15

4.1 Tislelizumab ........ 15

4.2 Lenvatinib ........ 16

4.3 Duration of Therapy ........ 16

4.4 Concomitant Medications ........ 16

4.5 Contingency for Surgical or Radiation Intervention ........ 18

4.6 Withdrawal or Termination ........ 18

4.7 Criteria for Treatment Continuation beyond Disease Progression ........ 19

5 DOSE DELAY OR MODIFICATION ........ 19

5.1 Dose Modification of Tislelizumab ........ 20

5.2 Dose Modification of Lenvatinib ........ 22

6 CLINICAL AND LABORATORY EVULATIONS ........ 24

6.1 Study Calendar ........ 24

6.2 Pretreatment Evaluation ........ 28

6.3 Evaluation during Treatment 29
6.4 Evaluation at the End of Treatment 30
6.5 Evaluation during Follow-up 30
7 ADVERSE EVENTS AND REPORTING REQUIREMENTS 31
7.1 Definition 31
7.2 AE/SAE Records and Reports 32
7.3 Abnormal Laboratory Results 33
7.4 Disease Progression 33
7.5 Reporting of Death Events 33
7.6 Reporting of Pregnancy Events 34
7.7 Drug Overdose 34
8 MEASUREMENTS OF EFFECT 34
8.1 Definitions 34
8.2 Guidelines for Evaluation 36
8.3 Imaging Scan Protocol 36
8.4 Biomarker Evaluation 37
9 DATA AND PROTOCOL MANAGEMENT 37
10 STATISTICAL CONSIDERATIONS 38
10.1 Sample Size 38
10.2 Analysis Plan 38

# 1. OBJECTIVES

## 1.1 Primary Objectives

To evaluate the efficacy of tislelizumab plus lenvatinib as the first-line treatment in patients with unresectable, or recurrent, or metastatic fumarate hydratase-deficient renal cell carcinoma (FH-RCC), depicted by objective response rate (ORR) by investigators according to the response evaluation criteria for solid tumors (RECIST)1.1.

## 1.2 Secondary Objectives

(1) To evaluate the disease control rate (DCR), clinical benefit rate, duration of response (DOR), progression-free survival (PFS), and overall survival (OS) evaluated by investigators in FH-RCC patients treated with tislelizumab plus lenvatinib in the first-line setting.
(2) To evaluate the safety of tislelizumab plus lenvatinib as the first-line treatment in patients with advanced FH-RCC.

## 1.3 Exploratory Objectives

(1) To explore the correlation between tumor burden and plasma or urine succinate-modified metabolites (succinyl-adenosine and succinic-cysteine) in patients with advanced FH-RCC treated with tislelizumab plus lenvatinib.
(2) To explore the correlation between tumor microenvironment characteristics (including PD-L1 expression and lymphocyte infiltration, etc.) and the efficacy of tislelizumab plus lenvatinib in patients with advanced FH-RCC

# 2. BACKGROUND

## 2.1 Epidemiological and Clinical Features of FH-RCC

Renal cell carcinoma (RCC) accounts for 90% of all renal malignancies, including clear cell carcinoma, papillary renal cell carcinoma, chromophobe renal cell carcinoma, collecting duct carcinoma and other pathological subtypes, each of which has unique clinical, pathological and prognostic characteristics. Hereditary leiomyomatosis and renal cell carcinoma (HLRCC) became an independent pathological subtype, in the 2016 edition of the World Health Organization (WHO)/International Association of Urology Pathology (ISUP) classification of renal malignancies[1]. HLRCC is a rare autosomal dominant tumor syndrome, with an incidence of about 1/200,000. It is caused by germline mutation of fumarate hydratase (FH) gene. Seventy percents of HLRCC patients have skin leiomyoma, over 80% of female HLRCC patients have uterine leiomyoma, and about 15% ~ 30% develop highly invasive renal cancer[2,3]. In recent years, it has been reported that a small number of RCC patients carried somatic biallelic *FH* mutations without family history of HLRCC or clinical manifestations of leiomyomatosis. The clinicopathological features of this type of RCC were almost the same as HLRCC-related RCC. Therefore, in the updated 2022 WHO/ISUP renal tumor pathological classification revision, "fumarate hydratase-deficient renal cell carcinoma (FH-RCC)" has been used to better define this type of tumor, including both "HLRCC-related RCC" caused by *FH* germline mutation and the other part of tumors

[4]with somatic *FH* biallelic mutations[5].

FH-RCC is a rare type of tumor. In the case cohorts reported in the literature to date, the number of individual cases has consistently been below 40. A UK multicenter retrospective study documented 185 patients from 69 HLRCC families, among which 23 were diagnosed with FH-RCC[6]. A nationwide study in France identified 114 HLRCC families, including 37 cases of FH-RCC[7]. A PubMed literature review summarized 672 reported cases of HLRCC syndrome as of November 2019, among which 189 involved renal cancer[8]. Local recurrence or distant metastasis is found in 50%-80% of patients at the time of initial diagnosis, with a median survival time of 18-21 months[6-9]. After evaluating 77 cases of FH-RCC from 15 centers across China, we found that 92.2 % had recurrence or metastasis and 30% died within a median follow-up of 26 months[10]. The survival data from the real world showed poor prognosis of FH-RCC and indicated clinical urgency to explore optimal treatment for patients with advanced FH-RCC.

**2.2 Progress in the Treatment of Advanced FH-RCC**

Due to the low incidence, no randomized controlled trial has been carried out for advanced FH-RCC. The only one prospective phase 2 trial to date assessed the efficacy and safety of bevacizumab plus erlotinib for advanced HLRCC-related RCC and sporadic papillary RCC[11]. Enrolled patients received no more than two lines of systemic treatments targeting VEGFR. The primary endpoint of the study was ORR, and the secondary endpoints included PFS and DOR. ORR of HLRCC cohort was 64% (27/42), and the median PFS was 21.1 months. In terms of safety, most treatment-related adverse events (AEs) were grade 1 or 2, and the most common AE were acne-like rash (92%), diarrhea (77%), proteinuria (71%) and dry skin (61%). Forty-seven percents of patients experienced ≥Grade 3 treatment-related AEs, including hypertension (34%) and proteinuria (13%). This was the only prospective study in HLRCC to date, and the results seemed to show that bevacizumab plus erlotinib had good anti-tumor activity in patients with advanced papillary RCC, especially in patients with advanced HLRCC-related RCC characterized by FH deficiency. Although this regimen has been recommended by the NCCN guidelines, it was not approved by the FDA, due to multiple confounding factors. The result of our retrospective real world study also suggested that bevacizumab plus erlotinib appeared to be less effective than expected[10].

Trials evaluating TKIs targeting VEGF/VEGFR or mTOR, or immune checkpoint inhibitors (ICI) for FH-RCC were of small size, and the results were controversial. Retrospective analysis of 32 cases of FH-RCC from MSKCC showed that mTOR/VEGF-TKI had an ORR of 44%, which was better than VEGF-TKI, mTOR or ICI monotherapy[12]. Another retrospective study from West China Hospital showed that the DCR of patients receiving ICI/TKI combination or ICI monotherapy in the first-line treatment was 100%, and that of patients receiving TKI alone was 58.3%. ICI-based first-line therapy (ICI/TKI combination or ICI monotherapy) achieved a median PFS of 13.3 months, suggesting that patients with advanced FH-RCC benefited more from ICI-based therapy[13]. Our multi-center real-world retrospective study also showed that ICI/TKI combination therapy as the first-line therapy had an ORR of 27% and a DCR of 85%. Median PFS was 18.0 months, and median OS was not reached. However, bevacizumab/erlotinib combination therapy led to an ORR of 25% and a median PFS of 10.0 months; VEGF-TKI single agent led to an ORR of 10% and a median PFS of 12.0 months. Our findings suggest that patients with advanced FH-deficient RCC appear to

benefit from ICI and TKI combination therapy[10].

**2.3 Metabolic Biomarkers of FH-RCC**

Diagnosis of FH-RCC is difficult. The imaging characteristics of FH-RCC are diverse[14,15]. Furthermore, pathological diagnosis of FH-RCC was also non-specific, while a number of cases were diagnosed as collecting duct carcinoma, medullary carcinoma or even clear cell carcinoma[9,16,17]. Combined application of antibodies against fumarate hydratase (FH) and 2-succinate cysteine (2-SC) in immunohistochemical staining can improve the accuracy of diagnosis, in which tumor cells of typical FH-RCC could show negative FH and strong positive 2-SC expression[7,18,19]. However, FH antibody has been reported to show a certain probability of false positivity, and 2-SC antibody has not yet been commercialized.

Another major challenge lies in the difficulty of monitoring and evaluation. Since a considerable proportion of FH-deficient renal carcinoma patients are diagnosed at an advanced stage, repeated imaging examinations such as contrast-enhanced CT or MRI are required to assess treatment response and monitor disease progression during systemic therapy. For some patients who have undergone surgical resection, frequent imaging is also necessary for early detection of tumor recurrence or metastasis. Given that FH-RCC patients typically require lifelong follow-up, this imposes cumulative radiation exposure, financial burdens, and various practical inconveniences. Therefore, the development of novel, simple, and non-invasive monitoring methods is of significant importance for these patients and their family members.

Liquid metabolic biomarkers in blood or urine represent a promising direction for non-invasive diagnosis. To date, neither FH-RCC nor the broader category of renal cell carcinoma has clinically available liquid tumor marker, which is equivalent to prostate-specific antigen (PSA) in prostate cancer, to assist in diagnosis and disease monitoring. However, the detection of metabolic biomarkers in body fluids has already been successfully applied in other cancers. For example, isocitrate dehydrogenase 1/2 (IDH1/2) gene mutations occur in approximately 15%–20% of acute myeloid leukemia (AML) cases. The detection of plasma 2-hydroxyglutarate (2-HG) levels can predict the presence and mutational burden of IDH1/2 mutations, with specificity and sensitivity reaching 99% and 92%, respectively, in AML diagnosis [23-25]. After induction therapy, 2-HG levels showed a strong positive correlation with minimal residual disease markers (such as WT-1 and NPM1), indicating its utility in monitoring disease progression[20-22]. The successful clinical application of 2-HG provides a model for developing non-invasive diagnostic and evaluation methods for FH-deficient renal carcinoma, which similarly involves a key enzyme deficiency in the tricarboxylic acid (TCA) cycle.

Our center's hereditary renal cancer research team has long focused on FH-RCC and has accumulated extensive clinical cases and research resources through sustained clinical work. To date, we have collected over 90 cases of FH-RCC, making our center one of the largest single-center repositories worldwide for this disease. Building on this foundation, we have successfully established primary cell lines, cell-derived xenograft (CDX) models, and patient-derived xenograft (PDX) models of FH-RCC. The laboratory component of our team has been consistently engaged in investigating the metabolic reprogramming mechanisms of this tumor type. In previous work, we

conducted a series of metabolomic studies using FH-deficient cell models, CDX models, tumor tissues, and patient plasma samples. These investigations revealed significantly elevated levels of succinate-modified metabolites, specifically succinyl-adenosine and succinic-cysteine, in the plasma of patients or tumor-bearing animal models. Following tumor resection or effective drug therapy, the levels of these two metabolic markers decreased, whereas they rose again upon tumor recurrence, systemic treatment resistance, or disease progression. Based on these findings, we hypothesize that succinate-modified metabolic markers, represented by succinyl-adenosine and succinic-cysteine, have the potential to serve as specific biomarkers for the diagnosis and monitoring of FH-RCC[23]. This discovery may fill a critical gap in the availability of tumor markers and non-invasive diagnostic techniques for FH-RCC.

Based on our preclinical work, this study will assess correlation between the tumor burden and dynamic changes of metabolic biomarkers, such as blood and urine succinate-modified metabolites (succinyl-adenosine and succinic-cysteine) along the whole treatment process as an exploratory endpoint.

## 2.4 Rationale of the Study Design

### 2.4.1 Pathogenesis of FH-RCC and the rationale for ICI/TKI combination therapy

Papillary renal cell carcinoma (PRCC), which constitutes 15%-20% of all renal cell carcinomas, represents a heterogeneous group. A previous multi-omics study of 161 papillary renal cell carcinomas revealed that type 2 PRCC is significantly associated with CDKN2A silencing, SETD2 mutations, TFE3 fusion genes, and dysregulation of the NRF2-mediated oxidative stress response pathway. Within type 2 PRCC, a subgroup characterized prominently by CpG island hypermethylation (CpG island methylator phenotype, CIMP) exhibited exceptionally high aggressiveness and distinct molecular and metabolic features: 56% of these tumors harbor germline or somatic mutations in the FH gene, accompanied by significant upregulation of cell cycle and hypoxia response pathways and prominent metabolic reprogramming. The CIMP subgroup represents the poorest prognostic category among papillary renal cell carcinomas[24], and research indicates that the majority of the CIMP subgroup essentially corresponds to FH-RCC.

Fumarate hydratase (FH) is a key enzyme in the tricarboxylic acid (TCA) cycle, reversibly catalyzing the conversion of fumarate to malate. Deficiency in FH leads to disruption of the intracellular TCA cycle, resulting in a classic "Warburg effect" characterized by impaired oxidative phosphorylation and other aerobic metabolic processes, alongside enhanced anaerobic metabolism such as glycolysis, and accumulation of fumarate. Studies on the FH-deficient cell line UOK262, derived from HLRCC-associated renal cancer, revealed that glucose-derived carbon atoms rarely entered the TCA cycle. Instead, glutamine-derived carbon accumulated in TCA cycle intermediates, including fumarate. This reflected an adaptive response known as "reductive carboxylation" adopted by FH-deficient cells to bypass the blocked TCA cycle, a process dependent on the pentose phosphate pathway for NADPH supply[25,26]. Furthermore, FH-deficient cells reversed the action of arginine-succinate lyase, catalyzing the reaction between fumarate and arginine to produce large amounts of arginine-succinate, thereby creating a metabolic dependency on arginine[27]. The accumulated fumarate in cells covalently binded to glutathione (GSH) via its α,β-unsaturated double bond. This reaction depleted the cell's most critical antioxidant, leading to increased reactive oxygen

species, generating substantial succinic-GSH, which promoted cellular senescence and, under specific conditions, facilitated tumor formation[28]. The electrophilic reaction between fumarate and cysteine thiol groups on intracellular proteins leads to the widespread formation of 2-succinocysteine (2-SC)[29]. On one hand, this reaction provides the theoretical basis for using 2-SC immunohistochemical staining to identify FH-RCC. On the other hand, this succination modification impairs protein function. The activity of dioxygenases—such as proline hydroxylase (PHD), the Jumonji domain family of histone lysine demethylases (JMJ-KDM), and the TET family of 5-methylcytosine hydroxylases (TET)—is directly inhibited. This inhibition prevents the hydroxylation of HIF1/2α, thereby blocking its ubiquitin-mediated degradation. Consequently, histones and DNA maintain a broadly hypermethylated state. This mechanism partly explains the link between FH gene mutations in the PRCC CIMP subgroup and genomic hypermethylation, upregulation of hypoxia pathways, and the observed "Warburg effect"[30-33].

A multi-omics study of 25 FH-RCC cases by Professor Hao Zeng's team at West China Hospital of Sichuan University revealed that the FH-RCC genome exhibited a hypermethylated phenotype. Among nearly 200,000 differentially methylated CpG islands compared to adjacent normal tissue, 66% showed elevated methylation levels. Integrated analysis with the TCGA-KIRP cohort indicated that most FH-RCC cases displayed a CpG island methylator phenotype (CIMP), and cases with lower methylation levels appeared to have a lower likelihood of recurrence and metastasis. Furthermore, the tumor microenvironment showed increased infiltration of $CD4^+$ T cells, $CD8^+$ T cells, activated NK cells, and macrophages, along with upregulated PD-L1 expression[13]. In summary, the accumulation of HIF within FH-deficient RCC tumor cells, combined with an immune-infiltrated microenvironment, provides a strong theoretical rationale for potential benefit from immune checkpoint inhibitor (ICI) / tyrosine kinase inhibitor (TKI) combination therapy.

2.4.2 Lenvatinib plus PD-1 antibody in advanced RCC: results of clinical trials

In April 2021, The New England Journal of Medicine published the results of the Phase 3 CLEAR trial, which investigated lenvatinib plus either pembrolizumab or everolimus versus sunitinib as first-line treatment for advanced clear cell renal cell carcinoma (ccRCC). This randomized controlled study enrolled 1,069 previously untreated patients with advanced ccRCC, who were assigned to one of three regimens: lenvatinib (20 mg orally once daily) plus pembrolizumab (200 mg intravenously every 3 weeks; n=355); lenvatinib (18 mg orally once daily) plus everolimus (5 mg orally once daily; n=357); or sunitinib (50 mg orally once daily on a 4-weeks-on/2-weeks-off schedule; n=357). The primary endpoint was PFS, with OS and safety as key secondary endpoints. Compared to sunitinib (median PFS 9.2 months), lenvatinib plus pembrolizumab significantly extended median PFS to 23.9 months (HR 0.39, 95% CI 0.32–0.49, p<0.001). Lenvatinib plus everolimus also showed superior PFS over sunitinib, with a median of 14.7 months (HR 0.65, 95% CI 0.53–0.80, p<0.001). Although median OS was not reached in any group, the 2-year OS rates were 79.2% for lenvatinib-pembrolizumab, 66.1% for lenvatinib-everolimus, and 70.4% for sunitinib, with lenvatinib-pembrolizumab demonstrating a significant OS benefit. In terms of clinical response, the ORR were 71.0%, 53.5%, and 36.1% for the three groups, respectively. Notably, the lenvatinib-pembrolizumab arm achieved a complete remission (CR) rate of 16.1%, and the lenvatinib-everolimus arm a CR of 9.8%, both significantly higher than that in the sunitinib group. Regarding safety, the incidence of grade ⩾3 AEs was 82.4%, 83.1%, and 71.8% in the three

groups, respectively. In the lenvatinib-pembrolizumab group, 37.2% of patients discontinued at least one study drug due to AEs, 68.8% required lenvatinib dose reduction, and 78.4% experienced treatment interruption. Common AEs included hypertension, diarrhea, and elevated lipase[34]. The lenvatinib plus pembrolizumab regimen in the CLEAR study achieved the highest ORR and CR rates reported to date in first-line systemic therapy for advanced ccRCC. Its safety profile was generally consistent with that of other combination therapies, leading to its rapid approval by the FDA and inclusion in NCCN guidelines as a standard first-line treatment option.

At the 2022 ESMO Annual Meeting, interim results (⩾24 weeks) from the Phase II KEYNOTE-B61 study investigating first-line lenvatinib plus pembrolizumab in advanced non-clear cell renal cell carcinoma (nccRCC) were presented. As of the data cutoff, 82 patients with advanced nccRCC had been enrolled, with a histologic distribution of 62.2% papillary, 18.3% chromophobe, and 6.1% translocation RCC. After a median follow-up of 8.2 months, an ORR of 47.6% was observed, with an ORR of 52.9% in patients with advanced PRCC. The 6-month PFS rate was 72.3%, and the 6-month OS rate was 87.8%. The incidence of Grade ⩾3 AEs was 35%. These data suggest that first-line treatment with lenvatinib combined with a PD-1 inhibitor may offer favorable efficacy and safety in advanced nccRCC.

Given that FH-RCC represents a highly aggressive pathological subtype within nccRCC, and based on the previously elucidated understanding of its pathogenesis and tumor microenvironment characteristics, we anticipate that the therapeutic regimen combining a PD-1 inhibitor with lenvatinib may provide clinical benefit for patients with advanced FH-RCC.

2.4.3 Lenvatinib plus tislelizumab in patients with advanced hepatocellular carcinoma: results of clinical trials

The combination regimen of tislelizumab plus lenvatinib has been preliminarily investigated for efficacy and safety in advanced hepatocellular carcinoma (HCC). A prospective, single-arm, multicenter Phase II clinical study reported at the ESMO Immuno-Oncology Congress 2022 enrolled 64 previously untreated patients with unresectable, advanced HCC. Patients received tislelizumab (200 mg every 3 weeks) plus lenvatinib (12 mg or 8 mg once daily). The primary endpoint was ORR, with secondary endpoints including safety, duration of response (DOR), disease control rate (DCR), and PFS.

As of the data cutoff, with a median follow-up of 12.5 months, 62 patients were evaluable for efficacy. The ORR as assessed by an Independent Review Committee (IRC) was 38.7% (24/62), while investigator-assessed ORR was 41.9% (26/62). The DCR was 90.3% (56/62) and 85.5% (53/62) per IRC and investigator assessment, respectively. A reduction in target lesions was observed in 74.2% to 80.6% of subjects. The median PFS was 9.6 months per IRC and 8.5 months per investigator assessment.

Regarding safety, the incidence of Grade ⩾3 treatment-related AEs was 28.1% (18/64). Treatment-related serious AEs occurred in 9.4% (6/64) of patients, and Grade ⩾3 immune-related AEs were reported in 12.5% (8/64). Systemic corticosteroid treatment was required for 6.3% (4/64) of subjects. The most common treatment-related AEs (incidence >10%) included proteinuria, hypertension, and

hypothyroidism.

This study demonstrates that the combination of tislelizumab and lenvatinib has a favorable and manageable safety and tolerability profile.

2.4.4 Rationale for patient enrollment

As previously mentioned, there is no standard-of-care established for patients with advanced FH-RCC. AVATAR study (https://clinicaltrials.gov/ct2/show/NCT01130519) was the only one prospective phase 2 trial to date, which showed that bevacizumab (10 mg/kg Q2W) plus erlotinib (150 mg QD) achieved an ORR of 64% and a median PFS of 21.1 months in patients with advanced HLRCC. The most common AEs included acneiform rash (92%), diarrhea (77%), proteinuria (71%), and dry skin (61%), and Grade⩾3 treatment-related AEs occurred in 47% of patients, including hypertension (34%) and proteinuria (13%).These results appear to suggest that bevacizumab combined with erlotinib has promising antitumor activity in advanced FH-RCC, particularly in HLRCC patients[11]. This regimen is recommended by the NCCN guidelines, however, has not yet received FDA approval or CSCO recommendation, as the study had been conducted over a decade and probably influenced by numerous confounding factors. Meanwhile, retrospective data from our center indicated that the therapeutic efficacy of this regimen seemed to fall short of expectations.
Since the treatment of advanced FH-RCC does not have a unified plan supported by high-level evidence-based medical evidence, retrospective studies from our center suggest that ICI/TKI may bring survival benefits to patients with advanced FH-RCC[10]. We believe that it is reasonable to carry out clinical research on the combined treatment of lenvatinib and tislelizumab in patients with advanced FH-RCC.

**2.5 Tislelizumab**

Tislelizumab is a humanized monoclonal antibody against PD-1. It has been approved for relapsed or refractory classical Hodgkin's lymphoma, locally advanced or metastatic urothelial cancer, locally advanced or metastatic squamous non-small cell lung cancer and hepatocellular carcinoma. The $EC_{50}$ of tislelizumab binding to human recombinant PD-1 is 0.12 nM, Kd values are $1.45 \times 10^{-10}$ (PD-1 low density) and $1.10 \times 10^{-11}$ (PD-1 high density). The $IC_{50}$ of inhibiting PD-1 binding to PD-L1 is about 0.5 nM, and the $IC_{50}$ of inhibiting PD-1 binding to PD-L2 is about 0.4-0.6 nM.

Pharmacokinetic data for tislelizumab derived from population pharmacokinetic analyses in 798 patients with advanced malignancies in three clinical studies (BGB-A317-001, BGB-A317-102, and BGB-A317-203) and from non-compartmental pharmacokinetic analyses in 112 patients with advanced malignancies. These patients received 0.5, 2, 5, or 10 mg/kg every two weeks, or 2 or 5 mg/kg every three weeks, or 200 mg every three weeks. After a single intravenous infusion of this product, the drug exposure (Cmax and AUC0-14d) increased proportionally with dose in the dose range of 0.5 mg/kg to 10 mg/kg. After a single intravenous infusion of 200 mg of this product in Chinese patients with advanced solid tumors (n = 19), the average distribution volume (SD) of this product was 4.41±1.04 L. Based on population pharmacokinetic analysis, the steady-state volume of distribution (Vss) was 5.247 L. The average clearance (SD) of this product was 0.247±0.0918 L/day and the average half-life (SD) was 13.3±2.95 days. Based on population pharmacokinetic analysis, the clearance rate of this product is 0.171 L/day, the interindividual variation is 31.9, and

the terminal half-life is about 26 days.

Safety information for tislelizumab derived from four single-agent clinical studies (BGB-A317-001 [N = 451], BGB-A317-102 [N = 300], BGB-A317-203 [N = 70], and BGB-A317-204 [N = 113]) involving 934 patients. Tumor types included urothelial carcinoma (N = 152), non-small cell lung cancer (N = 105), esophageal cancer (N = 81), gastric cancer (N = 78), classical Hodgkin lymphoma (N = 70), hepatocellular carcinoma (N = 69), colorectal cancer (N = 54), ovarian cancer (N = 51), renal cell carcinoma (N = 37), melanoma (N = 36), breast cancer (N = 32), head and neck squamous cell carcinoma (N = 29), nasopharyngeal carcinoma (N = 27), cholangiocarcinoma (N = 18), pancreatic cancer (N = 10), small cell neuroendocrine carcinoma (N = 10), sarcoma (N = 10), mesothelioma (N = 9), cervical cancer (N = 7), and other types of tumors (N = 49). In the above study, 496 patients received the treatment of 200 mg once every 3 weeks, 355 patients received the treatment of 5 mg/kg once every 3 weeks, 26 patients respectively received the treatment of 2 mg/kg or 5 mg/kg once every 2 weeks, and 21 patients received the treatment of 2 mg/kg once every 3 weeks, seven patients were treated with 10 mg/kg every 2 weeks and 3 patients were treated with 0.5 mg/kg every 2 weeks. The median duration of administration was 16 weeks (range: 0.6-160.4 weeks), with 36.1 percent of patients receiving treatment for ⩾ 6 months and 20.6 percent receiving treatment for ⩾ 12 months.

The incidence of all grades of AEs in 934 patients treated with this product was 70.8. AEs with an incidence of ⩾ 10% included skin rash, fatigue, and elevated alanine aminotransferase. The incidence of ⩾ Grade 3 AEs was 21.8%, and AEs with incidence ⩾ 1% included increased gamma-glutamyl transferase, anemia, increased aspartate aminotransferase, increased alanine aminotransferase, pneumonia (non-infectious), severe skin reaction, and hypokalemia.

**2.6 Lenvatinib**

Lenvatinib is manufactured by ChaiTai Tianqing Pharmaceuticals and approved for patients with unresectable hepatocellular carcinoma who have not previously received systemic treatment.

It is a TKI inhibiting the kinase activity of vascular endothelial growth factor (VEGF) receptors VEGFR1(FLT1), VEGFR2(KDR), and VEGFR3(FLT4). The combination of lenvatinib and everolimus can inhibit the proliferation of human endothelial cells, angiogenesis and VEGF signaling pathway in vitro, and reduce the tumor volume of human renal cell carcinoma tumor-bearing mice in vivo, and the anti-angiogenesis and anti-tumor activity of the combination is greater than that of single drug use.

The pharmacokinetic parameters of lenvatinib have been studied in healthy adult subjects as well as in adult subjects with hepatic insufficiency, renal insufficiency and solid tumors.

After oral administration, lenvatinib is absorbed, and tmax is usually observed 1 to 4 hours after administration. Food does not affect the degree of absorption but can slow down the rate of absorption. The in vitro binding of lenvatinib to human plasma proteins was high and ranged from 98%-99% (0.3-30 μg/mL, mesylate). It binds mainly to albumin and lightly to α1-acid glycoprotein

and γ-globulin. In vitro, the lenvatinib blood-plasma concentration ratio ranged from 0.589 to 608(0.1-10 μg/mL, mesylate salt). In patients, the median volume of distribution for the first dose ranged from 50.5L to 92L, with substantial consistency in the 3.2mg to 32mg dose groups. Similar median steady-state volume of distribution was also largely consistent, ranging from 43.2L to 121L.

The efficacy of lenvatinib was evaluated in a randomized, open-label, international, multicenter clinical study (REFLECT; NCT01761266) in patients with previously untreated unresectable hepatocellular carcinoma (HCC). A total of 954 patients were randomized, of which 478 were assigned to lenvatinib and 476 to sorafenib.Lenvatinib was non-inferior to sorafenib in OS. Median OS was 13.6 months in the lenvatinib group and 12.3 months in the sorafenib group, with HR = 0.92 (95% CI 0.79-1.06).

The safety of lenvatinib was also evaluated in REFLECT study. Most patients (99%) in the lenvatinib group had ≥ 1 AE. The most common adverse reactions observed in patients treated with lenvatinib (≥ 20%) were as follows, in descending order of frequency: hypertension (45%), fatigue (44%), diarrhea (39%), decreased appetite (34%), weight loss (31%), arthralgia/myalgia (31%), abdominal pain (30%), palmoplantar redness syndrome (27%), proteinuria (26%), bleeding events (25%), dyspnea (24%), hypothyroidism (21%) and nausea (20%).

Grade ≥ 3 AEs occurred in 75% of patients in the lenvatinib group. The most common ≥ grade AEs observed in lenvatinib-treated patients (≥ 5%) were hypertension (24%), weight loss (8%), fatigue (7%), elevated blood bilirubin (7%), proteinuria (6%), decreased platelet count (5%), hepatic encephalopathy (5%), elevated gamma-glutamyl transferase (5%), bleeding events (5%) and aspartate aminotransferase elevation (5%).

The most common SAEs (≥ 2%) in lenvatinib-treated patients were bleeding events (5%), hepatic encephalopathy (5%), liver failure (3%), ascites (3%), and decreased appetite (2%).

AEs led to dose reductions or discontinuations in 62% of patients receiving lenvatinib. The most common AEs (≥ 5%) leading to dose reduction or discontinuation in the lenvatinib group were fatigue (10%), decreased appetite (8%), diarrhea (8%), proteinuria (7%), hypertension (6%), and palmoplantar swelling syndrome (5%).

In the lenvatinib-treated group, 20% of patients discontinued treatment due to AEs. The most common AEs (≥ 1%) leading to withdrawal of lenvatinib were fatigue (2%), bleeding events (2%), hepatic encephalopathy (2%), hyperbilirubinemia (1%), and liver failure (1%).

## 3 PATIENT SELECTION

### 3.1 Inclusion Criteria

(1) Fully comprehends and voluntarily signs the informed consent form, and agrees to undergo treatment, examinations, and follow-up assessments in accordance with the protocol.
(2) Age≥18, <80 years old, male or female.

(3) ECOG score ≤2.
(4) Histologically or molecularly confirmed unresectable, locally advanced or metastatic FH-RCC without prior systemic therapy, prior cytokine therapy allowed.
(5) At least one measurable lesion as per RECIST 1.1. Lesions with a history of radiotherapy can be selected as the targets if progresses again.
(6) Agree to provide blood and urine samples, and previously archived or fresh tumor tissue samples at the time points specified in the protocol, for participants in the exploratory investigation.
(7) Adequate function reserve of vital organs (Table 1):

| System | Inclusion criteria |
|---|---|
| **Hematopoietic function** (no blood transfusion or blood products within 2 weeks before enrollment, no history of hematopoietic stimulating factor or other drugs used to treat hematological abnormality): | |
| Neutrophil count | ≥ $1.5\times10^{9}$ /L |
| Hemoglobin | ≥ 9g/dL |
| Platelet count | ≥ $100\times10^{9}$ /L |
| Prothrombin time (PT) or internationally standardized ratio (INR) | ≤ 1.5×ULN |
| Partial thromboplastin time (APTT). | ≤ 1.5×ULN |
| **Liver function** | |
| Total bilirubin | ≤ 1.5 × ULN; Or total bilirubin > 1.5 × ULN but direct bilirubin within normal range |
| Alanine aminotransferase, aspartate aminotransferase (ALT, AST) | ≤ 2.5×ULN |
| ALT, AST in patients with liver metastases | ≤ 5×ULN |
| **Renal function** | |
| Serum creatinine (SCr) | ≤1.5×ULN |
| **Endocrine function** | |
| Thyroid-stimulating hormone (TSH) | Normal; or abnormal TSH, but FT3 FT4 within normal range |
| **Cardiac function** | |
| Left ventricular ejection fraction (LVEF) | ≥50% |

ULN: Upper limit of normal
(8) Female subjects of childbearing potential must have a negative serum pregnancy test within 7 days prior to the first dose. Both female subjects of childbearing potential and male subjects with fertile partners must agree to use highly effective contraception during the study and for 180 days after the last dose of the study drug.

**3.2 Exclusion Criteria**

(1) Prior treatment with agents targeting VEGF, VEGFR, or mTOR, including but not limited to sunitinib, axitinib, pazopanib, sorafenib, cabozantinib, lenvatinib, bevacizumab, anlotinib, or everolimus.
(2) Prior treatment with antibody agents against anti-PD-1, PD-L1 or CTLA-4.
(3) Receipt of any other investigational product within 4 weeks prior to the first dose of the trial drug, except during an observational (non-interventional) clinical study or the follow-up phase of an interventional study.
(4) Any major surgery or recovery from surgery within 4 weeks before the first dose of investigation agents (as judged by the investigator).
(5) Use of Chinese medicine or Chinese patent medicine with anti-tumor indications within 2 weeks the first dose of investigation agents.
(6) Adrenocorticosteroids (>10mg prednisone daily or equivalent) or other immunosuppressive therapy within 2 weeks before the first dose of investigation agents; Inhalation agents of > 10mg prednisone daily without active autoimmune disease is allowed for enrollment.
(7) History of organ transplantation or long-term adrenocortical hormone or immunosuppressive therapy.
(8) Thyroid, adrenal or pituitary gland dysfunction controlled only by hormone replacement therapy, Type I diabetes, and psoriasis or vitiligo not requiring systemic treatment, etc., can be enrolled.
(9) Toxicity not resolved after previous antineoplastic therapy, i.e. not resolved to baseline, or NCI-CTCAE 5.0 specified Grade 0 to 1 (except alopecia), or the level specified in the inclusion/exclusion criteria. Subjects with irreversible toxicities that are not expected to be exacerbated by the study drug (e.g., hearing loss) may be enrolled in this study.
(10) Other malignancy that have progressed or need treatment within 5 years (excluding skin basal cell carcinoma, skin squamous cell carcinoma, superficial bladder cancer or cured carcinoma in situ, such as breast carcinoma in situ and prostate cancer; Subjects with localized low-risk prostate cancer (⩽ T2a, Gleason ⩽ 6, PSA<10ng/ml) who received radical treatment and have no PSA biochemical recurrence can be enrolled in this study).
(11) History of active central nervous system (CNS) metastases, or baseline imaging indicating CNS metastasis within 30 days prior to the first dose. Subjects with previous surgery or radiation therapy for brain or meningeal metastases with clinically stable disease for ⩾ 3 months and not requiring systemic hormone therapy (prednisone or other equivalent hormone at a dose of > 10mg/day) for > 4 weeks may be enrolled. Subjects with CNS metastases can be enrolled in the study if they are adequately treated and if their CNS symptoms have recovered to grade ⩽ 1 for at least 2 weeks prior to enrollment (except for residual signs or symptoms related to CNS treatment).
(12) Poorly controlled hypertension: SBP ⩾ 150mmHg and/or DBP ⩾ 90mmHg.
(13) Have any one of the following cardiovascular disease states within the past 6 months: myocardial infarction; unstable angina; endovascular angioplasty or coronary stent implantation; coronary/peripheral artery bypass grafting; NYHA cardiac function class 3-4; congestive heart failure; cerebrovascular accident including transient ischemic attack.
(14) Heart rate corrected QT interval (QTc)⩾ 480ms.
(15) History of active bleeding or other severe bleeding within 1 month.
(16) Deep vein thrombosis or pulmonary embolism within 6 months.
(17) Occurrence of arterial embolism within the last 12 months.

(18) Clinically significant gastrointestinal abnormalities, including: malabsorption, total gastrectomy, or any condition affecting the absorption of oral medications; active ulcers treated within the previous 6 months; active gastrointestinal bleeding (hematemesis, hematochezia, or melena) within the previous 3 months without endoscopic evidence of recovery; metastatic lesions in the gastrointestinal tract with suspected bleeding, inflammatory bowel disease, ulcerative colitis, gastrointestinal perforation or other gastrointestinal disorders that increase the risk of perforation.
(19) Previous history or current presence of (non-infectious) pneumonia/interstitial lung disease requiring corticosteroid therapy.
(20) Active infection requiring systemic treatment, human immunodeficiency virus (HIV) infection (known HIV antibody positive), active HBV infection (HBsAg positive, or HBcAb positive but HBsAg negative requiring further DNA quantification test and with the results not exceeding 2×ULN), active HCV infection (HCV RNA test result negative during screening period of previous HCV infection can be included).
(21) Live vaccines, including but not limited to mumps, rubella, measles, varicella/shingles, yellow fever, rabies, BCG and typhoid vaccine, excluding inactivated virus vaccine.
(22) History of severe drug allergy, including but not limited to antibody drugs and small molecule targeted drugs.
(23) Known history of mental illness or history of substance abuse.
(24) There are unhealed wounds.
(25) As per judgment of the investigator, the subject has a history or current evidence of any disease, treatment, or laboratory abnormality that may confuse the results of the trial, interfere with the subject's participation, or is not in the best interest of the subject participating in the trial.

### 3.3 Inclusion of Women and Minorities

Both men and women and members of all races and ethnic groups are eligible for this trial.

## 4 STUDY PLAN

### 4.1 Tislelizumab

Tislelizumab is administered intravenously 200mg every 3 weeks on the first day of each cycle, and the first intravenous infusion time should be over 60 minutes. If the first infusion is well tolerated, the time of the second infusion can be shortened to 30 minutes. If the 30-minute infusion is well tolerated, all subsequent infusions can be completed within 30 minutes. If an infusion reaction occurs in previous treatment, subsequent infusions should be maintained for over 60 minutes.

The dosing time window per cycle can be ± 3 days (C1D1 dosing time window is +3 days). All required examinations every cycle should be completed at least 72h before each administration (C1D1 can be completed within 7 days before administration) to assess tolerance, except imaging examinations.

The delay (not caused by AE) is generally allowed for no more than 3 days. If the delay exceeds 3 days, the next planned administration time is recalculated based on the actual administration date, and the subsequent administration time window is still ± 3 days.

Dose adjustment is not permitted. Delayed administration (caused by AE and other reasons) is allowed, and the scheduled administration is allowed to be delayed for up to 12 weeks, calculated from the last administration date, otherwise the treatment is terminated.

### 4.2 Lenvatinib

The starting dose of lenvatinib is 20mg QD by mouth and should be taken at a fixed time every day. The medication should continue until the criteria of discontinuation is met. In case of AE, the dose of lenvatinib can be gradually reduced to 16mg, 12mg, and 8mg QD. It is not recommended to reduce the dose of lenvatinib below 8mg QD. If there is still a ≥Grade 3 AE or intolerable Grade 2 AE during 8mg QD treatment, the treatment will be terminated.

### 4.3 Duration of Therapy

In the CLEAR study investigating first-line treatment of pembrolizumab plus lenvatinib in advanced clear cell renal cell carcinoma (ccRCC), the median time to response (mTTR) for the combination regimen was 1.94 months. In a phase Ib/II study of pembrolizumab plus lenvatinib for metastatic ccRCC previously treated with ≤2 lines of systemic therapies, nearly all responding patients achieved an objective response within 6 months. The primary endpoint of this study is objective response rate (ORR). Accordingly, enrolled patients receive the first 24 weeks (8 cycles) of combination therapy, during which imaging evaluation is taken every 6 weeks. After the initial 24-week treatment period, patients who demonstrate clear response and or benefit to therapy will be evaluated on a case-by-case basis and the investigators communicate with the subject according to the RECIST assessment results and safety tolerance to decide whether to continue the treatment. If participants remain in this regimen, imaging evaluation and safety follow-up will be taken every 3 months. Treatment will continue until disease progression, intolerable toxicity, or withdraw of consent.

### 4.4 Concomitant Medications

Concomitant medication refers to any additional treatments administered alongside the investigational agents, based on the investigator's judgment and in the interest of the subject's well-being. All concomitant medications, blood products, and non-pharmacological interventions (e.g., punctures) administered to the subject from the screening period through the safety follow-up duration must be rigorously documented in the Case Report Form (CRF) in full compliance with Good Clinical Practice (GCP) requirements.

4.4.1 Permitted concomitant medications

(1) Supportive care for disease-related symptoms.

(2) Topical, ocular, intra-articular, intranasal, and inhaled glucocorticoids; or ≤ 10mg/day prednisone or equivalent glucocorticoids allowed.

(3) Administration of corticosteroids at the dosage specified in the local prescribing information is permitted as premedication for the prevention of contrast agent allergies. Short-term use (≤3 weeks)

of corticosteroids is allowed for the treatment of non-autoimmune conditions (e.g., delayed-type hypersensitivity reactions caused by contact allergens).
(4) Hormone replacement therapy initiated prior to the first dose of the investigational product is permitted. For example, subjects receiving GnRH inhibitors may continue treatment if they have tolerated the therapy well for at least 3 months before enrollment. Subjects with a history of autoimmune-mediated hypothyroidism on a stable dose of thyroid replacement hormone are eligible for the study.
(5) Bisphosphonates or RANK-L inhibitors for bone metastases initiated prior to the first dose of the investigational product are allowed.
(6) Palliative local interventions (including palliative radiotherapy for symptomatic non-target isolated bone metastases, and drainage of symptomatic pleural effusion, pericardial effusion, or ascites) are permitted. It must be clearly documented that such interventions are not indicative of disease progression. Radiotherapy to target lesions is prohibited.
(7) The administration of anti-inflammatory or narcotic analgesic medications is permitted provided that no known or anticipated drug interactions exist and such medications are not included in the list of prohibited concomitant therapies.

4.4.2 Prohibited medications / Permitted medications – Use with caution
(1) Investigational agents other than tislelizumab and lenvatinib.
(2) Other systemic anticancer therapies, including chemotherapy, immunotherapy, biologic therapy, and Chinese herbal preparations with antitumor properties.
(3) Surgical intervention for tumor lesions is prohibited. If surgery is strongly justified by clinical necessity, the timing between the procedure and investigational product dosing must be optimized to avoid interference with wound healing and the evaluation of unexplained bleeding. It is recommended to withhold the investigational product for at least 1 week prior to surgery. Resumption of dosing after surgery should be based on clinical assessment of wound healing and recovery.
(4) Immunosuppressants or immunostimulants, including chronic systemic use of corticosteroids at doses >10 mg/day prednisone (or equivalent) for palliative or supportive purposes, except when used for the management of investigational product-related adverse events.
(5) Vaccination with live vaccines, including but not limited to those for measles, mumps, rubella, varicella, yellow fever, rabies, BCG, and typhoid, is prohibited. Administration of inactivated vaccines for infectious disease prevention (e.g., pneumococcal, influenza, COVID-19 vaccines) is permitted but must be discussed with the investigator prior to use.
(6) Caution is advised regarding the use of medications with known or potential arrhythmogenic effects.
(7) Caution is advised regarding the use of anticoagulants or thrombo-prophylactic agents to avoid exacerbating potential bleeding risks. These include but are not limited to: salicylate derivatives (e.g., aspirin), heparin-based anticoagulants (e.g., low molecular weight heparin, enoxaparin), and prophylactic antithrombotic agents for secondary prevention of cardiovascular events (e.g., clopidogrel, ticagrelor). If use is deemed necessary, close monitoring of platelet counts and coagulation parameters is mandatory.

### 4.5 Contingency for Surgical or Radiation Intervention

Patients who demonstrate significant responses to the treatment might raise the question about cytoreductive nephrectomy or lymphadenectomy or cytoreductive radiotherapy. The investigators will have a in-depth discussion with patients about the benefits and risks of particular interventions. If the patient decides to undergo surgery or radiotherapy against the predefined target lesion(s), he or she will be treated as withdrawal from the treatment study. The last imaging assessment and time of the last dose of investigation agents before surgery will be recorded to evaluate best response and duration of response. The overall survival will still be recorded, while tumor response and progression-free survival cannot be assessed after intervention against target lesion.

One week of washout period is required in light of the fact that wound healing might be affected by the side effect of lenvatinib. If there is clinically relevant neutropenia or thrombocytopenia, or other abnormal laboratory parameters that in the opinion of the surgeon or study physician will have a negative impact on surgical outcome, patients will need these values corrected prior to surgery. If there is evidence or potential of continued benefit, investigators will discuss with the patient resuming the combination treatment either as adjuvant therapy or advanced-stage therapy. The drug will restart 4 weeks post-surgery or at a later date if patient requires further postoperative recovery.

### 4.6 Withdrawal or Termination

4.6.1 Subject withdraw from the study
(1) Withdrawal of informed consent and refusal to continue follow-up.
(2) The subject will be withdrawn from the study if, in the investigator's judgment, continued participation is no longer in the subject's best interest due to any of the following: adverse reaction(s) or intolerable toxicity, clinically significant abnormal laboratory finding(s), intercurrent illness(es), et al.
(3) Disease progression requiring immediate disruption of the current treatment and other interventions.
(4) Patients' decision to receive surgery, radiotherapy, or any other local intervention for preset target lesions.
(5) Loss of follow-up.
(6) Death of any cause.

4.6.2 Termination of study treatment
(1) Withdrawal of informed consent.
(2) Radiographic or clinical evidence suggestive of disease progression, unless the subject meets all predefined criteria for treatment continuation beyond progression and provide consent of continuation.
(3) Any urgent medical situation requiring immediate intervention and cessation of study treatment.
(4) Pregnancy event in a female subject during the study.
(5) Any adverse reactions, laboratory abnormalities, or other medical conditions that may preclude potential benefit from continued treatment with investigation agents.
(6) Deterioration of the subject's general health condition to an extent that prevents continued participation in the study.

(7) Subject ineligibility identified after enrollment, or significant protocol deviations such as non-compliance.
(8) Loss to follow-up or death of any cause.
(9) Completion of 24 months of combination therapy with lenvatinib plus tislelizumab, or monotherapy with either agent.
(10) Initiation of any other anti-tumor therapy prohibited by the study protocol.
(11) Any other reason that, in the investigator's opinion, justifies discontinuation of investigation agents
(12) Termination at the request of the sponsor, investigator, or regulatory authorities.

### 4.7 Criteria for Treatment Continuation beyond Disease Progression

In immunotherapy like tislelizumab, increased infiltration of immune cells may lead to apparent tumor enlargement or the emergence of new lesions, resulting in pseudo-progression. Some patients with renal cancer may derive clinical benefit even after progression on targeted therapies or immunotherapy. Given the limited alternative treatment options and potential toxicity concerns following disease progression, subjects with initial radiologically confirmed disease progression per RECIST 1.1 may continue study treatment—if the investigator judges that continued therapy is likely to provide clinical benefit according to protocol-specified criteria and after obtaining fully informed consent. A subsequent radiologic assessment must be performed within 4–8 weeks to confirm progression status (Further progression is defined as: An additional ⩾10% increase in tumor burden from the initial progression in the absence of new lesions; or, an additional ⩾10% increase in the sum of target lesion diameters and measurable new lesions (⩽5) from the time of initial progression, plus an absolute increase ⩾5 mm in the sum of diameters of new lesions (⩽5); or, the emergence of new lesions again). Upon confirmation of further progression, study drug treatment must be permanently discontinued.

Subjects may continue study treatment after initial progression only if all of the following criteria are met:
(1) Absence of clinical signs or symptoms indicative of disease progression (including clinically significant deterioration in laboratory examinations).
(2) No decline in ECOG performance status.
(3) No evidence of rapid progression at critical anatomical sites which require urgent medical intervention (e.g., new or worsening CNS metastases causing sensorimotor deficits or other neurological emergencies).

The investigator must thoroughly explain to the subject the potential risks and benefits of continuing study treatment after disease progression and disclose all currently available standard therapeutic options or alternative treatments. All related information must be accurately documented in the source medical records.

## 5 DOSE DELAY OR MODIFICATION

Medication adjustments will be based on drug-related AEs as judged by the investigator. According

to the judgment of the investigator, the dosing of individual study drugs may be managed independently based on their suspected relationship to an AE. Only the drug(s) implicated in the toxicity should undergo dose modification (reduction, interruption/delay, or discontinuation). If the AE cannot be definitively attributed to only a subset of the study drugs, the dosing of all study drugs should be adjusted accordingly.

Dose modifications for the subsequent cycle will be based on the highest-grade AE observed in the current cycle; treatment may only resume once the relevant AE has resolved to Grade 0-1 (with exceptions for alopecia, Grade 2 fatigue, and endocrine AEs ≤ Grade 2 controlled by hormone replacement therapy). If only the suspected drug(s) are interrupted/delayed, the non-suspended drug(s) should continue as scheduled, with the full regimen resuming upon toxicity resolution.

**5.1 Dose Modification of Tislelizumab**

Table 2: Modification due to Common Adverse Events
(Refer to NCCN Guidelines Version 1.2024 Management of Immune Checkpoint Inhibitor-Related Toxicities if needed)

| irAE | Severity (NCI CTCAE 5.0) | Treatment Adjustment |
|---|---|---|
| Pneumonia | Grade 2 | Hold immunotherapy until improvement to grade 0-1 |
| | Grade 3-4 or recurrent Grade 2 | Permanent discontinuation |
| Diarrhea and colitis | Grade 2-3 | Hold immunotherapy until improvement to grade 0-1 |
| | Grade 4 | Permanent discontinuation |
| Hepatitis | Grade 2, AST or ALT ≤ 3-5 × ULN, or/and TBIL ≤ 1.5-3 × ULN | Hold immunotherapy until improvement to grade 0-1 |
| | Grade 3, AST or ALT ≤ 5-20 × ULN, or TBIL ≤ 3-10 × ULN<br>Grade 4, AST or ALT>20 × ULN, or TBIL>10 × ULN | Permanent discontinuation |
| nephritis | Grade 2 or 3 blood Cr increased | Hold immunotherapy until improvement to grade 0-1 |
| | Grade 4 blood Cr increased | Permanent discontinuation |
| hypophysitis | Grade 2-3 | Hold immunotherapy until improvement to grade 0-1 |
| | Grade 4 | Permanent discontinuation |
| Thyroid disease | Grade 2-3 hypothyroidism<br>Grade 2-3 hyperthyroidism | Hold immunotherapy until improvement to grade 0-1 |
| | Grade 4 hypothyroidism<br>Grade 4 hyperthyroidism | Permanent discontinuation |
| Adrenal insufficiency | Grade 2 | Hold immunotherapy until improvement to grade 0-1 |

| | | |
|---|---|---|
| | Grade 3-4 | Permanent discontinuation |
| High blood sugar or type I diabetes | Grade 3 | Suspend dosing until improvement to grade 0-1 |
| | Grade 4 | Permanent discontinuation |
| Skin adverse reaction | Grade 3 | Suspend dosing until improvement to grade 0-1 |
| | Grade 4, Stevens-Johnson syndrome or toxic epidermal necrolysis | Permanent discontinuation |
| Thrombocytopenia | Grade 3 | Suspend dosing until improvement to grade 0-1 |
| | Grade 4 | Permanent discontinuation |
| Other irAE | Grade 3-4 elevated blood amylase or elevated lipase<br>grade 2-3 pancreatitis<br>Grade 2 myocarditis<br>grade 2 encephalitis<br>Other irAE with first occurrence of grade 2-3 | Suspend dosing until improvement to grade 0-1 |
| | Grade 4 pancreatitis or any grade of recurrent pancreatitis<br>Grade 3-4 myocarditis<br>Grade 3-4 encephalitis<br>Grade 4 First occurrence of other irAE | Permanent discontinuation |
| Recurrent or persistent irAE | Recurrent Grade 3 or 4 (except endocrine disease)<br>Grade 2 or 3 AE did not improve to Grade 0 to 1 within 12 weeks after last dose (excluding endocrine disease)<br>Corticosteroids fail to decrease to ≤ 10mg/day prednisone equivalent within 12 weeks after last dose | Permanent discontinuation |
| infusion reaction | Grade 2 | Decrease the drip rate or suspend the administration. When the symptoms are relieved, resume the medication and closely observe. |
| | Grade 3-4 | Permanent discontinuation |

### 5.2 Dose Modification of Lenvatinib

Mild to moderate AEs (Grade 1 to 2) generally do not require holding unless the patient remains intolerable after active supporting treatment. Severe (⩾Grade 3) or intolerable Grade 2 AEs require dose interruption until the AEs improve to Grade 0 to 1 or baseline.
Intolerable Grade 2 AEs are defined as those that, despite optimal supportive care, significantly interfere with daily activities or require persistent dose interruption.

Table 3: Modification Principles for Lenvatinib due to Common Adverse Events

| Treatment-related AEs | Processing | Dose Adjustment |
|---|---|---|
| | Grade 1 AEs or Tolerable Grade 2 AEs | |
| | Continue treatment | Dose unchanged |
| | Intolerable Grade 2 or Grade 3 AEs | |
| First occurrence | Suspend lenvatinib until remission to Grade 0-1 or tolerable Grade 2 | Decreased lenvatinib dose to 16mg QD |
| Second occurrence (same AE or newly-onset AE) | Suspend lenvatinib until remission to Grade 0-1 or tolerable Grade 2 | Reduce the dose of lenvatinib to 12mg QD or 8mg/12mg QOD |
| Third occurrence (same AE or newly-onset AE) | Suspend lenvatinib until remission to Grade 0-1 or tolerable Grade 2 | Reduce the dose of lenvatinib to 8mg QD |
| The Fourth Occurrence (same AE or newly-onset AE) | Suspension of lenvatinib | Discuss with the patient whether to discontinue |
| | Grade 4 AEs | |
| | Discontinuation of investigational drug therapy | |

Table 4: Medication Adjustment Scheme for Common Adverse Events of Lenvatinib

| AEs | Severity | Processing | Reduction and resumption of lenvatinib treatment |
|---|---|---|---|
| Hypertension | Grade 3 (despite optimal antihypertensive therapy) | Pause | Mitigate to Grade 0-2 |
| | Grade 4 | Drug withdrawal | Do not restart treatment |
| proteinuria | ⩾ 2g/24 hours | Pause | Remission to <2g/24 hours |
| Nephrotic syndrome | | Drug withdrawal | Do not restart treatment |
| Renal insufficiency | Grade 3 | Pause | Remission to Grade 0-1 or Baseline |
| | Grade 4 | Drug withdrawal | Do not restart treatment |
| Cardiac insufficiency | Grade 3 | Pause | Remission to Grade 0-1 or Baseline |

| | | | |
|---|---|---|---|
| | Grade 4 | Drug withdrawal | Do not restart treatment |
| Posterior reversible encephalopathy syndrome (PRES)/Reversible posterior leukoencephalopathy syndrome (RPLS) | Any grade | Pause | If remission is to Grade 0-1, consider restarting treatment with a reduced dose. |
| Liver toxicity | Grade 3 | Pause | Remission to Grade 0-1 or Baseline |
| | Grade 4 | Drug withdrawal | Do not restart treatment |
| Arterial thromboembolism | Any grade | Drug withdrawal | Do not restart treatment |
| Bleeding | Grade 3 | Pause | Mitigate to Grade 0-1 |
| | Grade 4 | Drug withdrawal | Do not restart treatment |
| Gastrointestinal perforation or fistula | Grade 3 | Pause | Remission to Grade 0-1 or Baseline |
| | Grade 4 | Drug withdrawal | Do not restart treatment |
| Non-gastrointestinal fistula | Grade 4 | Drug withdrawal | Do not restart treatment |
| QT interval prolongation | > 500ms | Pause | Remission to ≤ 480ms or baseline |
| Diarrhea | Grade 3 | Pause | Remission to Grade 0-1 or Baseline |
| | Grade (despite medical management) | Drug withdrawal | Do not restart treatment |

When a Grade ≥3 or intolerable Grade 2 AE considered possibly related to lenvatinib occurs, lenvatinib is temporarily withheld. Treatment could be resumed only after the AE resolves to Grade 0-1 or baseline, at which point a dose reduction is required. Dose reduction is allowed stepwise from 20 mg to 16 mg, then to 12 mg, and then to 8 mg (20 mg → 16 mg → 12 mg → 8 mg). Skipping dose levels is not permitted. Subjects who could not tolerate the 8 mg dose would be suggested discontinued from the study.

In principle, subjects who receive a dose reduction will continue on the reduced dose in subsequent cycles. However, while on a reduced dose, if the investigator judge that there would be a risk or possibility of disease progression, and the subject is considered stable for safety (i.e., no Grade ≥ 3 AE or intolerable Grade 2 AE), dose re-escalation by one level (i.e., +4 mg, no skipping) is allowed. Each subject is permitted at most one dose re-escalation. If, after re-escalation, the subject again develops a Grade ≥3 or intolerable Grade 2 AE, the protocol-mandated dose interruption is applied, and no further dose re-escalation is allowed after subsequent dose reduction.

## 6 CLINICAL AND LABORATORY EVULATIONS

### 6.1 Study Calendar (Table 5)

| Study Steps/Visits | Screening period (-28 days) | | | Treatment period (drug treatment every 3 weeks cycle) | | | | | | | | End of C1-8 visit (± 3) [4] & treatment continue | Follow-up period (every 3 months ± 14 days) | |
|---|---|---|---|---|---|---|---|---|---|---|---|---|---|---|
| | Within D-28 | Within D-14 | Within D-7 | C1 D1 | C2 D1 (± 3) | C3 D1 (± 3) | C4 D1 (± 3) | C5 D1 (± 3) | C6 D1 (± 3) | C7 D1 (± 3) | C8 D1 (± 3) | | Safety follow-up [5] | Survival follow-up[6] |
| Sign Informed Consent Form | √ | | | | | | | | | | | | | |
| Demographic data | √ | | | | | | | | | | | | | |
| Tumor diagnosis | √ | | | | | | | | | | | | | |
| Previous history of cancer and treatment | √ | | | | | | | | | | | | | |
| Concomitant disease | √ | | | | | | | | | | | | | |
| Vital Signs | | | √ | √ | √ | √ | √ | √ | √ | √ | √ | √ | √ | |
| ECOG Score | | | √ | | √ | √ | √ | √ | √ | √ | √ | √ | √ | |
| Physical examination | | | √ | | √ | √ | √ | √ | √ | √ | √ | √ | √ | |
| CBC | | | √ | | √ | √ | √ | √ | √ | √ | √ | √ | √ | |
| Urine analysis | | | √ | | √ | √ | √ | √ | √ | √ | √ | √ | √ | |
| Metabolic panel | | | √ | | √ | √ | √ | √ | √ | √ | √ | √ | √ | |
| Coagulation function | | | √ | | | √ | | √ | | √ | | √ | √ | |
| Pregnancy test | | | √ | | | | | √ | | | | √ | | |

| | | | | | | | | | | | | | | |
|---|---|---|---|---|---|---|---|---|---|---|---|---|---|---|
| Thyroid function | | √ | | | | √ | | √ | | √ | | √ | √ | |
| 12-lead ECG | | √ | | | | √ | | √ | | √ | | √ | √ | |
| Echocardiography | | √ | | | | | | | | | | | | |
| Virus Check | √ | | | | | | | | | | | | | |
| Chest CT [1] | √ | | | | | √ | | √ | | √ | | √ | | √ |
| Contrast-enhanced CT or MRI [1] of the abdomen | √ | | | | | √ | | √ | | √ | | √ | | √ |
| Contrast-enhanced Brain MRI [1] | √ | | | | | √ | | √ | | √ | | √ | | √ |
| Other imaging [1] (as needed) | √ | | | | | √ | | √ | | √ | | √ | | √ |
| Tumor tissue collection [2] | √ | | | | | | | | | | | | | |
| Blood and urine specimen collection [2] | | | √ | | √ | √ | √ | √ | √ | √ | √ | √ | | √ |
| Checking inclusion/exclusion criteria | | | √ | | | | | | | | | | | |
| Tislelizumab administration | | | | √ | √ | √ | √ | √ | √ | √ | √ | | | √ |
| Lenvatinib accountability | | | | √ | √ | √ | √ | √ | √ | √ | √ | | | |
| Lenvatinib administration | | | | Continuous oral, 20mg QD or reduced dose according to AE | | | | | | | | | | |
| AE [3] evaluation and record | √ | | | √ | √ | √ | √ | √ | √ | √ | √ | √ | √ | |
| Concomitant medication or concomitant therapy | √ | | | √ | √ | √ | √ | √ | √ | √ | √ | √ | √ | |
| Subsequent treatment | | | | | | | | | | | | √ | √ | √ |
| Survival follow-up | | | | | | | | | | | | | | √ |

Note: 1. Tumor Imaging and Efficacy Assessment: Tumor response is assessed according to RECIST 1.1. Imaging examinations include contrast-enhanced CT or MRI of the chest, abdomen, pelvis, and any other areas with known lesions. During the screening period, contrast-enhanced MRI or CT of the brain and a bone scan are also required, which can be replaced by PET-CT. The imaging techniques used for subsequent assessments must be consistent with those used at baseline. A routine tumor imaging assessment performed within 28 days prior to the first dose of the investigation agents (after signing the informed consent form) is acceptable for screening. If this assessment meets the requirements for evaluation, repeat during the screening period is not required. A bone scan performed within 42 days prior to the first dose is acceptable for the screening period. During the treatment period, tumor assessments are performed every 6 weeks for the first 24 weeks, followed by every 12 weeks thereafter. These assessments will continue until one of the following events occurs, whichever comes first: withdrawal of consent, loss to follow-up, disease progression, death, or study termination. Additional imaging of other sites with known or suspected lesions is performed when clinically indicated. Tumor imaging and efficacy assessment should also be performed when disease progression is suspected and upon treatment discontinuation, if no tumor assessment has been conducted within the preceding 4 weeks.

2. Subjects participating in the exploratory study should provide archived or fresh tumor tissue specimens before enrollment and take blood and urine specimens at baseline and time points required by the protocol.

3. Adverse Events (AEs): AEs will be collected from the first dose of the study drug until 90 days after the last dose of the investigation agents or until the initiation of new anticancer therapy, whichever occurs first. Exception: If treatment with tislelizumab is discontinued and the subject continues on lenvatinib monotherapy, the AE collection period for that subject will be until 30 days after the last dose of lenvatinib or the start of new anticancer therapy, whichever occurs first. All AEs will be graded for severity according to the National Cancer Institute Common Terminology Criteria for Adverse Events (NCI CTCAE) version 5.0.

4. A comprehensive assessment is required at the end of treatment visit, which must occur within 3 days of the treatment discontinuation decision. This assessment will include all scheduled examinations, with the exception of those that have been performed within the following timeframes: tumor imaging within 4 weeks and other tests within 7 days.

5. Safety Follow-up Period: The Safety follow-up period is defined as the time from the last dose of the study drug(s) until 90 days (±7 days) after the last dose of the investigational product(s), or until the initiation of new anticancer therapy, whichever occurs first. Exception: If treatment with tislelizumab is discontinued and the subject continues on lenvatinib monotherapy, the follow-up period for that subject will be until 30 days (±7 days) after the last dose of lenvatinib, or the start of new anticancer therapy, whichever occurs first. Subjects must return to the study site within 30 days (±7 days) after the last dose of the study drug(s) or before starting new anticancer therapy to complete the protocol-specified safety assessments. Subjects are strongly encouraged to return to the study site for safety assessments at Day 60 and Day 90 (±7 days) after the last dose of the investigational product(s). For subjects who are unable to return, a telephone follow-up should be conducted to collect safety information, including: outcome of ongoing AEs, newly occurring AEs and SAEs, concomitant medications.

6. Survival follow-up will commence after the last dose of the study treatment and continue at 3-month intervals (±14 days). Information regarding survival and subsequent anticancer treatments will be collected through telephone contact or other methods.

### 6.2 Pretreatment Evaluation

All screening assessments must be completed and results reviewed to confirm that patients met all inclusion criteria without any exclusion criteria prior to receiving study treatment. The investigator records the information of all the screened patients, the screening examination and results, confirms the qualification of the patient or records the reasons for the screening failure, etc. Abnormal screening results involving the enrollment are only allowed to be repeated once during the screening period, and the results closest to the first dose of trial drug are used to determine whether the patient can be enrolled. Subjects with previous screening failures are allowed to be screened again, and the investigator will evaluate the patient's eligibility based on the latest screening evaluation results. All items shall be completed within 28 days before the first dose of medication unless otherwise specified.

[Signed Informed Consent] Subject's signed informed consent must be obtained prior to any study procedure, except for tumor imaging examinations that meet the time limits.

[Demographic data] including gender, date of birth, nationality, etc.

[Tumor diagnosis] including clinical diagnosis, pathological diagnosis, gene diagnosis, diagnosis time, clinical pathological stage, primary and metastatic sites.

[Previous history of tumor and treatment] History of tumor: first clinical diagnosis, pathological diagnosis, time of first diagnosis, etc; History of surgery for primary lesion and/or metastatic lesion, including operation time, operation method (radical/cytoreductive) and whether there was neoadjuvant therapy or adjuvant therapy; Drug treatment history: Chemotherapy, targeted drugs, cytokine treatment history, including medication regimen, time, dose, optimal efficacy and time to disease progression or recurrence after the last systemic treatment; Radiotherapy History: including radiotherapy method, time, dose and site (systemic/local);

[Concomitant diseases and related treatment] such as diabetes, hypertension and other chronic diseases.

[Vital signs] including body temperature, heart rate, respiratory rate, and blood pressure. Completed within 7 days prior to the first dose.

[ECOG Score] Completed within 7 days prior to the first trial medication.

[Physical examination] A comprehensive physical examination performed during the screening period and at the end of the study, which was completed within 7 days before the first trial medication, and the weight and height were measured at the first visit.

[Complete blood count], [urine routine], [metabolic panel], [coagulation function], [pregnancy test] Completed within 7 days before the first administration.

[Thyroid function], [12-lead electrocardiogram], [echocardiography] Completed within 14 days before the first administration.

[Virus examination] Completed within 28 days before the first administration.

[Tumor imaging examination] Including head contrast-enhanced MRI or enhanced CT, chest CT (contrast-enhanced or plain scan), abdominal enhanced CT or MRI and bone scan. Tumor imaging assessments should be performed within 28 days prior to the first dose and bone scan is allowed to be performed within 42 days prior to the first dose.

[Tumor tissue collection] Subjects participating in the exploratory study are required to provide tumor tissue from previous archived or fresh tumor tissue samples, which is expected to completed within 28 days before the first trial.

[Check the inclusion and exclusion criteria] Subjects can only be enrolled in the study after meeting all the inclusion conditions, and without any exclusion criteria.

[Adverse events] After signing the informed consent form and before the first medication, only AE leading to the subject's out of the group or death is reported, and other events are recorded as medical history.

[Concomitant medication/concomitant treatment] All medications within 28 days prior to the first medication must be recorded in the CRF, including the generic name of the drug, dose, reason for use, start time and end time.

[Urine analysis] If urine protein ⩾ 2 + before medication, or if the researcher judges that the result is abnormal and with clinical significance, additional 24-hour urine protein quantification should be detected. Subjects with urine protein quantification ⩾ 2g during screening are not recommended to be enrolled.

### 6.3 Evaluation during Treatment

The treatment period begins on the day of the first dose of the investigational product and continues until the completion of the study treatment. Eligible subjects who enter the treatment period will have their subsequent visit and tumor imaging assessment schedules calculated based on the date of the first dose. The window for all treatment period visits is ±3 days. The window for tumor imaging assessments is ±7 days.

For subjects taking lenvatinib outside the hospital, the following information should be recorded by the subject in the Patient Diary Card:

Drug administration, missed doses, and dosing errors.

Blood pressure monitoring results.

Any self-perceived discomfort or symptoms.

The diary card must be submitted to the investigator at each clinic visit. The investigator will then assess medication compliance and safety based on these records. Following this assessment, the investigational product and a new diary card for the next treatment cycle will be dispensed to the subject.

[Vital signs] include body temperature, heart rate, respiratory rate, and blood pressure. Vital signs should be checked 30 minutes before each infusion and within 60 minutes after the infusion, and at the end of treatment visit. Blood pressure of the subject should be monitored daily when taking lenvatinib outside the hospital and recorded on the medication diary card.

[physical examination] During the treatment period, physical examinations will be limited to targeted assessments, with the exception of body weight, which must be measured at every visit.

[ECOG score], [CBC], [urine analysis], [metabolic panel], [coagulation function] ECOG score, CBC, urine analysis and metabolic panel carried out every 3 weeks in the first 24 weeks and every 12 weeks after 24 weeks. If hematological toxicity leads to interruption of medication or dose adjustment, the researchers can adjust the examination frequency by themselves. Coagulation were performed every 6 weeks for 24 weeks and every 12 weeks after 24 weeks.

[Pregnancy test] Female subjects of childbearing potential will have a urine pregnancy test every 12 weeks after starting drug treatment, and a serological pregnancy test as needed.

[Thyroid function] Every 6 weeks after the start of study drug treatment in the initial 24 weeks, every 12 weeks after 24 weeks, and also at the end of treatment visit and safety follow-up visit.

[12-lead ECG] After the start of study drug treatment, an examination (frequency is the same as thyroid function) performed and QTc calculated according to Bazett's consensus, as well as when clinically indicated.

[Echocardiography] Including LVEF assessment, examination after initiation of study drug therapy (once per year) and when clinically indicated.

[Imaging examination] Tumor imaging examination and efficacy assessment are carried out according to RECIST 1.1. Imaging examinations include chest CT, upper and lower abdominal enhanced CT or enhanced MRI, etc., according to the specific circumstances, using the same method as the baseline to ensure that the baseline of the target lesion and the data during the treatment period can be collected completely. Imaging and efficacy assessments are performed every 6 weeks (± 7 days) for the first 24 weeks after initiation and every 12 weeks (± 7 days) after 24 weeks until disease progression, loss to follow-up, death, withdrawal of consent, initiation of other antitumor therapy, or study termination. Other sites with known or suspected lesions (eg, brain, bone) should be examined by imaging when clinically indicated, and unscheduled tumor assessments should be performed in subjects with suspected disease progression before the next scheduled oncology assessment.

[Study drug treatment] The study examination specified in the protocol must be completed before each cycle of administration, and investigation agents can be dispensed only after the safety evaluation completed.

[Adverse events] and [Concomitant medication] Continuously monitored and recorded.

### 6.4 Evaluation at the End of Treatment

When a subject discontinues study treatment or withdraws from the study, all protocol-specified assessments must be completed in End-of-Treatment (EOT) Visit. Waiver of repeat examinations: assessments do not need to be repeated if valid results are available from the same tests performed within 7 days prior to treatment discontinuation (for non-radiologic assessments), or tumor imaging assessment performed within 4 weeks prior to study termination.

Early termination visit: If the decision to discontinue treatment is made during a scheduled visit and before the initiation of the next treatment cycle, that visit may be designated as the EOT Visit.

EOT Visit evaluation items included: [Vital signs], [comprehensive physical examination], [ECOG score], [CBC], [urine analysis], [metabolic panel], [coagulation function], [pregnancy test], [thyroid function], [12-lead electrocardiogram], [imaging examination], [adverse events], [concomitant medication], [subsequent anti-tumor treatment].

### 6.5 Evaluation during Follow-up

Safety follow-up: 90(± 7) days after the last dose of study drug (30 days after the last dose of lenvatinib if lenvatinib alone maintained in the treatment) or before the initiation of new anti-tumor

therapy (whichever occurs first). Safety visits should include [vital signs], [physical examination], [ECOG score], [12-lead electrocardiogram], [laboratory examination] and assessment of AEs, concomitant medication and treatment. Additional visits may be added as needed for management of AEs.

Survival follow-up: survival follow-up will commence after the last dose of the investigational product and be conducted every 3 months (±14 days). Information regarding survival status and subsequent anti-tumor therapies will be collected via telephone or other appropriate means until death, withdrawal of consent, loss to follow-up, or study termination.

Continue imaging examination after the end of treatment: If a subject discontinues treatment for reasons other than disease progression, initiation of new anti-tumor therapy, withdrawal of consent, loss to follow-up, or study termination, and has no evidence of radiographic progression at the time of treatment cessation, radiographic assessments must continue per the protocol-specified schedule until one of the following occurs (whichever comes first): radiographic progression as per RECIST 1.1, initiation of new anti-tumor therapy, withdrawal of consent, loss to follow-up, or study termination.

# 7 ADVERSE EVENTS AND REPORTING REQUIREMENTS

## 7.1 Definition

An Adverse Event (AE) is any untoward medical occurrence in a patient or clinical trial subject administered a medicinal product. It does not necessarily have to have a causal relationship with this treatment. An AE can therefore be any unfavorable and unintended sign (including an abnormal laboratory finding), symptoms, or disease temporally associated with the use of an investigational product, whether or not considered related to the product. This includes, but is not limited to:
(1) Exacerbation of a pre-existing illness or condition.
(2) An abnormal laboratory value that is not part of the investigational product's intended therapeutic effect.
(3) Any accidental injury occurring during the subject's participation in the trial (e.g., a fracture from a fall).

The investigator should record in detail any AE occurring in the subject, including description of AE and all related symptoms, time of occurrence, severity, cause of AE, correlation with experimental drugs, duration, measures taken, and final results and outcomes.

A serious adverse event (SAE) is an adverse medical event that meets one or more of the following criteria:
(1) The events leading to the death.
(2) Life-threatening events (the subject is in immediate danger of death at the time of the event).
(3) Events requiring hospitalization or prolonged hospitalization.
(4) Events that may result in permanent or severe disability/dysfunction/affecting the ability to work.
(5) Congenital abnormalities or birth defects.

(6) Other: Although not immediately life-threatening, resulting in death or hospitalization, it may be harmful to the subject in accordance with reasonable medical and scientific judgment, or intervention may be required to prevent serious consequences, including but not limited: allergic bronchospasm requiring intensive treatment in the emergency or at home, hematological cachexia or convulsions not requiring hospitalization, potential drug-induced liver damage, suspected transmission of pathogens through study drugs, secondary tumors, etc.

Waiver of SAE reporting requirements:
(1) When death caused by disease progression is the endpoint of efficacy, death caused by disease progression is not required to be reported as SAE.
(2) The following specific hospitalizations and prolonged hospital stays do not need to be reported as SAE: during the visit and hospitalization specified in the protocol during the clinical trial, no new AE occurred and no aggravation of the original disease; Elective hospitalization unrelated to AE deterioration; Hospitalization for management reasons; Hospitalization due to medical insurance reimbursement, etc.

**7.2 AE/SAE Records and Reports**

In the pre-treatment period (after ICF signing until before first dose), only all SAEs will be recorded and reported. In the treatment and follow-up Period (after initiation of study treatment), all AEs and SAEs will be reported. The reporting period for AEs continues until 90 days (±7 days) after the last dose of the combined regimen (tislelizumab + lenvatinib), or 30 days (±7 days) after the last dose of lenvatinib monotherapy (in cases where tislelizumab is discontinued), or the initiation of new anticancer therapy, whichever occurs first. In the post safety follow-up period, investigators are not required to actively solicit AEs. However, if an investigator becomes aware of an AE that is considered related to prior study treatment, it must be accurately recorded and reported.

For all AEs occurring during the course of the clinical study, the following actions should be taken:
(1) Take appropriate protective measures for the subjects immediately to ensure the safety of the subjects, and experts should make a diagnosis and state the reasons.
(2) If the study is terminated, the researcher should check the subjects regularly and fill in the date of termination of the study, the reason for termination and the detailed process in the CRF.
(3) The investigator should follow up all AEs until any of the following occurs: the AE is resolved or improved to the baseline level or better; the investigator confirms that the event is stable and no further improvement is expected; the subject dies; the subject is lost to follow-up or withdraws informed consent; the investigator confirms that the AE is not related to the study treatment; the subject starts a new anti-tumor treatment.

SAE reporting begins with the subject's signed informed consent until the end of the safety follow-up period. In the event of an SAE, whether it is the first report or a follow-up report, the investigator is required to complete the SAE report form and send it to the regulatory agency within 24 hours of being informed of the SAE. The SAE should include detailed documentation of symptoms, severity, association with study drug, time to onset, time to treatment, measures taken, time and manner of follow-up, and outcome. If the following important information of the ongoing SAE changes, the

investigator should submit a follow-up report immediately after 24 hours of knowledge: new symptoms, signs or changes in diagnosis; important new diagnostic test results; changes in causality based on new information; changes in event outcome (including recovery), etc. If the investigator believes that the previously reported SAE has misinformation, it can be corrected, withdrawn or downgraded in the follow-up report and reported in accordance with the SAE reporting procedures.

**7.3 Abnormal Laboratory Results**

Blood, urine, and other specimens will be collected according to the study schedule and analyzed by the central laboratory of this site. Not every laboratory abnormality constitutes an AE. Assessment should consider baseline values and clinical significance. An AE must be reported if a laboratory abnormality meets any of the following criteria:
(1) It is accompanied by clinical signs or symptoms.
(2) It leads to a modification of the study treatment (e.g., dose reduction, treatment interruption, or discontinuation).
(3) It requires medical intervention or a change in concomitant medication.
(4) It is considered clinically significant by the investigator.

**7.4 Disease Progression**

Disease progression (including fatal progression) that is an expected outcome in the study population and is measured as an efficacy endpoint should not be reported as an AE. Similarly, signs, symptoms, or clinical sequelae that are clearly attributable solely to disease progression do not need to be reported as AEs.

**7.5 Reporting of Death Events**

All deaths occurring during the study treatment period or within the protocol-defined safety follow-up period after the last dose must be reported as follows:
(1) Deaths due to disease progression: Deaths clearly attributable to disease progression are expected efficacy endpoints and should not be reported as an SAE. However, they must be documented on the Death Explanation page of the CRF, with the primary cause of death specified.
(2) Deaths not due to disease progression (or of unknown cause): If a death is not due to disease progression (or the cause is unclear), the AE leading to death must be reported as an SAE. It must also be recorded on the CRF's Death Explanation page. The report should include information on any potential disease progression and complications, specifying both the primary and secondary causes of death if applicable.
(3) Unexplained deaths during treatment/follow-up: Deaths from unknown causes occurring during the treatment period or the safety follow-up period must always be reported as an SAE and documented on the CRF's Death Explanation page. The investigator must make every effort to follow up and determine the cause of death.
(4) Deaths after the safety follow-up period: Deaths occurring after the safety follow-up period should be recorded on the Death Explanation page. If the death is due to an event that began after

the safety follow-up period but is suspected to be a result of delayed toxicity from the investigational product, it must be reported as an SAE according to the required reporting timelines.

### 7.6 Reporting of Pregnancy Events

All pregnancies occurring from the first dose until the end of the safety follow-up period must be reported.

Pregnancy in a female subject: The subject must be withdrawn from the study treatment. The pregnancy must be reported to the regulatory authority within 24 hours of the investigator becoming aware of it.

Pregnancy in a partner of a male subject: The subject may continue in the study. The pregnancy must be reported to the regulatory authority within 24 hours of the investigator becoming aware of it.

Pregnancy follow-up: The investigator must follow the outcome of the pregnancy until 1 month after delivery. The outcome must be reported to the regulatory authority.

Pregnancy Outcomes Considered SAEs: The following outcomes are considered SAEs and must be reported according to SAE timelines: stillbirth, spontaneous abortion, congenital anomaly/birth defect.

Concurrent SAEs: If any other SAE occurs during the pregnancy, it must also be reported according to the standard SAE reporting procedures.

### 7.7 Drug Overdose

A drug overdose is defined as the accidental or deliberate use of a dose that is larger than prescribed and medically significant. For this study, an overdose is defined as the administration of any investigational product at a dose exceeding 20% of the protocol-specified dose. A drug overdose itself, in the absence of any associated signs or symptoms, should not be reported as an AE. If the overdose results in any associated signs or symptoms, the corresponding AE or SAE must be recorded. In all cases, the overdose incident must be documented as a protocol deviation.

## 8 MEASUREMENTS OF EFFECT

### 8.1 Definitions

Best Overall Response (BOR) refers to the best response assessed by the investigator, recorded from the date of enrollment until the date of disease progression or the initiation of subsequent anti-cancer therapy (whichever occurs first). For each radiological assessment, two independent GU radiologists

will evaluate the treatment response according to the RECIST 1.1 criteria. For subjects who have no recorded disease progression and have not started subsequent anti-cancer therapy, the BOR will be determined based on all available response assessment results. For subjects who continue the original treatment regimen after disease progression, the BOR is determined based on the responses recorded up to the time of the initial progression. For subjects who achieve complete response (CR) or partial response (PR), the BOR result must be confirmed at the next scheduled tumor assessment according to RECIST 1.1. For stable disease (SD), the measurement must meet the SD criteria at least once after at least 6 weeks after treatment initiation.

The primary efficacy endpoint of this study is the objective response rate (ORR), as evaluated by RECIST 1.1 criteria, which refers to the proportion of subjects whose BOR is CR or PR among all enrolled subjects.

The secondary efficacy endpoints of the study were progression-free survival (PFS), overall survival (OS), duration of response (DOR), disease control rate (DCR), clinical benefit rate and safety.

PFS is defined as the interval between treatment initiation and the first occurrence of disease progression or death from any cause, whichever occurs first. Subjects who had no disease progression or death were censored on the date of the last valid tumor evaluation; subjects who did not undergo any in-study tumor evaluation and did not die were censored on the date of enrollment; subjects who did not report any disease progression and started any out-of-protocol anti-tumor therapy, censored treatment was performed on the date of the last evaluable tumor evaluation prior to the initiation of subsequent anti-tumor therapy.

OS is defined as the time from treatment start to death of any cause. Subjects with no recorded death will be censored on the last known date of survival, and those without any follow-up information will be censored on the day of enrollment.

DCR is defined as the proportion of subjects whose BOR is CR or PR or SD in all enrolled subjects.

DOR is defined as the time from the first documented CR or PR to the first documented disease progression (PD) or death from any cause.

The clinical benefit rate is defined as the percentage of patients who achieved sustained disease control for at least 24 weeks.

Upon maturity of the data, if the median OS or PFS has not been reached, the 1 or 2-year OS and 1 or 2-year PFS rates will be evaluated. The 1 or 2-year OS rate is defined as the proportion of subjects who were alive at 1 or 2 years after the treatment initiation using the Kaplan Meier method to estimate the 1-year or 2-year OS rates and 95% confidence intervals using the standard error derived from the Greenwood formula.

**8.2 Guidelines for Evaluation**

The efficacy measurement is conducted by imaging methods according to RECIST 1.1. Upon entering the survival follow-up phase, subjects shall be followed every 3 months (±14 days) to determine survival status through methods such as telephone contact. This continues until the subject's death, is lost to follow-up, withdraws consent, or the study is terminated.

Assessment of tumor response includes all known or suspected sites of disease. Imaging studies include CT or MRI of the chest, abdomen and lesions, brain CT or MRI for subjects with known or suspected brain metastases, and bone scan for subjects with known suspected bone metastases. Each imaging examination is evaluated by an independent imaging evaluation panel (composed of 2 GU radiologists from the Department of Radiology) to confirm the efficacy of the treatment.

Screening imaging evaluations will be performed within 28 days prior to the first dose, and imaging is planned to be performed every 6 weeks (± 7 days) for the first 24 weeks after the start of treatment, and every 12 weeks (± 7 days) after 24 weeks until disease progression, loss to follow-up, death, withdrawal of consent, subject's initiation of other anti-tumor therapy, or study termination.

Tumor assessment was not affected by dosing interruption or delay.

In cases where treatment is discontinued due to deterioration in the subject's overall health status without objective evidence of progression, the event should be reported as "symptomatic deterioration." Furthermore, every effort should be made to document objective progression (confirmed by imaging) even after the treatment has stopped. An assessment is also required whenever disease progression is suspected at the time of treatment discontinuation, if an assessment has not been performed within the previous 4 weeks.

**8.3 Imaging Scan Protocol**

Abdominal MRI was performed on a 3.0-T MRI scanner (Magnetom Prisma, Siemens Healthineers, Germany) with body coil transmission and 18-channel receiver coils. The non-enhancement protocol included the following sequences: axial T2-weighted fast spin-echo(repetition time, 4500 msec; echo time, 110 msec; FOV, 230×230 mm; section thickness, 3 mm; inter-section gap, 0.3 mm; matrix, 383× 383; NEX, 1; acquisition time, 2 min and 22 sec), axial T1-weighted Dixon gradient-echo (repetition time, 3.97; echo time1, 1.29 msec, echo time2, 2.52 msec; FOV, 380×310 mm; section thickness, 3 mm; matrix, 316×258; NEX, 1; acquisition time, 16 sec), and axial diffusion-weighted imaging (DWI) using a single-shot echo-planar imaging (SS-EPI) sequence (FOV, 320 × 210 mm2 ; matrix, 94 × 80; slice thickness, 3 mm; slices, 80; DW directions, 4; TR/TE, 6000/48 ms; b = 50 mm2 /s, 1000 mm2 /s; spectral adiabatic inversion recovery for fat suppression; acquisition time, 2 min 50 sec). Dynamic contrast enhancement was performed using axial T1-weighted Dixon gradient-echo (repetition time, 3.97; echo time1, 1.29 msec, echo time2, 2.52 msec; FOV, 380×310 mm; section thickness, 3 mm; matrix, 316×258; NEX, 1; acquisition time, 16 sec) following the intravenous administration of gadobutrol injection (Gadovist, Bayer Schering, 0.1-0.2 mL/kg) at a rate of 1.5-2.0 mL/s by power injection. Multiphasic contrast-enhanced MR sequences

were typically acquired at 20-25 sec, 40-60 sec, and 100-120 sec after contrast agent injection.

Abdominal CT examination was performed with helical scanners using triphasic Abdominal protocol with a CT system (TOSHIBA, Aquilion ONE TSX-301C, Japan). Patients were administered 400–500 mL of water orally 20 min before the examination. After unenhanced CT scans, arterial, venous and delayed phase images were then obtained beginning 30-35 sec, 60-70 sec, and 130-140 sec after injection of non-ionic contrast medium (Iopamiro, Bracco Imaging, or Iopromide, Bayer Schering Pharma) at a dose of 2 mL/kg. The imaging parameters of each phase were 120 kVp, 250-380-Ma tube current, a 5.0-mm slice thickness, and a reconstruction slice thickness of 1 mm.

All nonenhanced chest CT examinations were performed with helical scanners on CT systems (TOSHIBA, Aquilion ONE TSX-301C, Japan). The CT parameters included the following: tube voltage,120 kVp; tube rotation time, 0.6 seconds; tube current, 600 mA; helical pitch, 1.375; field of view, 500 mm; collimation, 40 mm; and slice thickness and interval for axial images, 5mm/5mm; reconstruction slice thickness, 1mm.

### 8.4 Biomarker Evaluation

The patient's EDTA anticoagulant peripheral blood 10ml and urine sample 20ml will be collected at the time points specified in the protocol before the treatment, during the treatment and at the end of the treatment visit. The biological specimens obtained will be stored in the biological specimen bank of Renji Hospital. Routine laboratory tests will be carried out in the central laboratory of Renji Hospital, mainly focusing on efficacy evaluation and safety monitoring.

For he exploratory investigation, LC-MS quantification will be applied to determine the levels of blood and urine succinate-modified small molecule metabolites (succinyl-adenosine and succinic-cysteine), to explore the correlation between metabolic marker levels and tumor burden (the sum of the longest diameters of target lesions evaluated by RECIST 1.1 criteria). The technical details of LC-MS quantification of plasma marker metabolites can be found in our previous report (***J Clin Invest***. 2023 Jun 1;133(11):e165028. doi: 10.1172/JCI165028.).

For exploratory biomarker research, archival tumor paraffin blocks or fresh biopsy specimens collected during the screening period (prior to treatment) will be analyzed by immunohistochemistry (IHC) for PD-L1 expression levels and lymphocyte subset infiltration. The objective is to determine their correlation with the efficacy of or resistance to the tislelizumab/lenvatinib combination therapy.

## 9 DATA AND PROTOCOL MANAGEMENT

All data, including subject screening information, medical history, baseline data, treatment administration details, adverse events (and their management/outcome), efficacy assessments, and follow-up records, are to be recorded by the investigator in the Case Report Forms (CRFs) and then entered into an electronic database. The original paper CRFs and the corresponding electronic datasets shall be retained at the Clinical Research Center of Renji Hospital, Shanghai Jiao Tong

University School of Medicine. In compliance with China's "Good Clinical Practice" (GCP), all original data pertaining to this clinical trial are required to be archived for a period of 5 years following the trial termination. The Principal Investigator will be the final arbiter of response, toxicity, or progression should a difference of opinion exist.

Patient Confidentiality: In order to maintain patient privacy, all database generated case report forms, study drug accountability records, study reports and communications will identify the patient by initials and the assigned patient number. The patient's confidentiality will be maintained and will not be made publicly available to the extent permitted by the applicable laws and regulations.

## 10 STATISTICAL CONSIDERATIONS

### 10.1 Sample Size

This study uses Simon's minimax two-stage design to evaluate the antitumor activity of tislelizumab plus lenvatinib. This design was selected because the study assesses a single pre-specified fixed-dose combination regimen, rather than multiple dose levels or competing regimens. Therefore, Simon's two-stage design was considered more appropriate than BOIN or BOP2 designs, which are mainly used for dose finding, dose optimization, or adaptive regimen selection.

Based on previous real-world data, first-line ICI/TKI or Beva/Erlo produced an ORR of approximately 25%–30% in patients with advanced FH-RCC. The null hypothesis was set as ORR $\leqslant$30% ($p_0$=0.30), and the alternative hypothesis was set as ORR $\geqslant$60% ($p_1$=0.60). With a one-sided α of 0.05 and 80% power, Simon's minimax two-stage design required 17 evaluable patients. In stage 1, 10 patients will be enrolled. If 2 or fewer objective responses are observed among the first 10 patients, the study will be stopped early for futility. Otherwise, an additional 7 patients will be enrolled in stage 2. The regimen will be considered promising if at least 9 objective responses are observed among 17 evaluable patients. This design yields an exact one-sided type I error rate of 4.02% under $p_0$=0.30 and a statistical power of 80.08% under $p_1$=0.60. To account for an anticipated 10%–20% attrition rate and ensure operational feasibility, the target enrollment is set at 20 patients. Statistical analyses were performed using R software (version 4.3.3).

### 10.2 Analysis Plan

The primary efficacy endpoint of the study is the investigator assessed ORR per RECIST 1.1. ORR is the proportion of subjects whose BOR is CR or PR among all enrolled subjects; the 95% confidence interval of ORR is calculated by Clopper-Pearson method.

The secondary efficacy endpoints of the study were progression-free survival (PFS), overall survival (OS), duration of response (DOR), disease control rate (DCR), clinical benefit rate and safety.

PFS is defined as the interval between treatment initiation and the first occurrence of disease progression or death from any cause, whichever occurs first. Subjects who had no disease progression or death are censored on the date of the last valid tumor evaluation; subjects who did not undergo any in-study tumor evaluation and did not die are censored on the date of enrollment;

subjects who did not report any disease progression and started any out-of-protocol anti-tumor therapy, censored treatment is performed on the date of the last evaluable tumor evaluation prior to the initiation of subsequent anti-tumor therapy. Median PFS is estimated with the Kaplan Meier method, and the 95% confidence interval of the median PFS is estimated with Greenwood method.

OS is defined as the time from entry to death from any cause. Subjects with no recorded death will be censored on the last known date of survival, and those without any follow-up information will be censored on the day of enrollment. OS is calculated using the same analytical method as PFS.

Upon maturity of the data, if the median OS or PFS has not been reached, the 1 or 2-year OS and 1 or 2-year PFS rates will be evaluated. The 1 or 2-year OS rate is defined as the proportion of subjects who were alive at 1 or 2 years after the treatment initiation using the Kaplan Meier method to estimate the 1-year or 2-year OS rates and 95% confidence intervals using the standard error derived from the Greenwood formula.

DOR is defined as the time from the first documented CR or PR to the first documented PD or death from any cause. Median DOR and the corresponding 95% confidence intervals will be calculated using the same analytical method as PFS.

DCR is defined as the proportion of subjects whose BOR is CR or PR or SD in all enrolled subjects. The clinical benefit rate is defined as the percentage of patients who achieved sustained disease control for at least 24 weeks. 95% confidence intervals of DCR and clinical benefit rate are calculated by Clopper-Pearson method.

The safety endpoints of this study is treatment-related AEs and SAEs (including laboratory abnormalities). Descriptive statistics will be used to summarize study drug exposure in the subject population, including duration of treatment, number of doses, and dose intensity; all treatment-related AEs and SAEs, severe AEs (Grade 3, 4, and 5), and the frequency of AEs leading to study drug interruption, discontinuation or dose reduction will be summarized. Deaths reported during study treatment and during follow-up after completion/discontinuation of treatment will be summarized.

Descriptive statistics is used to summarize the laboratory results, physical examination results, vital signs measurement results, weight, ECOG score, electrocardiogram results, and the number and proportion of abnormal changes after baseline.

Before receiving the test drug treatment, tumor tissue samples are examined for PD-L1 expression and lymphocyte abundance using immunohistochemical methods.

Blood and urine samples from patients at the time points specified in the protocol along the whole process of this study will be taken, and LC-MS quantification will be applied to determine the levels of blood and urine succinyl-adenosine and succinic-cysteine. The dynamic profiles of succinate-modified metabolite levels and the tumor burden of target lesions (as per RECIST assessment) will be illustrated graphically for each individual subject on a single plot, depicting their trajectories over

time relative to baseline. The collective trend of metabolic biomarker changes over the treatment period for the full evaluable population will also be illustrated on a single plot.

## Supplementary References

1. Moch H, Cubilla AL, Humphrey PA, Reuter VE, Ulbright TM. The 2016 WHO Classification of Tumours of the Urinary System and Male Genital Organs-Part A: Renal, Penile, and Testicular Tumours. *Eur Urol* 2016; **70**(1): 93-105.
2. Trpkov K, Hes O, Agaimy A, et al. Fumarate Hydratase-deficient Renal Cell Carcinoma Is Strongly Correlated With Fumarate Hydratase Mutation and Hereditary Leiomyomatosis and Renal Cell Carcinoma Syndrome. *Am J Surg Pathol* 2016; **40**(7): 865-75.
3. Zhang C, Li L, Zhang Y, Zeng C. Hereditary Leiomyomatosis and Renal Cell Cancer: Recent Insights Into Mechanisms and Systemic Treatment. *Front Oncol* 2021; **11**: 686556.
4. Trpkov K, Hes O, Williamson SR, et al. New developments in existing WHO entities and evolving molecular concepts: The Genitourinary Pathology Society (GUPS) update on renal neoplasia. *Mod Pathol* 2021; **34**(7): 1392-424.
5. Moch H, Amin MB, Berney DM, et al. The 2022 World Health Organization Classification of Tumours of the Urinary System and Male Genital Organs-Part A: Renal, Penile, and Testicular Tumours. *Eur Urol* 2022; **82**(5): 458-68.
6. Forde C, Lim DHK, Alwan Y, et al. Hereditary Leiomyomatosis and Renal Cell Cancer: Clinical, Molecular, and Screening Features in a Cohort of 185 Affected Individuals. *Eur Urol Oncol* 2020; **3**(6): 764-72.
7. Muller M, Ferlicot S, Guillaud-Bataille M, et al. Reassessing the clinical spectrum associated with hereditary leiomyomatosis and renal cell carcinoma syndrome in French FH mutation carriers. *Clin Genet* 2017; **92**(6): 606-15.
8. Chayed Z, Kristensen LK, Ousager LB, Ronlund K, Bygum A. Hereditary leiomyomatosis and renal cell carcinoma: a case series and literature review. *Orphanet J Rare Dis* 2021; **16**(1): 34.
9. Lau HD, Chan E, Fan AC, et al. A Clinicopathologic and Molecular Analysis of Fumarate Hydratase-deficient Renal Cell Carcinoma in 32 Patients. *Am J Surg Pathol* 2020; **44**(1): 98-110.
10. Xu Y, Kong W, Cao M, et al. Genomic Profiling and Response to Immune Checkpoint Inhibition plus Tyrosine Kinase Inhibition in FH-Deficient Renal Cell Carcinoma. *Eur Urol* 2023; **83**(2): 163-72.
11. Srinivasan R GS, Al HarthyM, et al. Results from a phase II study of bevacizumab and erlotinib in subjects with advanced hereditary leiomyomatosis and renal cell cancer (HLRCC) or sporadic papillary renal cell cancer. *J Clin Oncol* 2020; **38**(15): 5004.
12. Gleeson JP, Nikolovski I, Dinatale R, et al. Comprehensive Molecular Characterization and Response to Therapy in Fumarate Hydratase-Deficient Renal Cell Carcinoma. *Clin Cancer Res* 2021; **27**(10): 2910-9.
13. Sun G, Zhang X, Liang J, et al. Integrated Molecular Characterization of Fumarate Hydratase-deficient Renal Cell Carcinoma. *Clin Cancer Res* 2021; **27**(6): 1734-43.
14. Paschall AK, Nikpanah M, Farhadi F, et al. Hereditary leiomyomatosis and renal cell carcinoma (HLRCC) syndrome: Spectrum of imaging findings. *Clin Imaging* 2020; **68**: 14-9.
15. Nikolovski I, Carlo MI, Chen YB, Vargas HA. Imaging features of fumarate hydratase-deficient renal cell carcinomas: a retrospective study. *Cancer Imaging* 2021; **21**(1): 24.
16. Merino MJ, Torres-Cabala C, Pinto P, Linehan WM. The morphologic spectrum of kidney tumors in hereditary leiomyomatosis and renal cell carcinoma (HLRCC) syndrome. *Am J Surg Pathol* 2007; **31**(10): 1578-85.

17. Pan X, Zhang M, Yao J, et al. Fumaratehydratase-deficient renal cell carcinoma: a clinicopathological and molecular study of 13 cases. *J Clin Pathol* 2019; **72**(11): 748-54.
18. Llamas-Velasco M, Requena L, Adam J, Frizzell N, Hartmann A, Mentzel T. Loss of Fumarate Hydratase and Aberrant Protein Succination Detected With S-(2-Succino)-Cysteine Staining to Identify Patients With Multiple Cutaneous and Uterine Leiomyomatosis and Hereditary Leiomyomatosis and Renal Cell Cancer Syndrome. *Am J Dermatopathol* 2016; **38**(12): 887-91.
19. Buelow B, Cohen J, Nagymanyoki Z, et al. Immunohistochemistry for 2-Succinocysteine (2SC) and Fumarate Hydratase (FH) in Cutaneous Leiomyomas May Aid in Identification of Patients With HLRCC (Hereditary Leiomyomatosis and Renal Cell Carcinoma Syndrome). *Am J Surg Pathol* 2016; **40**(7): 982-8.
20. DiNardo CD, Propert KJ, Loren AW, et al. Serum 2-hydroxyglutarate levels predict isocitrate dehydrogenase mutations and clinical outcome in acute myeloid leukemia. *Blood* 2013; **121**(24): 4917-24.
21. Wang JH, Chen WL, Li JM, et al. Prognostic significance of 2-hydroxyglutarate levels in acute myeloid leukemia in China. *Proc Natl Acad Sci U S A* 2013; **110**(42): 17017-22.
22. Janin M, Mylonas E, Saada V, et al. Serum 2-hydroxyglutarate production in IDH1- and IDH2-mutated de novo acute myeloid leukemia: a study by the Acute Leukemia French Association group. *J Clin Oncol* 2014; **32**(4): 297-305.
23. Zheng L, Zhu ZR, Sneh T, et al. Circulating succinate-modifying metabolites accurately classify and reflect the status of fumarate hydratase-deficient renal cell carcinoma. *J Clin Invest* 2023; **133**(11).
24. Cancer Genome Atlas Research N, Linehan WM, Spellman PT, et al. Comprehensive Molecular Characterization of Papillary Renal-Cell Carcinoma. *N Engl J Med* 2016; **374**(2): 135-45.
25. Yang Y, Valera V, Sourbier C, et al. A novel fumarate hydratase-deficient HLRCC kidney cancer cell line, UOK268: a model of the Warburg effect in cancer. *Cancer Genet* 2012; **205**(7-8): 377-90.
26. Yang Y, Lane AN, Ricketts CJ, et al. Metabolic reprogramming for producing energy and reducing power in fumarate hydratase null cells from hereditary leiomyomatosis renal cell carcinoma. *PLoS One* 2013; **8**(8): e72179.
27. Zheng L, MacKenzie ED, Karim SA, et al. Reversed argininosuccinate lyase activity in fumarate hydratase-deficient cancer cells. *Cancer Metab* 2013; **1**(1): 12.
28. Zheng L, Cardaci S, Jerby L, et al. Fumarate induces redox-dependent senescence by modifying glutathione metabolism. *Nat Commun* 2015; **6**: 6001.
29. Linehan WM, Schmidt LS, Crooks DR, et al. The Metabolic Basis of Kidney Cancer. *Cancer Discov* 2019; **9**(8): 1006-21.
30. Isaacs JS, Jung YJ, Mole DR, et al. HIF overexpression correlates with biallelic loss of fumarate hydratase in renal cancer: novel role of fumarate in regulation of HIF stability. *Cancer Cell* 2005; **8**(2): 143-53.
31. Xiao M, Yang H, Xu W, et al. Inhibition of alpha-KG-dependent histone and DNA demethylases by fumarate and succinate that are accumulated in mutations of FH and SDH tumor suppressors. *Genes Dev* 2012; **26**(12): 1326-38.
32. Sullivan LB, Gui DY, Vander Heiden MG. Altered metabolite levels in cancer: implications for tumour biology and cancer therapy. *Nat Rev Cancer* 2016; **16**(11): 680-93.
33. Hoekstra AS, de Graaff MA, Briaire-de Bruijn IH, et al. Inactivation of SDH and FH cause loss of 5hmC and increased H3K9me3 in paraganglioma/pheochromocytoma and smooth muscle tumors. *Oncotarget* 2015; **6**(36): 38777-88.

34. Motzer R, Alekseev B, Rha SY, et al. Lenvatinib plus Pembrolizumab or Everolimus for Advanced Renal Cell Carcinoma. *N Engl J Med* 2021; **384**(14): 1289-300.

## Supplementary Note 3

**This is an investigator-initiated, single-arm, single-centre, open-label, phase 2 trial (ClinicalTrials.gov identifier: NCT05877820).**

**CONSORT 2025 checklist of information to include when reporting a randomised trial***

| Section / Topic | No | CONSORT 2025 checklist item description | Reported on page no. |
|---|---|---|---|
| **Title and abstract** | | | |
| Title and structured abstract | 1a | Identification as a randomised trial | NA |
| | 1b | Structured summary of the trial design, methods, results, and conclusions | Manuscript P1-2 |
| **Open science** | | | |
| Trial registration | 2 | Name of trial registry, identifying number (with URL) and date of registration | Manuscript P11 |
| Protocol and statistical analysis plan | 3 | Where the trial protocol and statistical analysis plan can be accessed | Supplementary Information |
| Data sharing | 4 | Where and how the individual de-identified participant data (including data dictionary), statistical code and any other materials can be accessed | Source Data file |
| Funding and conflicts of interest | 5a | Sources of funding and other support (e.g., supply of drugs), and role of funders in the design, conduct, analysis and reporting of the trial | Manuscript P17 |
| | 5b | Financial and other conflicts of interest of the manuscript authors | No conflict of interest |
| **Introduction** | | | |
| Background and rationale | 6 | Scientific background and rationale | Manuscript P2-3<br>Protocol P4-7 |
| Objectives | 7 | Specific objectives related to benefits and harms | Protocol P4 |
| **Methods** | | | |
| Patient and public involvement | 8 | Details of patient or public involvement in the design, conduct and reporting of the trial | Manuscript P11 |
| Trial design | 9 | Description of trial design including type of trial (e.g., parallel group, crossover), allocation ratio, and framework (e.g., superiority, equivalence, non-inferiority, exploratory) | Manuscript P11-12 |
| Changes to trial protocol | 10 | Important changes to the trial after it commenced including any outcomes or analyses that were not prespecified, with reason | No |
| Trial setting | 11 | Settings (e.g., community, hospital) and locations (e.g., countries, sites) where the trial was conducted | Renji Hospital, Shanghai Jiao Tong University School of Medicine, Shanghai, China |

| | | | |
|---|---|---|---|
| Eligibility criteria | 12a | Eligibility criteria for participants | Protocol P12-14 |
| | 12b | If applicable, eligibility criteria for sites and for individuals delivering the interventions (e.g., surgeons, physiotherapists) | NA |
| Intervention and comparator | 13 | Intervention and comparator with sufficient details to allow replication. If relevant, where additional materials describing the intervention and comparator (e.g., intervention manual) can be accessed | Protocol P15<br>Manuscript P12 |
| Outcomes | 14 | Pre-specified primary and secondary outcomes, including the specific measurement variable (e.g., systolic blood pressure), analysis metric (e.g., change from baseline, final value, time to event), method of aggregation (e.g., median, proportion), and time point for each outcome | Protocol P34<br>Manuscript P13 |
| Harms | 15 | How harms were defined and assessed (e.g., systematically, non-systematically) | Protocol P31 |
| Sample size | 16a | How sample size was determined, including all assumptions supporting the sample size calculation | Manuscript P14 |
| | 16b | Explanation of any interim analyses and stopping guidelines | Manuscript P14 |
| Randomisation: | | | NA |
| Sequence generation | 17a | Who generated the random allocation sequence and the method used | NA |
| | 17b | Type of randomisation and details of any restriction (e.g., stratification, blocking and block size) | NA |
| Allocation concealment mechanism | 18 | Mechanism used to implement the random allocation sequence (e.g., central computer/telephone; sequentially numbered, opaque, sealed containers), describing any steps to conceal the sequence until interventions were assigned | NA |
| Implementation | 19 | Whether the personnel who enrolled and those who assigned participants to the interventions had access to the random allocation sequence | NA |
| Blinding | 20a | Who was blinded after assignment to interventions (e.g., participants, care providers, outcome assessors, data analysts) | NA |
| | 20b | If blinded, how blinding was achieved and description of the similarity of interventions | NA |
| Statistical methods | 21a | Statistical methods used to compare groups for primary and secondary outcomes, including harms | Manuscript P14 |
| | 21b | Definition of who is included in each analysis (e.g., all randomised participants), and in which group | Manuscript P14 |
| | 21c | How missing data were handled in the analysis | NA |

| | | | |
|---|---|---|---|
| | 21d | Methods for any additional analyses (e.g., subgroup and sensitivity analyses), distinguishing prespecified from post-hoc | NA |
| **Results** | | | |
| Participant flow, including flow diagram | 22a | For each group, the numbers of participants who were randomly assigned, received intended intervention, and were analysed for the primary outcome | Figure 1 |
| | 22b | For each group, losses and exclusions after randomisation, together with reasons | Figure 1 |
| Recruitment | 23a | Dates defining the periods of recruitment and follow-up for outcomes of benefits and harms | Manuscript P3 |
| | 23b | If relevant, why the trial ended or was stopped | Primary endpoint met |
| Intervention and comparator delivery | 24a | Intervention and comparator as they were actually administered (e.g., where appropriate, who delivered the intervention/comparator, how participants adhered, whether they were delivered as intended [fidelity]) | Manuscript P3-7 |
| | 24b | Concomitant care received during the trial for each group | NA |
| Baseline data | 25 | A table showing baseline demographic and clinical characteristics for each group | Table 1 |
| Numbers analysed, outcomes and estimation | 26 | For each primary and secondary outcome, by group:<br>● the number of participants included in the analysis<br>● the number of participants with available data at the outcome time point<br>● result for each group, and the estimated effect size and its precision (such as 95% confidence interval)<br>● for binary outcomes, presentation of both absolute and relative effect size | Manuscript P3-7 |
| Harms | 27 | All harms or unintended events in each group | Manuscript P5 |
| Ancillary analyses | 28 | Any other analyses performed, including subgroup and sensitivity analyses, distinguishing pre-specified from post-hoc | Manuscript P6-7 |
| **Discussion** | | | |
| Interpretation | 29 | Interpretation consistent with results, balancing benefits and harms, and considering other relevant evidence | Manuscript P7-11 |
| Limitations | 30 | Trial limitations, addressing sources of potential bias, imprecision, generalisability, and, if relevant, multiplicity of analyses | Manuscript P7-11 |

*We strongly recommend reading this statement in conjunction with the CONSORT 2025 Explanation and Elaboration and/or the CONSORT 2025 Expanded Checklist for important clarifications on all the items. We also recommend reading relevant CONSORT extensions. See www.consort-spirit.org.

Citation: Hopewell S, Chan AW, Collins GS, Hróbjartsson A, Moher D, Schulz KF, et al. CONSORT 2025 Statement: updated guideline for reporting randomised trials. BMJ. 2025; 388:e081123. https://dx.doi.org/10.1136/bmj-2024-081123.

© 2025 Hopewell et al. This is an Open Access article distributed under the terms of the Creative Commons Attribution License (https://creativecommons.org/licenses/by/4.0/), which permits unrestricted use, distribution, and reproduction in any medium, provided the original work is properly cited.
