# Supplementary material for: Tislelizumab plus lenvatinib in advanced fumarate hydratase-deficient renal cell carcinoma: a single-arm, phase 2 trial with metabolic biomarker analysis
Source: Nat Commun. 2026 Sep 7;17:10591. doi: 10.1038/s41467-026-77615-9 (PMC13645792; doi:10.1038/s41467-026-77615-9)
Supplement: Supplementary file 2 [file 41467_2026_77615_MOESM2_ESM.pdf]

Description of Additional Supplementary Files

Supplementary Dataset 1: Serial representative images for each patient at baseline, maximal tumor response, and confirmed progression.
