# Supplementary material for: Tislelizumab plus lenvatinib in advanced fumarate hydratase-deficient renal cell carcinoma: a single-arm, phase 2 trial with metabolic biomarker analysis
Source: Nat Commun. 2026 Sep 7;17:10591. doi: 10.1038/s41467-026-77615-9 (PMC13645792; doi:10.1038/s41467-026-77615-9)
Supplement: Supplementary file 3 [file 41467_2026_77615_MOESM4_ESM.pdf]

nature portfolio

Peer Review File

Tislelizumab plus lenvatinib in advanced fumarate hydratase-deficient renal cell carcinoma: a single-arm, phase 2 trial with metabolic biomarker analysis

Corresponding Author: Dr Jin Zhang

This file contains all reviewer reports in order by version, followed by all author rebuttals in order by version.

Version 0:

Reviewer comments:

Reviewer #1

(Remarks to the Author)
This single-arm phase 2 trial evaluated tislelizumab plus lenvatinib as first-line therapy in 20 patients with advanced fumarate hydratase–deficient renal cell carcinoma (FH-RCC), a rare and aggressive kidney cancer lacking a standard treatment. The combination achieved a 95% objective response rate, including 20% complete responses, with a median progression-free survival of 20.7 months and an estimated 2-year overall survival of 89.7%. Toxicities were consistent with known immune checkpoint inhibitor and tyrosine kinase inhibitor combinations and were generally manageable. Exploratory analyses showed that circulating succinate-related metabolites correlated with tumor burden and may serve as early biomarkers of treatment response.

Comments for authors' consideration

1. The study employs a Simon two-stage design; however, the authors should justify why this design was selected over a BOIN/BOP2 design, which may offer greater flexibility and efficiency in early-phase trials.

2. There is inconsistency in the use of spelled-out versus numeric values (e.g., “four achieving CR and 15 showing PR” on page 7). Please standardize number formatting throughout the manuscript.

3. In Table 1, “residual kidney recurrence” should be capitalized consistently (e.g., “Residual kidney recurrence”) to match formatting conventions used elsewhere in the table.

4. The gridlines in the Kaplan–Meier plots reduce visual clarity and should be removed. Additionally, the median PFS is explicitly reported in the PFS figure, whereas the median OS is not reported in the OS figure; please ensure consistent reporting across survival endpoints.

5. The color coding in Figure 2B is not clearly labeled. Please add a legend specifying what each color represents.

6. In the OS curve, dashed lines indicate both the 1.5-year and 2-year OS rates, which appear to be identical. If the values are indeed the same, consider reporting only the 2-year OS rate to avoid redundancy and improve clarity.

Reviewer #2

(Remarks to the Author)
This manuscript reports results from a single-arm, single-center, phase 2 trial (NCT05877820) of tislelizumab plus lenvatinib in 20 treatment-naïve patients with advanced FH-RCC. The authors report a striking 95% ORR (4 CR, 15 PR), median PFS of 20.7 months, and a 2-year OS of 89.7%. An exploratory biomarker analysis demonstrates that circulating succinyl-adenosine and succinic-cysteine track with radiographic tumor burden in most patients. The authors conclude this regimen shows "favorable antitumor activity" and that the metabolites may serve as real-time monitoring biomarkers. I commend the authors for conducting a prospective trial in this exceptionally rare disease, where evidence generation is inherently difficult.

FH-RCC is an area of genuine unmet need, and any prospective data are welcome. The metabolic biomarker work is scientifically interesting. Below are my specific comments:

1. The ORR of 95% is implausibly high and demands greater scrutiny than the authors provide. A 95% ORR in any advanced solid tumor treated with an ICI/TKI combination would be extraordinary. For context, the KEYNOTE-B61 study of the same TKI backbone (lenvatinib) plus pembrolizumab yielded a 49% ORR across non-clear cell RCC (PMID: 37451291). The authors' own prior retrospective ICI/TKI data showed a 27% ORR in FH-RCC (PMID: 35715365). The recently published sintilimab plus axitinib study in a comparable FH-RCC population reported an ORR of 56% (PMID: 40810951). Even the highly selected, NCI-run trial study of bevacizumab/erlotinib in HLRCC-associated RCC reported ORR = 72% (PMID: 40532152). A jump from these figures to 95% requires more than a brief mention in the discussion. Several explanations are possible and should be addressed systematically as outlined below.

2. Patient selection bias: This is a single-center study at a major referral center that has published extensively on FH-RCC. The screened-to-enrolled ratio (25:20) is narrow, but we have no data on the broader referral funnel. Were patients with rapidly progressive or high-volume disease less likely to be referred or enrolled? The fact that 60% had ECOG 0 and 15% were favorable IMDC risk raises this concern as this is a rather high proportions for a cancer characterized by aggressive biology.

3. Tumor response was assessed by two radiologists at the treating institution. In an open-label, single-arm trial, investigator-assessed ORR is known to be inflated relative to blinded independent central review (BICR). The absence of BICR is a critical limitation that the authors understate. Given the headline-grabbing nature of a 95% ORR, BICR should have been incorporated into the study design or at minimum performed post hoc.

4. Lead-time and length-time biases: The median interval from prior nephrectomy to enrollment was 10.7 months, suggesting some patients had relatively indolent recurrence courses. Were any of these patients truly in need of immediate systemic therapy, or could some have been observed? This relates to the broader question of whether the trial population is representative of the typical advanced FH-RCC patient.

5. The authors do not discuss the possibility that the high ORR is partly attributable to the regimen's TKI component alone. Lenvatinib monotherapy achieves meaningful response rates in RCC. Without a comparator arm or any attempt to disentangle the ICI and TKI contributions, attributing the efficacy to the combination is speculative. This is especially relevant given FH-RCC's documented high vascularity and VEGF-pathway dependence.

6. The authors report that germline FH mutation carriers had significantly better PFS (24.8 vs. 8.7 months, p=0.0214) and OS (p=0.0109) compared to patients with somatic biallelic alterations. With only 5 patients in the somatic subgroup, these p-values are unreliable and highly sensitive to individual patient outcomes. The confidence intervals confirm this — the HR for OS is reported as 0.01 (95% CI: 0.00–0.38), which is an essentially meaningless point estimate driven by the small denominator. Reporting formal p-values for a comparison of 15 vs. 5 patients as if this constitutes evidence of a biological difference is misleading. This analysis should be clearly labeled as hypothesis-generating only, and the p-values should be de-emphasized or removed. The possibility that this simply reflects different disease biology (somatic FH-RCC may represent a fundamentally different entity) or unmeasured confounders should be discussed more thoroughly.

7. The exploratory biomarker analysis is the most novel aspect of this manuscript, yet it is presented largely descriptively. Four of 20 patients (20%) had biomarker levels below the detection cutoff and were excluded. This is a substantial attrition rate for a biomarker intended for clinical monitoring and limits the generalizability of the findings.

8. The claim that biomarker changes "preceded" imaging changes is based on visual inspection of individual patient plots (Figure 5), not on any formal statistical framework. No concordance statistics, no time-dependent sensitivity/specificity analysis, and no landmark analysis are provided. What is the lead time? How often do biomarker rises occur without subsequent imaging progression (false positives)? How often does imaging progression occur without biomarker elevation (false negatives)? These are the questions that can determine clinical utility. The claim that these biomarkers could provide "a critical window for treatment modification" is premature without any evidence that acting on biomarker changes improves outcomes.

9. The confounding effect of uterine leiomyomas in female patients is acknowledged but not adequately addressed. If the biomarkers are unreliable in a defined subset of HLRCC patients, this is a significant limitation of their clinical applicability.

10. PD-L1 expression status is not reported for this cohort, despite the discussion invoking tumor immunogenicity as a mechanism. Was PD-L1 testing performed? If so, report it. If not, acknowledge this gap.

Reviewer #3

(Remarks to the Author)

Summary:
This is a prospective phase II study tislelizumab plus lenvatinib for patients with FH deficient metastatic renal cell carcinoma. The study reached planned accrual of 20 patients. The combination achieved the primary endpoint with a

reported ORR of 95%, greatly exceeding the level of interest of 60% pre-specified. All patients were reported to have disease control. While the follow up is relatively short, the PFS and OS are likely unstable though appear impressive. The authors reported the use two primary circulating metabolites and correlation of response and progression. These were pre-specified and were impressive results.

This is the second reported IO/TKI combination for FH-RCC building off a recently published single arm study of axitinib plus sintilimab. The activity is at minimum in line with this previously published data and adds a second study supporting the use of IO/TKI for this rare tumor subtype.

The work supports the conclusions or claims though would recommend that the authors refrain from stating their combination appears more impressive than axitinib plus sintlimab given the small, single arm nature of both studies and difficulties in cross trial comparisons.

The methodology appears sound, though would like the authors to comment on below questions.

I would recommend revision of the manuscript.
Introduction: would cite in line 87 the previous work supporting the metabolites of interest.

Methods in line 122: where there rules about re-escalation in terms of resolution to grade 1 or limit of grade that would allow future re-escalation?

In methods would discuss how follow up was calculated, would suggest reverse Kaplan Meier technique.

In sample size calculation in the previous retrospective series IO/TKI had an ORR of approximately 60%, the previous study with bevacizumab plus erlotinib had an ORR of 72%. Please explain how the authors assumed a response rate of 20-30% to calculate sample size? If using historical data with single agent TKI this would be reasonable to consider.

Results:
Line 198-199 would state length of SD (assume greater than 24 weeks) given 100% DCR
Line 223: the rate of grade 3 adverse events is much lower than previous studies with lenvatinib 20mg, would recommend reporting all adverse events and then treatment related adverse events.
Line 234: what proportion of patients had re-escalation of lenvatinib as allowed in protocol?
Is there any information about dose density? How many breaks occurred per patient, what was the average dosing of patients treatment?
Line 236: what were the immune related adverse events that require high dose steroids? What event led to treatment discontinuation of the immunotherapy?

Discussion
Line 273 would move up the pneumonitis to the results section.
Line 277: add citation
Line 288: Would recommend moving the discussion of the JAMA Oncology manuscript of axitinib plus sintilimab in FH_RCC doi:10.1001/jamaoncol.2025.2497, and discuss similarities of findings to this area of the discussionL
Line 289 histopathology evaluation was part of pre-planned correlative analysis this needs to move to results
Line 294 this should move to the introduction to discuss how you decided about pre-specified IO/TKI response rate. This appears very different than a different retrospective analysis. Liang, J., Sun, G., Pan, X. et al. Genomic and transcriptomic features between primary and paired metastatic fumarate hydratase–deficient renal cell carcinoma. Genome Med 15, 31 (2023). https://doi.org/10.1186/s13073-023-01182-7 which reported much higher IO/TKI responses. Any reason to believe the response to bevacizumab plus erlotinib would be so much lower in the cited article as compared to the prospective study? Were more patients somatic biallelic loss?
Line 303 would remove cross trial declaration of numerical superiority of your combination, given the small sample size and the wide overlapping confidence interval
Line 330 strong part of discussion. Would comment on differences detected between germline and somatic biallelic alteration of circulating markers. If no difference would comment.

Table 3: Would add a line for total % of patients with AE and TRAE

Figure 2: the patient with SD is much lower than 30% cut line, why was this patient SD, was this initial response or best response?

Version 1:

Reviewer comments:

Reviewer #1

(Remarks to the Author)
I appreciate the authors' careful and thorough responses to the previous reviewers' comments. The revised manuscript has been substantially improved.

From a statistical perspective, the authors have adequately addressed the major concerns regarding the study design, efficacy analyses, subgroup analyses, and interpretation of the exploratory biomarker findings. The limitations inherent to this single-arm, single-center phase II study are now appropriately acknowledged, and the conclusions are presented with suitable caution.

The statistical methodology is appropriate for the objectives of this study, and the analyses support the conclusions drawn by the authors. I have no further statistical concerns.

Reviewer #2

(Remarks to the Author)
The authors have addressed my comments.

Reviewer #3

(Remarks to the Author)
All of my concerns in the initial review have been addressed

Open Access This Peer Review File is licensed under a Creative Commons Attribution 4.0 International License, which permits use, sharing, adaptation, distribution and reproduction in any medium or format, as long as you give appropriate credit to the original author(s) and the source, provide a link to the Creative Commons license, and indicate if changes were made.
In cases where reviewers are anonymous, credit should be given to 'Anonymous Referee' and the source.
The images or other third party material in this Peer Review File are included in the article's Creative Commons license, unless indicated otherwise in a credit line to the material. If material is not included in the article's Creative Commons license and your intended use is not permitted by statutory regulation or exceeds the permitted use, you will need to obtain permission directly from the copyright holder.
To view a copy of this license, visit https://creativecommons.org/licenses/by/4.0/

Response to Reviewers' Comments

Reviewer #1 (Remarks to the Author):

This single-arm phase 2 trial evaluated tislelizumab plus lenvatinib as first-line therapy in 20 patients with advanced fumarate hydratase–deficient renal cell carcinoma (FH-RCC), a rare and aggressive kidney cancer lacking a standard treatment. The combination achieved a 95% objective response rate, including 20% complete responses, with a median progression-free survival of 20.7 months and an estimated 2-year overall survival of 89.7%. Toxicities were consistent with known immune checkpoint inhibitor and tyrosine kinase inhibitor combinations and were generally manageable. Exploratory analyses showed that circulating succinate-related metabolites correlated with tumor burden and may serve as early biomarkers of treatment response.

Comments for authors' consideration

1. The study employs a Simon two-stage design; however, the authors should justify why this design was selected over a BOIN/BOP2 design, which may offer greater flexibility and efficiency in early-phase trials.

Thank you for this important comment. We agree that BOIN/BOP2 designs can provide greater flexibility and efficiency in early-phase trials, particularly for dose finding, dose optimization, or adaptive selection among multiple dose levels or regimens.

However, our study was not a dose-finding or regimen-selection trial. It was a single-arm phase 2 study evaluating one pre-specified fixed-dose combination regimen, tislelizumab plus lenvatinib. The primary endpoint was ORR, and the objective was to determine whether this fixed regimen achieved a clinically meaningful response rate compared with a predefined historical benchmark. Therefore, Simon's minimax two-stage design was considered appropriate, as it directly evaluates a binary efficacy endpoint and allows early stopping for futility, which is particularly important in an ultra-rare disease setting.

In addition, two single-arm phase 2 oncology trials recently published in ***Nature Communications*** have also employed Simon's minimax two-stage design with ORR as the primary endpoint, including the study of dalpiciclib plus pyrotinib in HER2-positive advanced breast cancer (***Nat Commun.*** 2023 Oct 7;14(1):6272. PMID: 37805496) and the APICAL-GC trial of anlotinib plus

toripalimab in advanced gastric cancer patients with performance status 2 (*Nat Commun*. 2025 May 31;16(1):5069. PMID: 40450051).

Based on your suggestion, we have carefully revised the protocol Sample Size section and the manuscript Statistical analysis section to clarify the rationale for using Simon' s minimax two-stage design.

2. There is inconsistency in the use of spelled-out versus numeric values (e.g., "four achieving CR and 15 showing PR" on page 7). Please standardize number formatting throughout the manuscript.

We thank the reviewer for this suggestion. We have now unified the numerical format throughout the manuscript to use Arabic numerals for all numerical values in this context (e.g., '4 (20%) achieving CR and 15 (75%) showing PR') to ensure consistency.

3. In Table 1, "residual kidney recurrence" should be capitalized consistently (e.g., "Residual kidney recurrence") to match formatting conventions used elsewhere in the table.

We thank the reviewer for this careful observation. As suggested, we have revised Table 1 by capitalizing "residual kidney recurrence" to "Residual kidney recurrence" to ensure consistent formatting with other entries in the table. The correction has been highlighted in the revised table.

4. The gridlines in the Kaplan–Meier plots reduce visual clarity and should be removed. Additionally, the median PFS is explicitly reported in the PFS figure, whereas the median OS is not reported in the OS figure; please ensure consistent reporting across survival endpoints.

Thank you for your thoughtful feedback. We have revised the Kaplan–Meier plots accordingly: gridlines have been removed to enhance visual clarity. In addition, we have now explicitly reported the median OS in the OS figure to ensure consistency with the PFS figure. The updated figures have been incorporated into the revised manuscript. We believe these changes improve the transparency and readability of survival data.

5. The color coding in Figure 2B is not clearly labeled. Please add a legend specifying what each color represents.

Thank you for your helpful comment. We have revised Figure 2B by adding a clear legend that specifies what each color represents. The updated figure now includes the legend in an appropriate position, which we believe greatly

improves the interpretability of the figure. We appreciate your careful reading and suggestion.

6. In the OS curve, dashed lines indicate both the 1.5-year and 2-year OS rates, which appear to be identical. If the values are indeed the same, consider reporting only the 2-year OS rate to avoid redundancy and improve clarity.

Thank you for your careful observation. We confirm that the 1.5-year and 2-year OS rates were indeed identical. Following your suggestion, we have revised the OS KM curve by removing the dashed line and label for the 1.5-year rate and now report only the 2-year OS rate. This change eliminates redundancy and improves the clarity of the figure. We appreciate your constructive input.

Reviewer #2 (Remarks to the Author):

This manuscript reports results from a single-arm, single-center, phase 2 trial (NCT05877820) of tislelizumab plus lenvatinib in 20 treatment-naïve patients with advanced FH-RCC. The authors report a striking 95% ORR (4 CR, 15 PR), median PFS of 20.7 months, and a 2-year OS of 89.7%. An exploratory biomarker analysis demonstrates that circulating succinyl-adenosine and succinic-cysteine track with radiographic tumor burden in most patients. The authors conclude this regimen shows ″favorable antitumor activity″ and that the metabolites may serve as real-time monitoring biomarkers. I commend the authors for conducting a prospective trial in this exceptionally rare disease, where evidence generation is inherently difficult. FH-RCC is an area of genuine unmet need, and any prospective data are welcome. The metabolic biomarker work is scientifically interesting. Below are my specific comments:

1. The ORR of 95% is implausibly high and demands greater scrutiny than the authors provide. A 95% ORR in any advanced solid tumor treated with an ICI/TKI combination would be extraordinary. For context, the KEYNOTE-B61 study of the same TKI backbone (lenvatinib) plus pembrolizumab yielded a 49% ORR across non-clear cell RCC (PMID: 37451291). The authors' own prior retrospective ICI/TKI data showed a 27% ORR in FH-RCC (PMID: 35715365). The recently published sintilimab plus axitinib study in a comparable FH-RCC population reported an ORR of 56% (PMID: 40810951). Even the highly selected, NCI-run trial study of bevacizumab/erlotinib in HLRCC-associated RCC reported ORR = 72% (PMID: 40532152). A jump from these figures to 95% requires more than a brief mention in the discussion. Several explanations are possible

and should be addressed systematically as outlined below.

Thank you for your thoughtful and rigorous review. We fully understand your concern regarding the unusually high ORR of 95% observed in our study, especially when compared with the published data you kindly cited (KEYNOTE-B61: 49%; our own prior retrospective study: 27%; sintilimab plus axitinib: 56%; and the NCI bevacizumab/erlotinib trial: 72%). We agree that such a high ORR is extraordinary and deserves more than a brief discussion. In response to your comment, we have now included in the supplementary materials a comprehensive set of pretreatment and posttreatment imaging for all enrolled patients. These images have been independently reviewed and clearly demonstrate the objective responses that form the basis of our ORR calculation. In the revised Discussion, we have added dedicated paragraphs analyzing the possible reasons for the high ORR, including the small sample size, the molecular nature of FH-RCC sensitive to ICI/TKI combinations, the different TKI agents among related studies, and the single-arm, open-label design which could have overestimated the true effect size. Accordingly, we no longer merely mention the high ORR briefly; instead, we critically examine it in light of all the studies you pointed out, and we emphasize that confirmatory studies with larger sample sizes are necessary before any definitive conclusion can be drawn. We believe the revised manuscript now presents these findings with appropriate caution and transparency.

We thank you again for your incisive and constructive critique, which has substantially improved the quality of our paper.

2. Patient selection bias: This is a single-center study at a major referral center that has published extensively on FH-RCC. The screened-to-enrolled ratio (25:20) is narrow, but we have no data on the broader referral funnel. Were patients with rapidly progressive or high-volume disease less likely to be referred or enrolled? The fact that 60% had ECOG 0 and 15% were favorable IMDC risk raises this concern as this is a rather high proportions for a cancer characterized by aggressive biology.

We thank the reviewer for raising this important concern regarding potential selection bias in a single-center study. We fully acknowledge that our cohort showed a relatively high proportion of ECOG 0 (60%) and favorable IMDC risk (15%), which may raise concerns about generalizability. We do not claim that our cohort is representative of all FH-RCC patients in the real world, and we agree that we lack data on the broader referral funnel (i.e., patients

who were never referred to our center). Nevertheless, we would like to provide the following information to help contextualize our findings:

First, although this is a single-center study, our enrolled patients came from 10 different provinces/municipalities across China (Shanghai: 1; Jiangsu: 5; Zhejiang: 3; Jiangxi: 2; Fujian: 1; Liaoning: 3; Shandong: 2; Hainan: 1; Anhui: 2). This broad geographic distribution suggests that our findings are not limited to a single local population.

Second, we compared our baseline characteristics with those of two other published trials in advanced FH-RCC or related histologies. In the bevacizumab + erlotinib trial (*N Engl J Med.* 2025 Jun 19;392(23):2346-2356. PMID: 40532152.) (HLRCC-associated papillary RCC, n=43), ECOG 0 was 67% and favorable IMDC risk 26%. In the sintilimab + axitinib trial (*JAMA Oncol.* 2025 Oct 1;11(10):1169-1177. PMID: 40810951) (advanced FH-RCC), favorable IMDC risk was 7% (Figure R1). Our cohort's baseline characteristics fall within the range of these published trial populations, suggesting that our results are broadly comparable to those from other clinical trial settings — not obviously skewed toward a uniquely favorable subgroup.

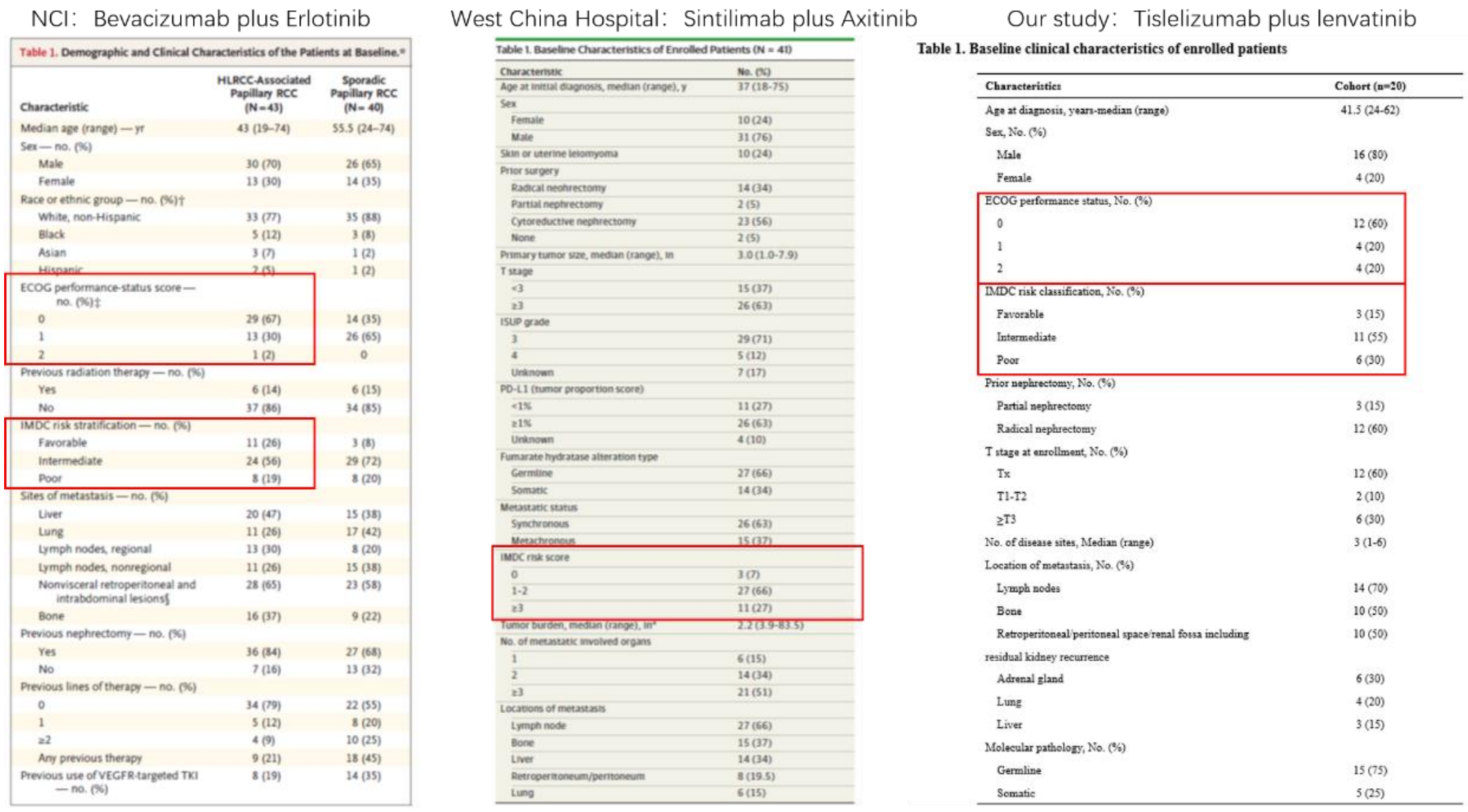

NCI: Bevacizumab plus Erlotinib

Table 1. Demographic and Clinical Characteristics of the Patients at Baseline.*

Characteristic	HLRCC-Associated Papillary RCC (N=43)	Sporadic Papillary RCC (N=40)
Median age (range) — yr	43 (19–74)	55.5 (24–74)
Sex — no. (%)		
Male	30 (70)	26 (65)
Female	13 (30)	14 (35)
Race or ethnic group — no. (%)†		
White, non-Hispanic	33 (77)	35 (88)
Black	5 (12)	3 (8)
Asian	3 (7)	1 (2)
Hispanic	2 (5)	1 (2)
ECOG performance-status score — no. (%)‡		
0	29 (67)	14 (35)
1	13 (30)	26 (65)
2	1 (2)	0
Previous radiation therapy — no. (%)		
Yes	6 (14)	6 (15)
No	37 (86)	34 (85)
IMDC risk stratification — no. (%)		
Favorable	11 (26)	3 (8)
Intermediate	24 (56)	29 (72)
Poor	8 (19)	8 (20)
Sites of metastasis — no. (%)		
Liver	20 (47)	15 (38)
Lung	11 (26)	17 (42)
Lymph nodes, regional	13 (30)	8 (20)
Lymph nodes, nonregional	11 (26)	15 (38)
Nonvisceral retroperitoneal and intrabdominal lesions§	28 (65)	23 (58)
Bone	16 (37)	9 (22)
Previous nephrectomy — no. (%)		
Yes	36 (84)	27 (68)
No	7 (16)	13 (32)
Previous lines of therapy — no. (%)		
0	34 (79)	22 (55)
1	5 (12)	8 (20)
≥2	4 (9)	10 (25)
Any previous therapy	9 (21)	18 (45)
Previous use of VEGFR-targeted TKI — no. (%)	8 (19)	14 (35)

West China Hospital: Sintilimab plus Axitinib

Table 1. Baseline Characteristics of Enrolled Patients (N = 41)

Characteristic	No. (%)
Age at initial diagnosis, median (range), y	37 (18-75)
Sex	
Female	10 (24)
Male	31 (76)
Skin or uterine leiomyoma	10 (24)
Prior surgery	
Radical neohrectomy	14 (34)
Partial nephrectomy	2 (5)
Cytoreductive nephrectomy	23 (56)
None	2 (5)
Primary tumor size, median (range), in	3.0 (1.0-7.9)
T stage	
<3	15 (37)
≥3	26 (63)
ISUP grade	
3	29 (71)
4	5 (12)
Unknown	7 (17)
PD-L1 (tumor proportion score)	
<1%	11 (27)
≥1%	26 (63)
Unknown	4 (10)
Fumarate hydratase alteration type	
Germline	27 (66)
Somatic	14 (34)
Metastatic status	
Synchronous	26 (63)
Metachronous	15 (37)
IMDC risk score	
0	3 (7)
1-2	27 (66)
≥3	11 (27)
Tumor burden, median (range), in²	2.2 (3.9-83.5)
No. of metastatic involved organs	
1	6 (15)
2	14 (34)
≥3	21 (51)
Locations of metastasis	
Lymph node	27 (66)
Bone	15 (37)
Liver	14 (34)
Retroperitoneum/peritoneum	8 (19.5)
Lung	6 (15)

Our study: Tislelizumab plus lenvatinib

Table 1. Baseline clinical characteristics of enrolled patients

Characteristics	Cohort (n=20)
Age at diagnosis, years-median (range)	41.5 (24-62)
Sex, No. (%)	
Male	16 (80)
Female	4 (20)
ECOG performance status, No. (%)	
0	12 (60)
1	4 (20)
2	4 (20)
IMDC risk classification, No. (%)	
Favorable	3 (15)
Intermediate	11 (55)
Poor	6 (30)
Prior nephrectomy, No. (%)	
Partial nephrectomy	3 (15)
Radical nephrectomy	12 (60)
T stage at enrollment, No. (%)	
Tx	12 (60)
T1-T2	2 (10)
≥T3	6 (30)
No. of disease sites, Median (range)	3 (1-6)
Location of metastasis, No. (%)	
Lymph nodes	14 (70)
Bone	10 (50)
Retroperitoneal/peritoneal space/renal fossa including	10 (50)
residual kidney recurrence	
Adrenal gland	6 (30)
Lung	4 (20)
Liver	3 (15)
Molecular pathology, No. (%)	
Germline	15 (75)
Somatic	5 (25)

Figure R1. Comparison of baseline characteristics among similar studies related to FH-RCC.

Third, to improve transparency, we have now included in the revised manuscript representative imaging panels (Supplemental Figure Panel S3) for each patient at baseline, maximum response, and confirmed progression, as well as a supplementary table (Supplemental Table S1) detailing individual baseline

tumor burden.

Overall, we acknowledge that the lack of data on the broader referral funnel might preclude definitive conclusions about selection bias, but our cohort's baseline characteristics seemed broadly comparable to those from other clinical trial settings and, to some extent, could represent most FH-RCC patients in the real-world circumstances.

We thank the reviewer for helping us improve the transparency and rigor of our manuscript.

3. Tumor response was assessed by two radiologists at the treating institution. In an open-label, single-arm trial, investigator-assessed ORR is known to be inflated relative to blinded independent central review (BICR). The absence of BICR is a critical limitation that the authors understate. Given the headline-grabbing nature of a 95% ORR, BICR should have been incorporated into the study design or at minimum performed post hoc.

We agree that blinded independent central review (BICR) is an important strategy to reduce potential bias, particularly in open-label, single-arm trials.

Previous studies reported discrepancies between BICR and local investigator assessments for PFS (***Eur J Cancer.*** 2024 Dec;213:115077. PMID: 39454538). However, more recent evidence suggests that for ORR, the primary endpoint of our study, no meaningful differences are observed between BICR and investigator assessments (***J Clin Oncol*** 2024 42, e23000-e23000; ***Cancers (Basel).*** 2025 Mar 25;17(7):1096. PMID: 40227586). Meta-analysis also indicates a high degree of concordance between BICR and local evaluation of ORR and PFS across many oncology trials (***Oncologist.*** 2024 Aug 5;29(8):e1073-e1081. PMID: 36905580). We note that investigator-assessed response based on RECIST 1.1 remains a widely accepted and standardized approach for efficacy evaluation in recent oncology trials (***Lancet Oncol.*** 2025 Oct;26(10):1289-1299. PMID: 40915303), especially in rare tumor settings where feasibility constraints are substantial.

This study focuses on FH-RCC, a rare and aggressive malignancy with inherently limited patient numbers and restricted opportunities for prospective investigation. In this context, early-phase, single-arm studies using RECIST-based investigator assessment represent a pragmatic and commonly adopted approach for timely evaluation of treatment activity.

However, to further reduce potential assessment bias in this open-label, single-arm study, a post-hoc independent radiological review was performed. All available baseline and post-baseline imaging data were anonymized and reviewed by two independent radiologists who were not involved in patient care or the original response assessment. Tumor response was reassessed according to RECIST 1.1. Discrepancies were resolved by consensus or by adjudication from a third independent radiologist. Finally, the post-hoc independent radiological assessment supported the investigator-reported benefits in both ORR and PFS (Supplemental Table S3).

Furthermore, we have now included in the revised manuscript representative imaging panels (Supplemental Figure Panel S3) for each patient at baseline, maximum response, and confirmed progression to improve transparency and reproducibility.

Finally, we thank the reviewer for raising this important point, and we have revised the manuscript to explicitly acknowledge the absence of BICR as a limitation and to better contextualize the interpretation of the efficacy results.

4. Lead-time and length-time biases: The median interval from prior nephrectomy to enrollment was 10.7 months, suggesting some patients had relatively indolent recurrence courses. Were any of these patients truly in need of immediate systemic therapy, or could some have been observed? This relates to the broader question of whether the trial population is representative of the typical advanced FH-RCC patient.

Thank you for your thoughtful and rigorous comments. We completely agree with you that FH-RCC is a highly heterogeneous disease, and both lead-time bias and length-time bias are legitimate concerns when interpreting trial outcomes in post-nephrectomy recurrence settings. We appreciate you pushing us to examine these issues more deeply.

First，we acknowledge the heterogeneity and potential biases as inherent limitations. FH-RCC can exhibit a wide spectrum of clinical behavior—from indolent recurrences months or even years after nephrectomy to fulminant, rapidly progressive disease. Our single-arm trial, by design, cannot fully eliminate the possibility that some patients with slowly progressing disease might have been included. We also acknowledge that the absence of a prospective control arm receiving observation alone limits our ability to definitively quantify the net benefit of systemic therapy across all disease

phenotypes.

However, our detailed case-level analysis confirms that no patient was inappropriately enrolled. Despite this heterogeneity, we performed a granular stratification of all 20 enrolled patients. The key findings are listed below:

(1) Five patients presented with de novo metastatic disease at initial diagnosis ($T_{1-4}N_{0-1}M_1$), immediate systemic therapy is guideline-indicated, and no upfront nephrectomy was performed in these patients.

(2) Four patients had surgery-to-enrollment interval <3 months, including 2 cases receiving cytoreductive nephrectomy when diagnosed as advanced-stage disease (P03, P06), and 1 case of bilateral renal tumors plus retroperitoneal lymph nodes metastasis with cytoreductive partial nephrectomy of one kidney and leaving the contralateral tumor and lymph nodes untreated (P02), and 1 case of early local recurrence with positive margin in the initial partial nephrectomy (P08) , none of which were suitable for observation.

(3) The remaining 11 patients were initially diagnosed as non-metastatic disease ($T_{1-3}N_{0-1}M_0$) at nephrectomy. Their median interval from radical nephrectomy to enrollment was 13.7 months. Critically, every single patient in this subgroup had either symptomatic progression, evident RECIST-defined progression during the lead-in period, or high-volume disease threatening vital organs prior to enrollment. None had stable disease for >6 months.

Thus, while FH-RCC is heterogeneous, within this specific trial cohort, all patients met clear, guideline-consistent criteria for immediate systemic therapy. No patient who could have been safely observed was enrolled.

Furthermore, comparison with real-world data supports representativeness. In our previously published multicenter real-world retrospective study (***Eur Urol.*** 2023 Feb;83(2):163-172.PMID: 35715365), the median recurrence-free survival after nephrectomy was 9.0 months (95% CI 5.5–12.5) within the subgroup of 54 FH-RCC patients with initially non-metastatic disease. Both the median interval from prior nephrectomy to enrollment for the overall trial population (10.7 months) and the median interval for the subgroup of patients who underwent definitive nephrectomy for initially non-metastatic disease (13.7 months) are close to the real-world retrospective data. The similarity between these time intervals supports that our trial cohort is representative of the typical disease trajectory of advanced FH-RCC requiring systemic therapy.

We thank the reviewer for raising these critical methodologic issues. We have

explicitly acknowledged FH-RCC heterogeneity and the inherent biases as study limitations; provided case-level data demonstrating that no patient in our trial was inappropriate for immediate therapy and proposed specific future directions to address these biases in subsequent studies. We believe these revisions make our manuscript more balanced, transparent, and scientifically rigorous.

5. The authors do not discuss the possibility that the high ORR is partly attributable to the regimen's TKI component alone. Lenvatinib monotherapy achieves meaningful response rates in RCC. Without a comparator arm or any attempt to disentangle the ICI and TKI contributions, attributing the efficacy to the combination is speculative. This is especially relevant given FH-RCC's documented high vascularity and VEGF-pathway dependence.

Thank you for raising this important question regarding attribution of the observed high ORR to the TKI versus ICI component. We fully agree with your points: the VEGF pathway is indeed a key dependency in FH-RCC, lenvatinib monotherapy is likely active, and our single-arm design cannot definitively disentangle the relative contributions of each agent. However, we would like to offer several considerations that support the clinical rationale and value of the combination regimen, without overclaiming superiority.

First, we acknowledge the inherent limitations of single-arm trials in rare tumors. We completely agree that a head-to-head randomized controlled trial would be ideal to address this question. However, for an ultra-rare tumor such as FH-RCC, conducting such a trial is extremely challenging due to slow patient accrual and limited funding. We have added this as a limitation in the Discussion.

Second, ICI/TKI combinations were accepted without TKI-monotherapy arms in the precedent from clear cell RCC. As the reviewer may appreciate, the well-recognized trials for first-line treatment in clear cell RCC, KEYNOTE-426 (pembrolizumab + axitinib vs. sunitinib), CheckMate-9ER (nivolumab + cabozantinib vs. sunitinib), and CLEAR studies (pembrolizumab + lenvatinib vs. lenvatinib + everolimus vs. sunitinib), did not include TKI-monotherapy arms (axitinib, cabozantinib, or lenvatinib alone) comparing directly against the combination, although we know that axitinib, cabozantinib, and lenvatinib monotherapy are all rational options for advanced RCC with superior efficacy compared with sunitinib (*N Engl J Med.* 2019 Mar 21;380(12):1116-1127. PMID: 30779529; *N Engl J Med.* 2021 Mar 4;384(9):829-841.PMID: 33657295; *N Engl J*

Med. 2021 Apr 8;384(14):1289-1300. PMID: 33616314). Nevertheless, these combinations have become standard of care based on superiority over sunitinib. The field has accepted that the benefit derives from the combination, even without isolating the TKI-alone contribution. Similarly, in the phase 2 study of bevacizumab + erlotinib in advanced papillary RCC, the remarkable ORR of 72% in the HLRCC-associated subgroup could not be definitively attributed to either agent alone, yet the regimen is considered active and clinically meaningful (***N Engl J Med.*** 2025 Jun 19;392(23):2346-2356. PMID: 40532152.). Analogously, our study demonstrated that the lenvatinib plus tislelizumab combination had important clinical activity in first-line treatment of advanced FH-RCC.

Furthermore, there is indirect evidence from non-clear cell RCC supporting incremental benefit of adding ICI to TKI. We have searched the literature and found no prospective data on lenvatinib monotherapy in non-clear cell RCC. However, data from other TKIs provide useful context (***Lancet*** 2021;397:695-703; ***J Clin Oncol*** 2021;39:1029-1039; ***J Clin Oncol*** 2022;40:2333-2341; ***Lancet Oncol.*** 2023 Aug;24(8):881-891.) (Table R1):

Table R1. Comparison of response rates among trials using TKI or ICI monotherapy and combinations

Study	Regimen	Population	ORR
SWOG 1500	Cabozantinib monotherapy	Metastatic papillary RCC	23%
KEYNOTE-427 (Cohort B)	Pembrolizumab monotherapy	Metastatic non-clear cell RCC	27%
CA209-9KU	Cabozantinib +nivolumab	Metastatic non-clear cell RCC	47.5%
KEYNOTE-B61	Lenvatinib +pembrolizumab	Metastatic non-clear cell RCC	49%

As shown above, the addition of ICI to TKI approximately doubles the ORR compared to either agent alone. While cross-trial comparisons must be interpreted cautiously, the magnitude of difference strongly suggests a genuine combination effect rather than TKI alone driving the response.

Furthermore, Chen et al. used the IPDfromKM package to reconstruct individual patient data from the Kaplan-Meier curves of SWOG 1500, KEYNOTE-B61, and CA209-9KU studies. Their reconstructed analysis demonstrated that both pembrolizumab + lenvatinib and nivolumab + cabozantinib provided superior PFS and OS compared to cabozantinib monotherapy, further supporting the added value of TKI+ICI combinations in advanced non-clear cell RCC (***Clin Genitourin Cancer.*** 2025 Dec;23(6):102458. PMID: 41193356).

All in all, we thank the reviewer for pushing us to address this fundamental interpretative challenge, which has substantially improved the scientific rigor of our manuscript. We have revised conclusions to be more circumspect, avoiding overstatement.

6. The authors report that germline FH mutation carriers had significantly better PFS (24.8 vs. 8.7 months, p=0.0214) and OS (p=0.0109) compared to patients with somatic biallelic alterations. With only 5 patients in the somatic subgroup, these p-values are unreliable and highly sensitive to individual patient outcomes. The confidence intervals confirm this — the HR for OS is reported as 0.01 (95% CI: 0.00–0.38), which is an essentially meaningless point estimate driven by the small denominator. Reporting formal p-values for a comparison of 15 vs. 5 patients as if this constitutes evidence of a biological difference is misleading. This analysis should be clearly labeled as hypothesis-generating only, and the p-values should be de-emphasized or removed. The possibility that this simply reflects different disease biology (somatic FH-RCC may represent a fundamentally different entity) or unmeasured confounders should be discussed more thoroughly.

Thank you for this critical statistical critique. You are absolutely correct. With only 5 patients in the somatic mutation subgroup, the p-values and hazard ratios we reported were unstable, highly sensitive to individual outcomes, and should not be interpreted as definitive evidence of a biological difference. We fully accept this limitation and have revised the manuscript accordingly.

First, we agree that the small sample size renders formal statistical inference unreliable. As the reviewer correctly notes, a comparison of 15 germline versus 5 somatic mutation patients produces an HR for OS of 0.01 (95% CI 0.00–0.38). This extremely wide confidence interval reflects the instability of the estimate, a single event or censoring event in the somatic group could dramatically change the result. Reporting a formal p-value in

this context is indeed misleading, and we thank the reviewer for pointing this out.

Therefore, we have revised the manuscript to de-emphasize p-values and label the analysis as hypothesis-generating. The following changes have been made: (1) Removed p-values from the abstract and results summary where this comparison appeared; (2) Added a clear disclaimer in the Results section: "Given the small number of patients in the somatic subgroup (n=5), formal statistical comparisons are underpowered and should be interpreted as hypothesis-generating only. The p-values are exploratory and highly sensitive to individual patient outcomes and therefore were not reported"; (3) Acknowledge this limitation in the Discussion and Conclusion section.

7. The exploratory biomarker analysis is the most novel aspect of this manuscript, yet it is presented largely descriptively. Four of 20 patients (20%) had biomarker levels below the detection cutoff and were excluded. This is a substantial attrition rate for a biomarker intended for clinical monitoring and limits the generalizability of the findings.

Thank you for this thoughtful and critical comment. You raise two important points: (1) the biomarker analysis was presented largely descriptively, and (2) the 20% exclusion rate due to undetectable levels was substantial and limits generalizability. We agree with both observations and have substantially revised the manuscript to address them. However, we would like to offer two additional perspectives that we believe are important for balanced interpretation.

First, we agree that our analysis was descriptive and exploratory. We have clearly labeled this analysis as hypothesis-generating in the revised manuscript. As a pre-specified exploratory endpoint, our goal was to generate signals for future research, not to clinically validate a ready-to-use monitoring test. We have focused the presentation on descriptive trends and individual patient trajectories, avoiding over-interpretation.

Second, you are so correct that a 20% exclusion rate was substantial. We have carefully re-examined these four excluded patients and offer the following clarifications.

These patients had very low, not absent, biomarker levels. The four patients did not have "zero" or "absent" levels. Their metabolite concentrations fell below the predefined thresholds (succinic-cysteine 32 ng/mL, succinyl-adenosine 12 ng/mL) - thresholds that were originally established using our

specific mass spectrometry platform and were not biological absolutes. We have clarified this in the Methods section. However, low levels could lead to unreliable measurements. At such low concentrations, multiple factors compromise data quality, including low signal-to-noise ratio; high proportional technical variability; marked instability in serial measurements (Supplementary Figure S2) among which biomarker trajectories oscillate widely without any correlation with tumor burden changes on imaging. Therefore, excluding these patients was a methodological necessity to avoid analyzing unreliable data, not a form of selection bias. We have added this explanation to the Results and Discussion. When we go back to the imaging, this low-level subgroup predominantly had low tumor burden and localized lymph node recurrence. In these patients, the absolute concentration of circulating metabolites likely falls below the current assay's limit of quantification. We have added supplemental materials to characterize this subset of patients (Supplemental Figure S2). So, we explicitly acknowledge that, for patients with biomarker levels above the threshold (80% of our cohort), the assay provides stable, interpretable, and tumor-burden-correlated data; for patients with levels below the threshold (20% of our cohort), the current assay is not reliable and should not be used for clinical monitoring.

Two additional perspectives for balanced interpretation would be significance of biomarkers in kidney cancer and future technical improvements. To our knowledge, there is currently no validated blood-based biomarker for disease monitoring in FH-RCC. In this context, a metabolic marker that provides interpretable, tumor-burden-correlated data in 16 of 20 patients (80%) is, we believe, a non-trivial advance. It offers a potential tool for the majority of patients, where none existed before. We have added the following sentence to the Discussion: "Considering the complete absence of established circulating biomarkers for FH-RCC, the ability to monitor disease dynamics in 80% of patients using this metabolic marker represents a meaningful step forward, even if the current assay requires further refinement for low-level cases."; "The 20% exclusion rate might reflect the current sensitivity limitation of our assay, not a fundamental failure of the biomarker concept. With future technical advances — such as high-resolution mass spectrometry, optimized sample preparation, or more sensitive detection modes — the limit of quantification can be lowered, and the operational thresholds can be revised downward. This would extend detectability to low-tumor-burden patients and reduce the exclusion rate.".

8. The claim that biomarker changes "preceded" imaging changes is based on visual inspection of individual patient plots (Figure 5), not on any formal statistical framework. No concordance statistics, no time-dependent sensitivity/specificity analysis, and no landmark analysis are provided. What is the lead time? How often do biomarker rises occur without subsequent imaging progression (false positives)? How often does imaging progression occur without biomarker elevation (false negatives)? These are the questions that can determine clinical utility. The claim that these biomarkers could provide "a critical window for treatment modification" is premature without any evidence that acting on biomarker changes improves outcomes.

Thank you so much for this rigorous and important methodological critique. You are correct on all counts: our claim that biomarker changes "preceded" imaging changes was based on visual inspection, not on formal statistical analysis; we provided no lead-time, no false-positive/false-negative rates, no landmark or time-dependent ROC analysis; and without such evidence, the statement that these biomarkers could provide "a critical window for treatment modification" is premature. We fully accept these points.

We have revised the manuscript to remove over-interpretation and to state only what the data actually showed. We have made the following specific changes, including deleting of any definitive claim that biomarker changes "preceded" imaging changes; deletion of the statement that the biomarkers could provide "a critical window for treatment modification"; replacing with a strictly descriptive, visual observation: "Visual inspection of serial biomarker and imaging plots suggested that biomarker elevations occurred temporally before radiographic progression. However, our study was unable to provide formal lead time estimates, false-positive or false-negative rates, time-dependent sensitivity or specificity, or landmark analysis due to the small sample size and exploratory nature of this analysis". Furthermore, we added explicit language in the Discussion section stating what was not done (lead-time calculation, false-positive/false-negative rates, landmark analysis) and why these are not possible in this small exploratory study.

However, we respectfully wish to clarify what this exploratory analysis does contribute, without over-claiming. We agree with the reviewer that this analysis did not provide validation for clinical use. But we believe it still has value as a proof-of-concept and hypothesis-generating investigation for the following reasons:

First, to our knowledge, this is the first study to serially measure circulating metabolic biomarkers in FH-RCC patients receiving systemic therapy. We showed that such monitoring was technically feasible.

Second, in 16 of 20 patients (80%), biomarker levels were detectable and showed dynamics that visually paralleled or preceded imaging changes in some cases (Figure 5: P01, P10, P11, P12). For a rare cancer with no established circulating biomarker, detecting any signal at all would be a meaningful first step.

Finally, our descriptive observations provided a rationale for larger, prospective, statistically powered studies to rigorously evaluate key performance parameters, including lead time, false-positive rates, and overall clinical utility.

We revised the Discussion and explicitly stressed the value of this study: "Despite the absence of formal statistical validation, this exploratory analysis provides valuable proof-of-concept evidence for the utility of circulating biomarkers in kidney cancer, a malignancy for which few biomarker has yet been validated in clinical practice. Our study demonstrated that serial metabolic biomarker monitoring was feasible in this rare cancer and that in the majority of patients, biomarker dynamics could be visualized in parallel with radiographic tumor changes, with some cases suggesting a temporal lead. These observations provide a rationale and a signal for future prospective cohort studies designed to test whether biomarker-guided monitoring improves outcomes."

9. The confounding effect of uterine leiomyomas in female patients is acknowledged but not adequately addressed. If the biomarkers are unreliable in a defined subset of HLRCC patients, this is a significant limitation of their clinical applicability.

Thank you for highlighting this important confounding factor. You are correct: the potential interference of uterine leiomyomas in female HLRCC patients is a significant limitation, and our previous acknowledgement of this issue was insufficiently addressed. We have now thoroughly re-analyzed four female patients in our cohort and provide the following detailed analysis and revised interpretation.

We have summarized detailed, case-level, clinical and biomarker data for all four female patients in the table below (Table R2 and Supplemental Figure Panel S4):

Table R2. Details of female participants regarding uterine leiomyomas history.

Patient ID	Uterine Status	Baseline succinic cysteine	Baseline succinyl-adenosine	Observed biomarker behavior
P18	Prior myomectomy	Below threshold	Below threshold	Excluded from main analysis
P17	Prior hysterectomy	Above threshold	Above threshold	Both biomarkers correlated well with tumor burden
P06	Leiomyoma (slow increase)	Above threshold	Above threshold	Succinic-cysteine showed deviation from tumor burden; Succinyl-adenosine remained correlated
P15	Leiomyoma (stable)	Above threshold	Below threshold	Succinyl-adenosine below threshold; Succinic-cysteine decreased with treatment, but magnitude less than tumor burden reduction

Based on these observations, we draw the following possible inference:

(1) No interference when uterus is absent (P17): After hysterectomy, both biomarkers showed clean correlation with tumor burden, suggesting the metabolic signature originates predominantly from tumor tissue, not from extra-uterine sources.

(2) Possible interference from growing leiomyomas (P06): In this patient with multiple leiomyoma untreated, succinic cysteine increased progressively and disproportionately to the responding metastatic disease, coinciding with radiographic slow enlargement of the leiomyoma. Succinyl-adenosine however, remained correlated with tumor burden.

(3) Possible low-level interference from stable leiomyomas (P15): succinic cysteine decreased with treatment but not as steeply as tumor burden, possibly reflecting a persistent low-level signal from the stable leiomyoma. Succinyl-adenosine was undetectable at baseline, possibly due to lower tumor burden or different biochemistry mechanism.

We acknowledge that we cannot quantitatively resolve the impact of leiomyomas.

We fully agree with the reviewer that with only four female patients and limited event numbers, we cannot statistically disentangle the relative contributions of metastatic RCC versus uterine leiomyomas to circulating metabolite levels. We have added a clear statement of this limitation in the revised manuscript: “the small number of female patients and the absence of quantitative metabolite measurements from leiomyoma tissue preclude any definitive assessment of the confounding effect of uterine leiomyomas. Whether these metabolites are produced by leiomyomas, and if so, to what extent, remains unknown.”

However, we argue that this limitation does not negate the clinical utility of the biomarkers for the following reasons. First, at least one biomarker remains informative in most female patients. In P06 (with slowly growing leiomyoma), succinyl-adenosine still tracked tumor burden well, even though succinic cysteine was confounded. In P15 (with stable leiomyoma), succinic cysteine still showed a directional response to treatment, albeit with attenuated magnitude. In P17 (post-hysterectomy), both biomarkers performed as expected. These examples suggest that a dual-metabolite panel offers valuable redundancy: when one marker shows unexpected deviation, the other may remain interpretable, prompting appropriate clinical correlation with imaging to exclude leiomyoma growth. Second, clinical context and imaging can resolve potential ambiguity. In clinical practice, if a female HLRCC patient shows a rising biomarker without corresponding progression of metastatic disease on imaging, the possibility of leiomyoma growth should be considered. Conversely, concordant increases in both biomarkers and imaging findings would support disease progression and warrant therapeutic adjustment. We have therefore emphasized in the Discussion that” The biomarker should not be used in isolation but as an adjunct to clinical and radiographic assessment.” Furthermore, ongoing technical advances may eventually mitigate this issue in the future. Future studies could develop metabolite “fingerprints” capable of distinguishing RCC-derived versus leiomyoma-derived signals, for instance through higher-resolution mass spectrometry to identify isoform-specific patterns. Until such approaches become available, we recommend cautious interpretation of biomarker trends in female patients with known uterine leiomyomas.

Finally, we wish to emphasize that these metabolic biomarkers, at their current stage of development, are intended as a complement to —not a replacement for— standard imaging. We fully acknowledge that several

important questions remain to be addressed, including the confounding effect of uterine leiomyomas, optimal detection thresholds, formal validation of lead time, false-positive and false-negative rates, and, ultimately, whether biomarker-guided monitoring improves clinical outcomes. Rather than claiming to have resolved these challenges, our study provides proof-of-concept evidence along with a rationale for future prospective investigations. We believe this honest and balanced perspective appropriately reflects both the promise and the current limitations of this approach.

10. PD-L1 expression status is not reported for this cohort, despite the discussion invoking tumor immunogenicity as a mechanism. Was PD-L1 testing performed? If so, report it. If not, acknowledge this gap.

We thank the reviewer for this important point. PD-L1 expression and lymphocyte infiltration were included as exploratory objectives in the original protocol. However, due to limited available tissue and practical constraints in this ultra-rare disease cohort, PD-L1 IHC was not successfully completed for all enrolled patients at the time of this analysis. We have therefore clarified this limitation in the revised manuscript and avoided drawing conclusions regarding PD-L1 status.

Reviewer #3 (Remarks to the Author):

Summary:

This is a prospective phase II study tislelizumab plus lenvatinib for patients with FH deficient metastatic renal cell carcinoma. The study reached planned accrual of 20 patients. The combination achieved the primary endpoint with a reported ORR of 95%, greatly exceeding the level of interest of 60% pre-specified. All patients were reported to have disease control. While the follow up is relatively short, the PFS and OS are likely unstable though appear impressive. The authors reported the use two primary circulating metabolites and correlation of response and progression. These were pre-specified and were impressive results.

This is the second reported ICI/TKI combination for FH-RCC building off a recently published single arm study of axitinib plus sintilimab. The activity is at minimum in line with this previously published data and adds a second study supporting the use of ICI/TKI for this rare tumor subtype.

The work supports the conclusions or claims though would recommend that the

authors refrain from stating their combination appears more impressive than axitinib plus sintlimab given the small, single arm nature of both studies and difficulties in cross trial comparisons.

The methodology appears sound, though would like the authors to comment on below questions.

I would recommend revision of the manuscript.

Introduction: would cite in line 87 the previous work supporting the metabolites of interest.

We appreciate the reviewer's reminder. We have now added the cited reference at the appropriate place and updated the reference list accordingly.

Methods in line 122: where there rules about re-escalation in terms of resolution to grade 1 or limit of grade that would allow future re-escalation?

We apologize that the detailed dose adjustment and re-escalation rules for lenvatinib were inadvertently omitted from the previously uploaded English version of Protocol. Thanks to the reviewer's insightful comment, we have now added a full description of these rules in the revised Protocol as follows (Protocol Page 23):

"When a grade ≥3 or intolerable grade 2 AE considered possibly related to lenvatinib occurs, lenvatinib is temporarily withheld. Treatment could be resumed only after the AE resolves to grade 0-1 or baseline, at which point a dose reduction is required. Dose reduction is allowed stepwise from 20 mg to 16 mg, then to 12 mg, and then to 8 mg (20 mg → 16 mg → 12 mg → 8 mg). Skipping dose levels is not permitted. Subjects who could not tolerate the 8 mg dose would be suggested discontinuation from the study.

In principle, subjects who receive a dose reduction will continue on the reduced dose in subsequent cycles. However, while on a reduced dose, if the investigator judge that there would be a risk or possibility of disease progression, and the subject is considered stable for safety (i.e., no grade ≥3 AE or intolerable grade 2 AE), dose re-escalation by one level (i.e., +4 mg, no skipping) is allowed. Each subject is permitted at most one dose re-escalation. If, after re-escalation, the subject again develops a grade ≥ 3 or intolerable grade 2 AE, the protocol-mandated dose interruption is applied, and no further dose re-escalation is allowed after subsequent dose reduction."

Accordingly, dose re-escalation is permitted only when the patient on the reduced dose is considered "stable for safety", defined as no grade ≥3 or intolerable grade 2 AE. Intolerable grade 2 AEs are prospectively defined (e.g., those that significantly impair daily activities or require dose interruption despite optimal supportive care). If a patient again develops grade ≥3 or intolerable grade 2 AEs after re-escalation, they are managed with protocol-mandated dose interruption, and no further re-escalation is allowed after subsequent dose reduction. These rules fully address the reviewer's concerns regarding the resolution grade and the upper limit of toxicity that would allow future re-escalation. We have added the details to the manuscript accordingly.

In methods would discuss how follow up was calculated, would suggest reverse Kaplan Meier technique.

We thank the reviewer for this constructive suggestion. We admit that in the original manuscript we did not correctly describe the method for calculating follow-up time. We simply reported the crude median follow-up duration without using the reverse Kaplan-Meier technique, which is indeed the appropriate method for estimating median follow-up time in time-to-event analyses.

Based on the reviewer's advice, we have now re-analyzed the data using the reverse Kaplan-Meier method (i.e., treating patients who experienced the endpoint event as censored and those alive/event-free at last follow-up as events). The median follow-up time calculated by this method was 20.2 months (95%CI 14.6–23.9).

We have added a clear description of this method in the revised Patients and Methods Section and reported the results in the Results Section. We appreciate the reviewer's guidance, which has improved the methodological accuracy of our manuscript.

In sample size calculation in the previous retrospective series ICI/TKI had an ORR of approximately 60%, the previous study with bevacizumab plus erlotinib had an ORR of 72%. Please explain how the authors assumed a response rate of 20-30% to calculate sample size? If using historical data with single agent TKI this would be reasonable to consider.

We thank the reviewer for this careful reading. However, we would like to respectfully clarify a factual misunderstanding.

In our previous retrospective study (*Eur Urol.* 2023 Feb;83(2):163–172.PMID:

35715365), 67 patients were evaluable for response to first-line systemic therapy with Bevacizumab/Erlotinib (n = 12), TKI monotherapy (n = 29), or ICI/TKI (n = 26). Best ORR of 27% was observed for the ICI/TKI cohort versus 25% for the Bev/Erlo cohort and 10% for the TKI monotherapy cohort. At the time we designed and initiated this clinical study, the results of the clinical trial of bevacizumab/erlotinib for advanced papillary renal cell carcinoma had only been reported at the ASCO-GU conference, while the results of the clinical trial of axitinib/sintilimab for advanced FH-RCC had not yet been publicly published. Therefore, the 25–30% response rate we used as the null hypothesis (p_0=0.30) for sample size calculation is exactly the one observed in our own prior real-world retrospective cohort of patients treated with first-line ICI/TKI or bevacizumab/erlotinib, which was published in 2023. In other words, we used precisely the type of historical data that the reviewer agrees would be reasonable.

We apologize if the original presentation was ambiguous. To avoid any confusion, we have now revised the sample size calculation section in the manuscript to more clearly state that, by adding citation, the null hypothesis ORR of 30% was based on our own retrospective data showing 25–30% ORR for ICI/TKI in the same treatment setting. Prospective studies led by Dr. Linehan from the U.S. National Cancer Institute (*N Engl J Med.* 2025 Jun 19;392(23):2346-2356) and by Dr. Zeng from West China Hospital (*JAMA Oncol.* 2025 Oct 1;11(10):1169-1177) reported response rates of 72% and 56% respectively, which were both published in 2025.

We appreciate the reviewer’s opportunity to clarify this point.

Results:

Line 198-199 would state length of SD (assume greater than 24 weeks) given 100% DCR

We thank the reviewer for the suggestion. We have reviewed the medical records of the single patient whose best response was stable disease (SD). The duration of SD for this patient was 4.4 months. The short duration was due to progression of non-target bone metastases at the subsequent imaging assessment, which defined the end of SD and converted the overall response to progressive disease (PD). No other patient had SD as best response; all others achieved partial or complete response. We have added this information to the Results section.

We thank the reviewer for improving the completeness of our data reporting.

Line 223: the rate of grade 3 adverse events is much lower than previous studies with lenvatinib 20mg, would recommend reporting all adverse events and then treatment related adverse events.

We thank the reviewer for the opportunity to clarify both the numerical comparison and the adverse event reporting approach.

First, we respectfully note that the incidence of grade≥3 treatment-related adverse events (TRAEs) in our study (45%) was not substantially lower than that reported in the key reference study (KEYNOTE-B61). In KEYNOTE-B61 study, the rate of grade 3-4 TRAEs was 51% in the initial report (81/158) (***Lancet Oncol.*** 2023 Aug;24(8):881-891. PMID: 37451291) and 58% in the updated 2-year analysis (92/158) (***Eur Urol.*** 2025 Dec;88(6):614-624. PMID: 40707309). The difference between our observed rate and these figures is modest and likely attributable to the relatively small sample size of our cohort and limited follow-up, which can lead to proportional variability. Therefore, we believe the description "much lower" may overstate the actual difference.

Second, regarding the suggestion to report all AEs separately from TRAEs, we would like to share the following considerations. In our study, all enrolled patients experienced at least one AE that was considered treatment-related; consequently, the distinction between "all AEs" and "TRAEs" is essentially absent. Moreover, the main safety tables in the KEYNOTE-B61 publication (PMID: 37451291, Table 3), bevacizumab/erlotinib publication (PMID: 40532152, Table 3) and also axitinib/sintilimab publication (PMID: 40810951, Table 2), all focused exclusively on TRAEs, which is a widely accepted practice in oncology trials. The CONSORT Harms 2022 statement acknowledges that when causal attribution is challenging or when all events are deemed treatment-related, reporting TRAEs as the primary safety summary is acceptable, provided the methods are transparent.

Nonetheless, if the editor and reviewer strongly prefer a separate listing of all observed AEs (regardless of attribution), we are happy to provide such a table as supplementary material. We would, however, prefer to keep the main manuscript aligned with the reporting standard of KEYNOTE-B61, which we consider appropriate for this class of combination therapy. We thank the reviewer again for the careful reading and constructive suggestions.

Line 234: what proportion of patients had re-escalation of lenvatinib as allowed in protocol?

We thank the reviewer for this question. We have reviewed the medical records

of all enrolled patients. Two out of 20 patients (10%) underwent dose re-escalation of lenvatinib as permitted by the protocol. The details are as follows:

Both patients had initially required dose reduction to 8 mg (the lowest allowed dose) due to TRAEs: one due to grade 3 arthralgia and the other due to intolerable grade 2 palmar-plantar erythrodysesthesia syndrome. Subsequently, because of imaging-documented fluctuation of target lesions suggesting potential PD risk, both patients were re-escalated by one dose level (i.e., from 8 mg back to 12 mg) according to the protocol-defined re-escalation rules and kept on this dose level. No other patients met the criteria for dose re-escalation. We have added this information to the Results-Safety Section as follows: "Dose re-escalation of lenvatinib (from 8 mg to 12 mg) was performed in 2 patients (10%) due to imaging-documented lesion fluctuation after initial dose reduction for grade 3 arthralgia or intolerable grade 2 palmar-plantar erythrodysesthesia."

We thank the reviewer for improving the completeness of our dose modification reporting.

Is there any information about dose density? How many breaks occurred per patient, what was the average dosing of patients treatment?

We thank the reviewer for these important questions. We agree that dose density and detailed break information are valuable for interpreting tolerability and efficacy. However, due to the nature of this investigator-initiated trial without an electronic data capture (EDC) system, all dosing records were manually documented in outpatient medical charts. While we were able to identify the number of treatment interruptions (breaks) per patient from the clinical notes, the exact duration (days) of each break was not consistently recorded, making it impossible to accurately calculate dose density (e.g., mg/week delivered or relative dose intensity). What we can provide include: (1) number of breaks per patient: median 1 (range 0–2); (2) distribution of lenvatinib doses at the end of follow-up (already reported in the manuscript): 4 patients on 20 mg/day, 2 patients on 16 mg/day, and 10 patients on 12 or 8 mg/day. This reflects the long-term results of dose adjustments made during the study.

We acknowledge the lack of precise dose-density data as a limitation of our study. We appreciate the reviewer's understanding of the practical constraints in investigator-initiated trials without dedicated data capture

infrastructure.

Line 236: what were the immune related adverse events that require high dose steroids? What event led to treatment discontinuation of the immunotherapy?

We thank the reviewer for asking for this clarification. Based on CRF records, we can now provide a more detailed answer.

In our cohort, two patients (10%) required high-dose steroids (⩾40 mg prednisone equivalent). Both of these patients developed immune-related pneumonitis. One of them had grade 3 severe immune-related pneumonitis that led to permanent discontinuation of tislelizumab. The other patient with pneumonitis recovered after steroid treatment and resumed immunotherapy thereafter. The third patient who had tislelizumab temporarily held (but not permanently discontinued) experienced adrenal insufficiency and required hydrocortisone supplement.

We have revised the manuscript accordingly to explicitly state: "Tislelizumab was temporarily held in 3 patients (15%) due to immune-related AEs (pneumonitis in 2 patients, adrenal insufficiency in 1 patient). High-dose steroids (⩾40 mg prednisone equivalent) were required in both patients with pneumonitis, one permanently discontinued tislelizumab because of severe immune-related pneumonitis and the other one recovered after steroid therapy and resumed immunotherapy thereafter. The patient with adrenal insufficiency required hydrocortisone supplement".

We appreciate the reviewer's attention to safety reporting.

Discussion

Line 273 would move up the pneumonitis to the results section.

We thank the reviewer for the suggestion. Upon reviewing the manuscript, we confirmed that the Results Section already contained description of the immune-related pneumonitis case. Therefore, the sentence at Line 273 in the Discussion was redundant.

We have deleted the redundant sentence at Line 273 to avoid repetition and improve clarity. The Results section now remains the sole location for this safety outcome, as recommended by the reviewer. We appreciate the reviewer's attention to manuscript organization.

Line 277: add citation

We thank the reviewer for the reminder. The missing citation has been added at Line 277 in the revised manuscript.

Line 288: Would recommend moving the discussion of the JAMA Oncology manuscript of axitinib plus sintilimab in FH_RCC doi:10.1001/jamaoncol.2025.2497, and discuss similarities of findings to this area of the discussion

We thank the reviewer for this helpful suggestion. We have now restructured the Discussion section, moving the discussion of the cited JAMA Oncology article (DOI: 10.1001/jamaoncol.2025.2497) to a more suitable location, where the similarities between our findings and that study are discussed in better sequence. We appreciate the reviewer’ s constructive input.

Line 289 histopathology evaluation was part of pre-planned correlative analysis this needs to move to results

We thank the reviewer for this careful reading. We realize that the original wording on Line 289 was ambiguous and may have led the reviewer to believe that we performed histopathology evaluation (e.g., IHC for lymphocyte markers and PD-L1) as part of our own pre-planned correlative analysis. In fact, that sentence was intended to cite previously published literature describing the characteristic histopathological features of FH-RCC (abundant lymphocyte infiltration and positive PD-L1 expression), not to report original data from our own study. We did not perform such immunostaining in our patients. Therefore, this information belongs to the Discussion rather than the Results section. To avoid confusion, we have revised the sentence to clearly indicate that these findings are from prior studies (citations retained). The revised sentence now reads: “Previous studies have shown that FH-RCC typically exhibits abundant lymphocyte infiltration and positive PD-L1 expression.” We apologize for the lack of clarity and thank the reviewer for the opportunity to improve our manuscript.

Line 294 this should move to the introduction to discuss how you decided about pre-specified ICI/TKI response rate. This appears very different than a different retrospective analysis. Liang, J., Sun, G., Pan, X. et al. Genomic and transcriptomic features between primary and paired metastatic fumarate hydratase–deficient renal cell carcinoma. Genome Med 15, 31 (2023). https://doi.org/10.1186/s13073-023-01182-7 which reported much higher ICI/TKI responses. Any reason to believe the response to bevacizumab plus erlotinib would be so much lower in the cited article as compared to the

prospective study? Were more patients somatic biallelic loss?

We thank the reviewer for the insightful comments and questions.

First, about Line 294 (pre-specified ICI/TKI response rate). We agree that this sentence is better placed in the Introduction or Method section to explain how the pre-specified response rate was determined for our study design. We have moved it to the appropriate position (Patient and Methods section) accordingly.

Second, about the discrepancy with the retrospective study by Liang et al. (*Genome Med.* 2023 May 2;15(1):31. PMID: 37131267). The reviewer notes that Liang et al. reported a higher ICI/TKI ORR of 47% (8/17) in a single-center retrospective analysis, whereas our previous multicenter retrospective study (*Eur Urol.* 2023 Feb;83(2):163-172.PMID: 35715365) showed an ORR of 27% (7/26). Given the small sample sizes in both studies (26 vs. 17 patients), we believe this numerical difference should be interpreted with caution and does not necessarily indicate a true biological or clinical disparity.

Third, about the lower ORR of bevacizumab plus erlotinib in our retrospective study (*Eur Urol.* 2023 Feb;83(2):163-172.PMID: 35715365) compared with the prospective trial (*N Engl J Med* 2025; PMID: 40532152). The reviewer raises two important points: whether real-world factors or a higher proportion of patients with somatic biallelic FH loss could explain the lower ORR. We fully agree with both possibilities.

(1) Real-world bias. In China, bevacizumab and erlotinib have not been approved for advanced RCC; their use in metastatic RCC is entirely off-label. Consequently, the retrospective study inevitably included heterogeneous dosing schedules and treatment protocols, whereas the prospective trial followed a strict protocol. Indeed, in the same retrospective study, the ORR for ICI/TKI combinations (27%) was also substantially lower than that reported in prospective ICI/TKI trials (e.g., sintilimab plus axitinib in *JAMA Oncol* 2025). Therefore, direct comparison between real-world retrospective data and highly selected prospective trial data may be inappropriate.

(2) Mutation type. The reviewer's hypothesis is plausible. The prospective trial of bevacizumab plus erlotinib enrolled only patients with germline *FH* mutations (HLRCC). In contrast, our retrospective study included both patients with germline *FH* mutations and those with somatic biallelic *FH*-inactivating mutations. Subgroup analyses from two prospective studies, sintilimab plus axitinib (*JAMA Oncol.* 2025 Oct 1;11(10):1169-1177. PMID:

40810951) and our current study of tislelizumab plus lenvatinib, consistently showed numerically higher ORR and longer PFS in patients with germline mutations compared to those with somatic mutations (sintilimab/axitinib: ORR 63% vs. 43%, median PFS 32.0 vs. 12.6 months; tislelizumab/lenvatinib: ORR 100% vs. 80%, median PFS 24.8 vs. 8.7 months). Although sample sizes were limited and definitive conclusions cannot be drawn, these data suggest that patients with somatic biallelic loss might possibly respond less favorably to systemic therapy. Thus, the inclusion of somatic-mutant patients in our retrospective cohort is a likely contributor to the lower ORR observed with bevacizumab plus erlotinib compared with the germline-only prospective trial.

We have added these considerations to the Discussion section and thank the reviewer for prompting us to clarify these important issues.

Line 303 would remove cross trial declaration of numerical superiority of your combination, given the small sample size and the wide overlapping confidence interval.

We thank the reviewer for the comment. We fully agree that with the small sample size and overlapping confidence intervals, any cross-trial claim of numerical superiority is not justified. We have removed that statement from line 303 and revised the wording accordingly to a more neutral description.

Line 330 strong part of discussion. Would comment on differences detected between germline and somatic biallelic alteration of circulating markers. If no difference would comment.

We thank the reviewer for the positive comment on this section of the Discussion and for the constructive suggestion. We agree that it would be valuable to comment on whether there is any difference in circulating metabolic markers between patients with germline *FH* mutations and those with somatic biallelic alterations. In our study, we examined the longitudinal changes in circulating succinyl-adenosine and succinic-cysteine during treatment and their correlation with radiographic tumor burden in the five patients with somatic mutations (P01, P03, P10, P11, P14). From visual inspection of the individual trajectories, the patterns appeared broadly similar to those observed in patients with germline mutations. However, given the very small number of somatic-mutant cases, we were unable to perform robust quantitative or statistical comparisons. Therefore, we cannot draw definitive conclusions regarding whether significant differences exist between the two mutation groups; we can only state that, based on the limited

available data, there was no obvious difference in trends.

In response to the reviewer’s suggestion, we have added a sentence in the Discussion acknowledging this observation and the limitation of small sample size. The revised text now states: ″In the five patients with somatic biallelic FH alterations, the longitudinal changes in circulating metabolic markers appeared similar to those in germline mutation carriers, but the small number precludes definitive statistical comparison. Larger studies are needed to clarify whether mutation type influences metabolic marker dynamics.″

We appreciate the reviewer’s thoughtful comment, which has improved the completeness of our discussion.

Table 3: Would add a line for total % of patients with AE and TRAE

Regarding the suggestion to report all AEs separately from TRAEs, we would like to share the following considerations. In our study, all enrolled patients experienced at least one AE that was considered treatment-related; consequently, the distinction between “all AEs” and “TRAEs” is essentially absent. Moreover, the main safety tables in the KEYNOTE-B61 publication (PMID: 37451291, Table 3), bevacizumab/erlotinib publication (PMID: 40532152, Table 3) and also axitinib/sintilimab publication (PMID: 40810951, Table 2), all focused exclusively on TRAEs, which is a widely accepted practice in oncology trials. The CONSORT Harms 2022 statement acknowledges that when causal attribution is challenging or when all events are deemed treatment-related, reporting TRAEs as the primary safety summary is acceptable, provided the methods are transparent. Nonetheless, if the editor and reviewer strongly prefer a separate listing of all observed AEs (regardless of attribution), we are happy to provide such a table as supplementary material. We would, however, prefer to keep the main manuscript aligned with the reporting standard of KEYNOTE-B61, which we consider appropriate for this class of combination therapy. We thank the reviewer again for the careful reading and constructive suggestions.

Figure 2: the patient with SD is much lower than 30% cut line, why was this patient SD, was this initial response or best response?

Thank you very much for this comment. In Figure 2, the response categories (CR, PR, SD) represented the best overall response (BOR) according to RECIST 1.1, rather than the initial or final response status. For the patient indicated by reviewer, the sum of target lesion diameters decreased from 114

mm at baseline to 90 mm at both the first and second follow-up assessments (approximately − 21.1%), which did not meet the threshold for partial response (PR, ≥30% decrease). Therefore, the best response achieved during these time points was classified as stable disease (SD). At the third follow-up, although the sum of target lesions further decreased to 25 mm (which would meet PR criteria based on size reduction alone), multiple new bone metastases were detected simultaneously. According to RECIST 1.1, the appearance of new lesions defines progressive disease (PD), regardless of target lesion shrinkage. Therefore, the result was classified as PD and does not change the previously determined BOR.

To improve transparency, we have now included in the revised manuscript representative imaging panels (Supplemental Figure Panel S3) for each patient at baseline, maximum response, and confirmed progression. All details mentioned above about this patient (P01) can be checked in the supplemental materials.

Regarding the waterfall plot, the bar reflects the maximum percentage reduction in target lesion size, while the response category reflects BOR based on comprehensive RECIST 1.1 evaluation, including both target lesions and new lesions. To avoid confusion, we have revised Figure 2 by explicitly adding the label "Best change from baseline, %" to clarify that the response categories (CR, PR, SD) represent the best overall response according to RECIST 1.1.
